# Asymmetric synthesis of γ-chiral borylalkanes *via* sequential reduction/hydroboration using a single copper catalyst†

**DOI:** 10.1039/d0sc03759a

**Published:** 2020-08-06

**Authors:** Jung Tae Han, Jin Yong Lee, Jaesook Yun

**Affiliations:** Department of Chemistry, Institute of Basic Science, Sungkyunkwan University Suwon 16419 Korea jaesook@skku.edu

## Abstract

The synthesis of γ-chiral borylalkanes through copper-catalyzed enantioselective S_N_2′-reduction of γ,γ-disubstituted allylic substrates and subsequent hydroboration was reported. A copper–DTBM-Segphos catalyst produced a range of γ-chiral alkylboronates from easily accessible allylic acetate or benzoate with high enantioselectivities up to 99% ee. Furthermore, selective organic transformations of the resulting γ-chiral alkylboronates generated the corresponding γ-chiral alcohol, arene and amine compounds.

## Abstract 2

Copper-catalyzed reductive hydroboration of γ,γ-disubstituted allylic substrates enables preparation of γ-chiral alkylboron compounds in a one-pot cascade manner.

## Graphical abstract

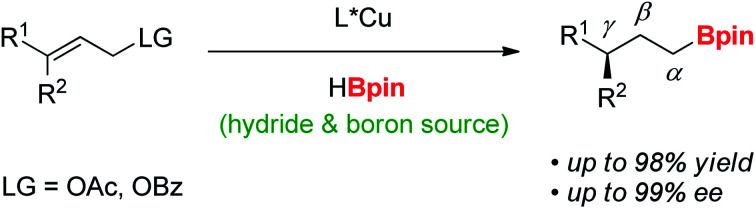

## Introduction

**Fig. 1:**
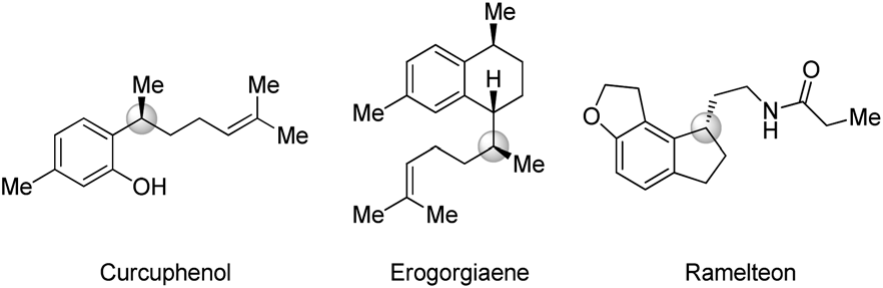
Representative functionalized γ-chiral compounds.

**Scheme 1:**
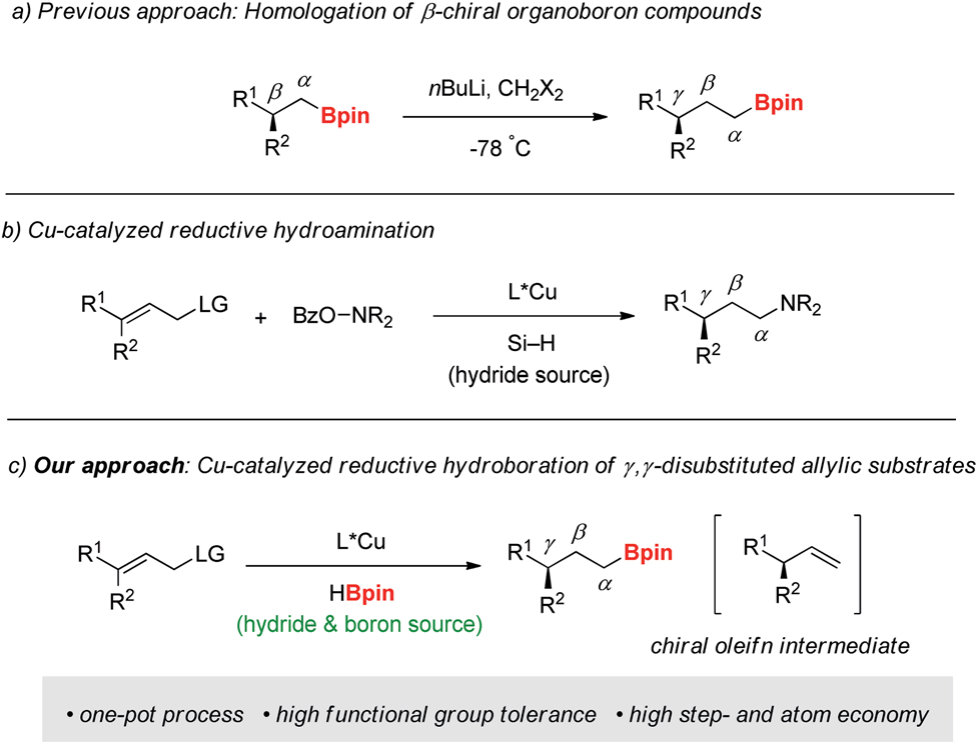
Approaches to γ-chiral organoboron compounds.

Efficient synthesis of enantiopure molecules with a stereogenic center remote from a functional group is of great interest in synthetic and medicinal chemistry, despite the difficulty of introducing such stereogenic centers.^1^ Especially, functionalized γ-chiral compounds represent important structural motifs in a diverse range of biologically active natural products and pharmaceutical drugs such as a marine natural product (curcuphenol) having inhibitory H,K-ATPase activity, an antimycobacterial agent (erogorgiaene) and a sleep agent (Ramelteon) (Fig. 1).^2^ In this context, γ-chiral organoboron compounds are valuable building blocks for the synthesis of functionalized chiral molecules due to efficient conversion of the carbon–boron bond to a range of carbon–carbon and carbon–heteroatom bonds.^3^ A typical approach towards γ-chiral organoborons is Matteson's homologation of enantioenriched β-chiral organoboranes with stoichiometric organolithium reagents (Scheme 1a).^4^ Despite the importance of these molecules, the direct preparation of γ-chiral organoboron compounds from easily accessible prochiral substrates remains unexplored in comparison with well-established methods for constructing α- and β-chiral organoboron compounds.^5^

Transition-metal catalyzed allylation is one of the most efficient and reliable tools for the synthesis of functionalized chiral molecules owing to facile construction of new stereogenic centers with simultaneous introduction of a versatile olefin fragment.^6^ Among the various methods, copper-catalyzed allylations have been widely explored with a range of organometallic nucleophiles such as Grignard, organolithium, organoboron, and organozirconium reagents.^7^ More recently, organocopper nucleophiles, catalytically *in situ* generated from unsaturated substrates, have been utilized in copper-catalyzed C–C bond formation reactions.^8^ Despite these significant advances, use of a hydride nucleophile is still rare in the allylation. Only two examples of copper-catalyzed enantioselective allylic reduction with hydrosilane (Si–H) as the stoichiometric hydride source have been recently reported.^9^ One of them reported highly enantioselective S_N_2′-reduction/hydroamination in a one-pot sequence (Scheme 1b).^9*b*^

Recently, we reported copper-catalyzed enantioselective hydroborations of various olefins with pinacolborane (HBpin).^10,11^ While pinacolborane displayed higher efficiency for addition reactions to multiple bonds such as alkenes, alkynes, and carbonyl derivatives^10–12^ than hydrosilane in the presence of a copper catalyst, its reactivity toward substitution reactions is unknown to date. Moreover, in our previous study on the copper-catalyzed hydroboration, the reaction of allylic acetate with pinacolborane-derived copper-hydride catalyst gave only hydroboration product,^10*b*^ indicating high tendency of pinacolborane for hydroboration of alkenes.

Our ongoing interest in copper-catalyzed synthesis of chiral organoboranes^13^ led us to explore preparation of γ-chiral organoboron compounds. Based on a possible dual role of pinacolborane to serve both as reducing and borating reagent, we envisioned that chiral copper-hydride species generated from HBpin could catalyze enantioselective S_N_2′-reduction of γ,γ-disubstituted allylic substrate, and hydroboration of the chiral intermediate olefins could afford γ-chiral organoboron compounds in a single operation (Scheme 1c). Herein, we report a general route for synthesis of γ-chiral organoboranes through reductive hydroboration strategy.

## Results and discussion

**Fig. 2:**
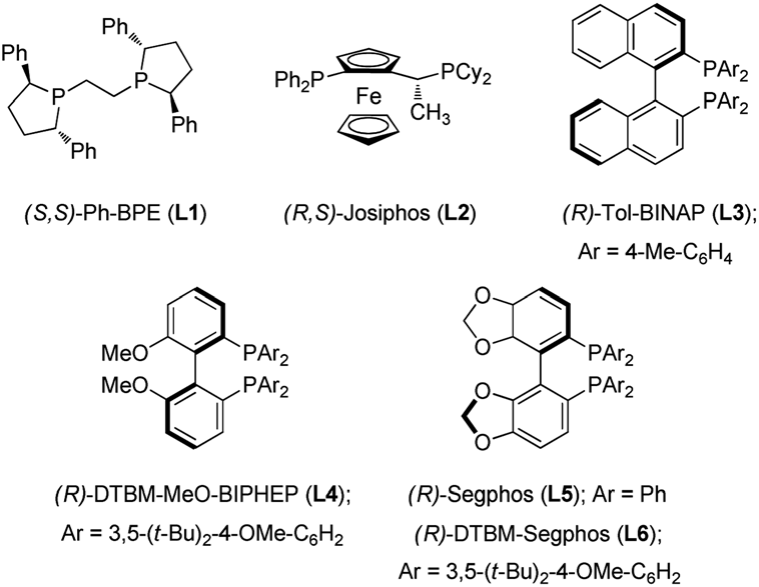
Structures of the chiral ligands.

In initial investigations, a series of chiral bisphosphine ligands (Fig. 2) were examined for reductive hydroboration of γ,γ-disubstituted allylic substrates (**1a**) derived from geraniol using pinacolborane (HBpin) (Table 1). Alkyl-tethered bisphosphine ligand **L1** and ferrocene-based bisphosphine ligand **L2** gave no desired product (entries 1 and 2). *C*_2_-symmetric tol-BINAP ligand **L3** showed no reactivity, but **L4** afforded the product in promising yield and with excellent enantioselectivity (entries 3 and 4). Although the Segphos (**L5**) did not provide the product, changing the ligand to DTBM-Segphos (**L6**) with its bulky aryl groups on the phosphine increased yield and enantioselectivity (entries 5 and 6).^10*b*–*d*,11*a*^ Next, we screened a range of leaving groups (LG) of **1a** for their effectiveness. Although use of allylic benzoate and carbonate afforded products in decreased yields, excellent enantioselectivities were conserved (entries 7 and 8). Allylic phosphate resulted in product in 60% yield and with 87% ee (entry 9), but allylic benzyl ether and bromide were inefficient (entries 10 and 11). Therefore, we chose acetate as the optimal leaving group, because it can be conveniently prepared from inexpensive acetic anhydride. Finally, prolongation of the reaction time to 24 h provided the product in 90% yield with retention of the high ee value (entry 12).

**Table 1:** Optimization of reaction conditions^a^

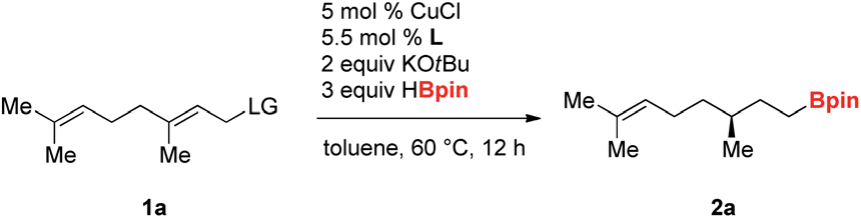				
Entry	Ligand	LG	Yield^b^ (%)	ee^c^
1	**L1**	OAc	0	—
2	**L2**	OAc	0	—
3	**L3**	OAc	0	—
4	**L4**	OAc	63	98
5	**L5**	OAc	0	—
6	**L6**	OAc	80	99
7	**L6**	OBz	75	97
8	**L6**	OCO_2_Me	59	99
9	**L6**	OP(O)(OEt)_2_	60	87
10	**L6**	OBn	0	—
11	**L6**	Br	0	—
12^d^	**L6**	OAc	90	99

a Reactions were conducted on 0.5 mmol scale of **1a**.

b Isolated yield.

c Determined by HPLC analysis on a chiral stationary phase.

d The reaction was carried out for 24 h.

With the optimized reaction conditions, the hydroboration of a range of γ,γ-disubstituted allylic substrates was investigated (Table 2). Allylic acetate derived from Nerol bearing a (*Z*)-olefin moiety was converted into **2b**, the enantiomeric product opposite to **2a** in high yield and enantioselectivity. Various functional groups were tolerated well, including chloro (**2d**), benzyl ether (**2e**), silyl ether (**2f**), and acetal group (**2g**) under the reaction conditions. While allylic acetate bearing a methyl and ethyl substituent on the γ-position underwent the reaction to afford highly enantioenriched alkylboronate (**2h**), the compounds (**1i**) bearing an ethyl and *n*-hexyl substituent resulted in drastically diminished yield and enantioselectivity. Bulky cyclohexyl (**1j**) and *tert*-butyl (**1k**) substituted allylic acetates were compatible and formed products in good yields and with excellent enantioselectivity. Similarly, silyl-substituted allylic acetate was converted into the γ-chiral silylalkylboronate (**2l**).

**Table 2:** Substrate scope in asymmetric reductive hydroboration^a^

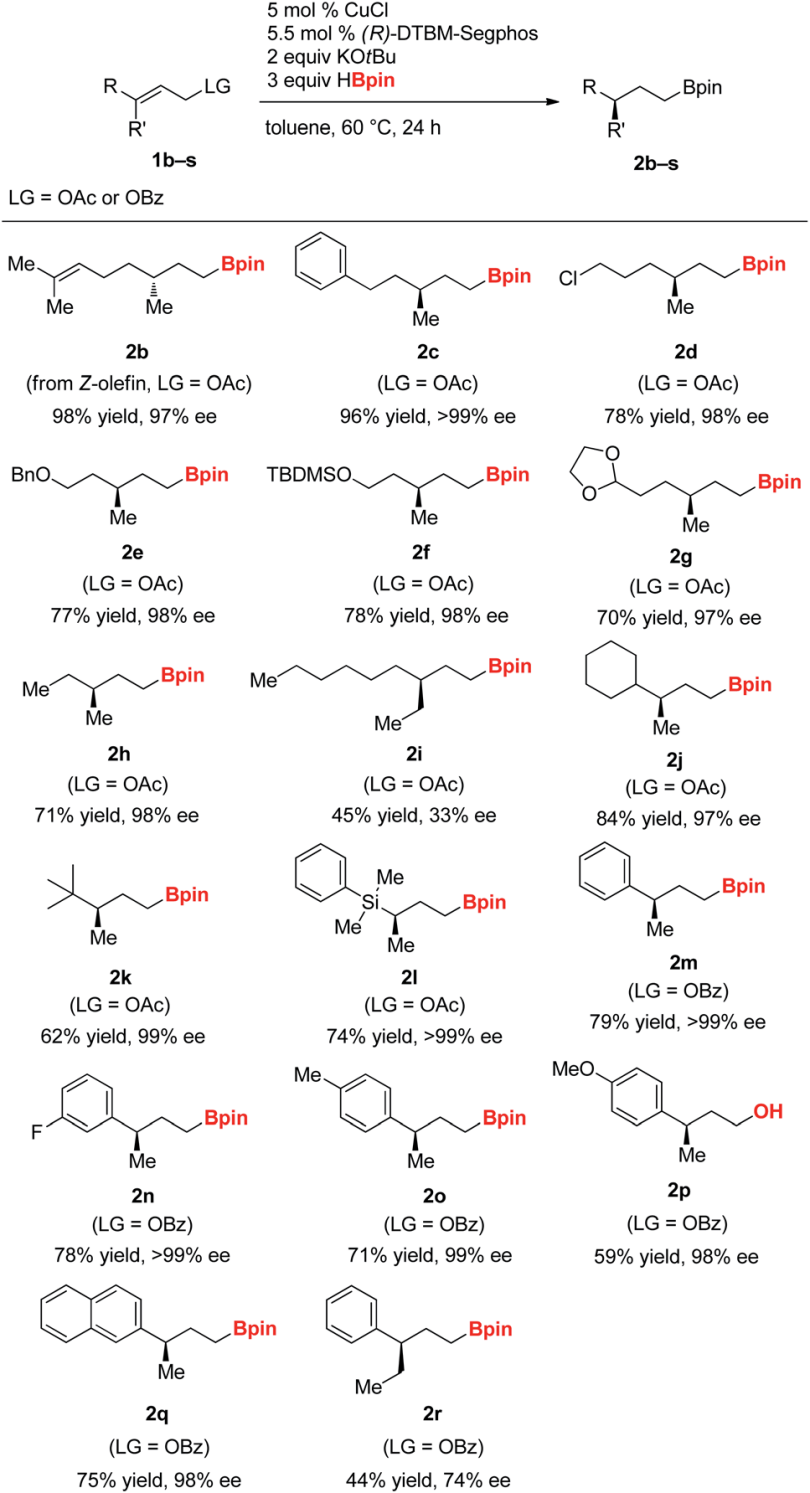

a Reactions were conducted on 0.5 mmol scale of **1**. ee values of **2** were determined by HPLC analysis on a chiral stationary phase.

Aryl-substituted allyl benzoates (**1m–1r**) efficiently underwent the hydroboration.^14^ Substrates bearing phenyl, 4-fluoro-phenyl, 4-tolyl, 4-methoxy-phenyl, and 2-naphthyl group were suitable for the reaction. However, allylic benzoate (**1r**) with a phenyl and ethyl substituent at the *γ*-position provided the desired product in diminished yield and enantioselectivity, possibly due to increased steric bulkiness at the reaction site. In addition, we found that allylic benzoates with a substituent at the C_α_ or C_β_ position were not efficient in yielding the desired products, probably due to enhanced steric hindrance around the olefin.^15^

**Scheme 2:**
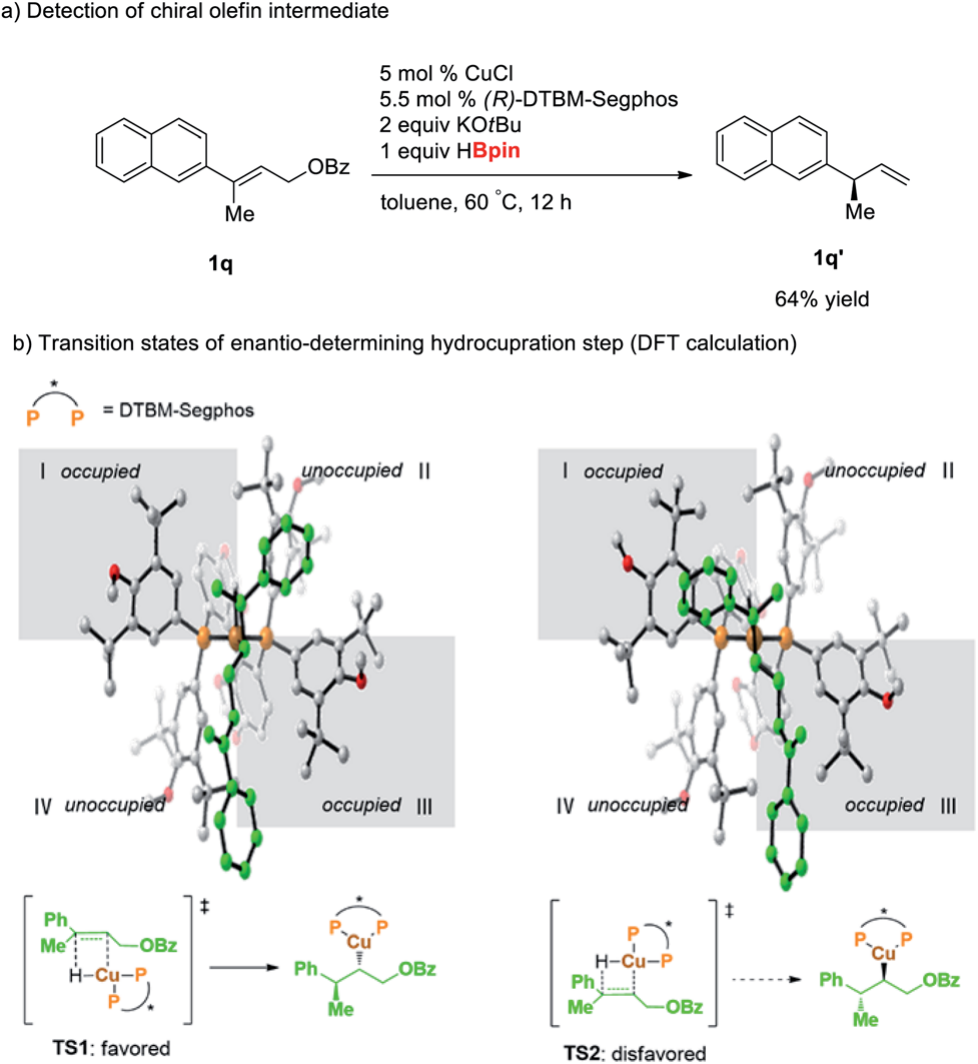
Mechanistic studies.

To examine the mechanism of the reductive hydroboration, we performed the reaction of **1q** with 1 equiv. of pinacolborane to observe the reaction intermediate (Scheme 2a). The reaction resulted in the formation of chiral olefin **1q′** in 64% yield without formation of further hydroboration product **2q**, indicating that this cascade reaction proceeds *via* rate-determining S_N_2′-reduction step followed by hydroboration. Moreover, DFT calculations of transition state for hydrocupration step of the allylic substrate **1m** revealed that the hydrocupration barrier for the major enantiomer is lower than that of the minor enantiomer by 4.6 kcal mol^−1^ (Scheme 2b).^16^ This energy difference of the transition states stems from steric repulsion between the phenyl substituent of **1m** and the bulky P substituents of the ligand **L6** (grey area **l** in the quadrant diagrams).

**Fig. 3:**
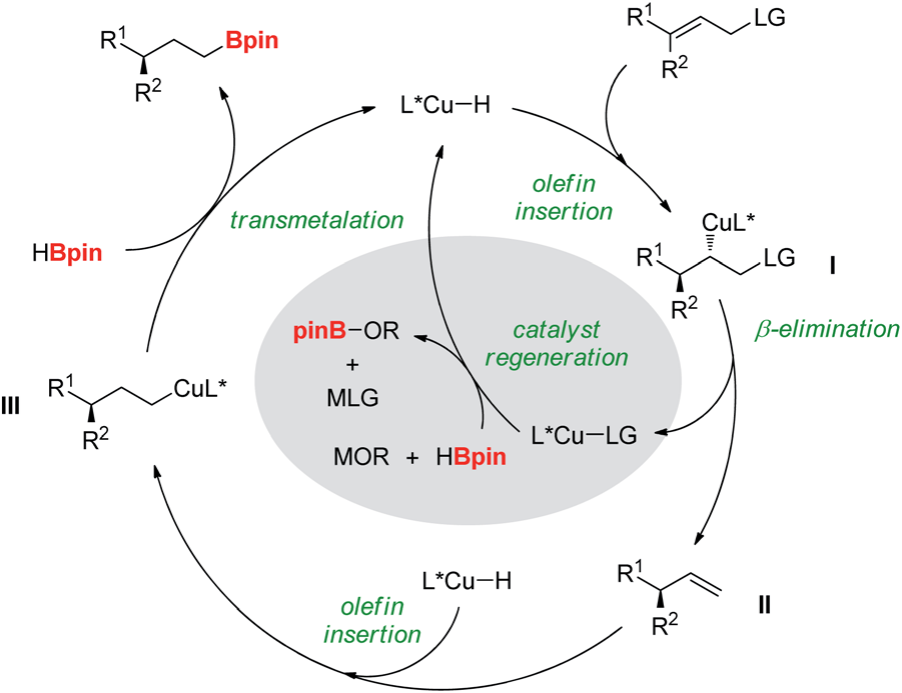
Proposed mechanism of copper-catalyzed reductive hydroboration.

Based on the mechanistic studies, we propose a catalytic cycle for the reductive hydroboration (Fig. 3). Copper–H addition to the allylic substrate would generate a chiral alkylcopper species **l**, which rapidly undergoes β-LG elimination to afford the chiral olefin intermediate **ll** and L*Cu–LG.^5*a*,17^ Subsequent addition of copper-hydride species, regenerated from the reaction of L*Cu–LG with pinacolborane and alkoxide base to **ll** would produce terminal alkylcopper intermediate **lll**. Finally, transmetalation of **lll** with pinacolborane would result in the formation of the desired product, releasing the copper-hydride species.

**Scheme 3:**
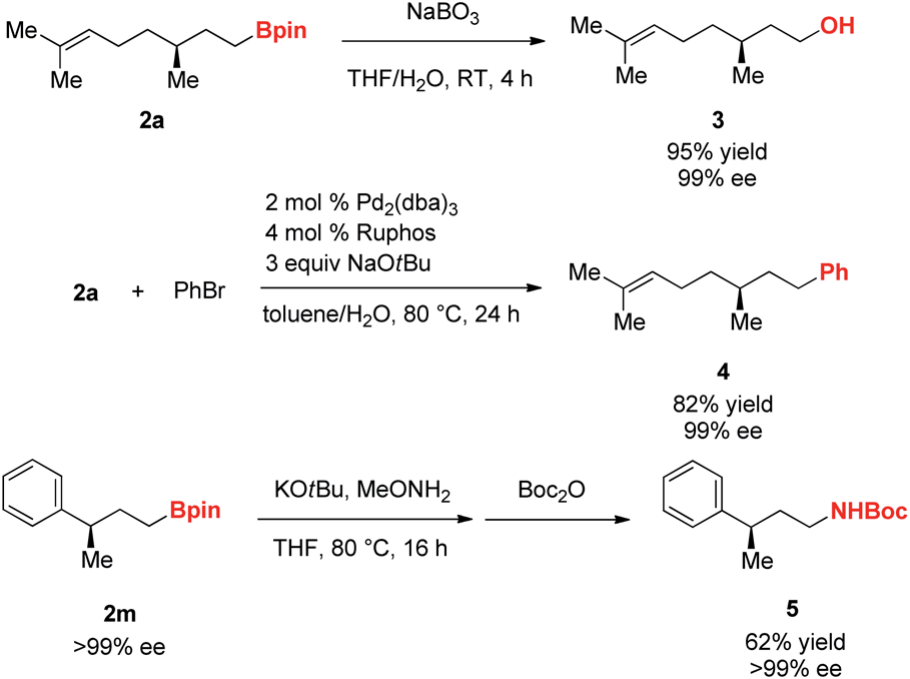
Application of γ-chiral alkylboron compounds.

Next, we examined applications of the resulting γ-chiral alkylboron compounds (Scheme 3). First, oxidation of **2a** with sodium perborate yielded (−)-citronellol **3**. Suzuki–Miyaura cross-coupling reaction of **2a** with an aryl bromide afforded the arylated product **4**.^18^ Furthermore, **2m** was transformed into the Boc-protected amine **5** through an amination and Boc protection.^19^

## Conclusion

In summary, we have described an efficient catalytic method for the synthesis of γ-chiral alkylboronates *via* S_N_2′-reduction and hydroboration. The DTBM-Segphos–copper complex successfully catalyzed the enantioselective allylic reduction of γ,γ-disubstituted allylic acetate (or benzoate) and subsequent hydroboration to produce γ-chiral alkylboronates in a one-pot cascade manner. This process provides a modular and general approach towards synthesis of γ-chiral organoboron compounds. Efforts to utilize a copper-hydride catalyst derived from pinacolborane in asymmetric synthesis are in progress.

## Conflicts of interest

There are no conflicts to declare.

## Supplementary Material

SC-011-D0SC03759A-s001
