# Supplementary material for: Asymmetric synthesis of γ-chiral borylalkanes via sequential reduction/hydroboration using a single copper catalyst
Source: Chem Sci. 2020 Aug 6;11(33):8961–5. doi: 10.1039/d0sc03759a (PMC8163415; doi:10.1039/d0sc03759a)
Supplement: SC-011-D0SC03759A-s001 [file SC-011-D0SC03759A-s001.pdf]

Electronic Supplementary Material (ESI) for Chemical Science.
This journal is © The Royal Society of Chemistry 2020

Supporting Information

Asymmetric Synthesis of *γ*–Chiral Borylalkanes via Sequential Reduction/Hydroboration using a Single Copper Catalyst

Jung Tae Han, Jin Yong Lee and Jaesook Yun*

Department of Chemistry and Institute of Basic Science

Sungkyunkwan University, Suwon 16419, Korea

Table of Contents

Ⅰ. General Methods	Page S1
Ⅱ. Experimental Details	Page S2–S40
Ⅲ. References	Page S41
Ⅳ. ^{1}H and ^{13}C NMR spectra	Page S42–S62

General Methods

CuCl, KO*t*Bu, pinacolborane, and other commercial reagents were purchased from Aldrich and used as received. *(R)*-DTBM-Segphos (**L6**) was purchased from TCI. **1a**–**1r** were prepared by following literature procedures.[1] Reactions with oxygen- and moisture-sensitive materials were carried out with the standard Schlenk technique. Toluene was purified using PureSolv solvent purification system, from Innovative Technology, Inc. Flash chromatography was performed on silica gel from Merck (70–230 mesh). All ^{1}H NMR spectra were obtained on Bruker at 500 systems and reported in parts per million (ppm) downfield from tetramethylsilane. ^{13}C NMR spectra are reported in ppm referenced to deuteriochloroform (77.16 ppm). Infrared spectra (IR) were obtained on Nicolet 205 FT-IR and were recorded in cm^{-1}. Optical rotation was measured with Model 343 plus polarimeter equipped with a sodium lamp (589 nm). High performance liquid chromatography (HPLC) was performed using Younglin Acme 9100 series. High resolution mass spectra (HRMS) were obtained at Korea Basic Science Institute (Cheongju, Korea) and reported in the form of *m/z* (intensity relative to peak = 100).

General procedure for the remote hydroboration of *γ*,*γ*-disbustituted allyic substrates

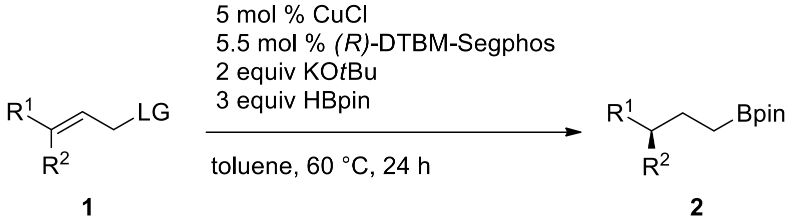

LG = OAc or OBz

A mixture of CuCl (5 mol %, 0.025 mmol), *(R)*-DTBM-Segphos (5.5 mol %, 0.0275 mmol) and KO*t*Bu (1 mmol) in anhydrous toluene (2 mL) were stirred for 5 min in a Schlenk tube under an atmosphere of nitrogen. Pinacolborane (1.5 mmol) was added to the reaction mixture and stirred for another 15 min at room temperature. Substrate **1** dissolved in toluene (1 mL) was added. The reaction mixture was sealed, stirred at 60 °C, and monitored by TLC. Upon completion of the reaction, the reaction mixture was diluted with diethyl ether (10 mL). After the aqueous layer was extracted with diethyl ether, the combined organic layers were dried over Na_2SO_4, and concentrated in vacuo. The product was purified by silica gel chromatography using hexanes/ethyl acetate as the eluent.

Determination of ee

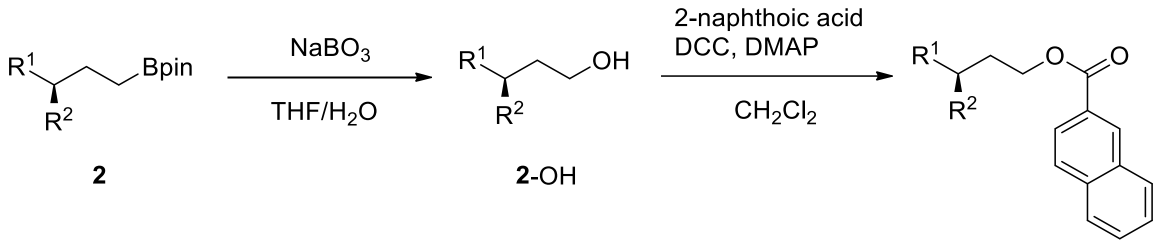

Sodium perborate (0.9 mmol) was added to **2** (0.3 mmol) in THF (2 mL) and water (2 mL). The reaction mixture was vigorously stirred for 4 h at room temperature. The reaction was quenched with water and then, extracted with diethyl ether. The combined organic layers were dried over Na_2SO_4 and concentrated in vacuo. The product was purified by silica gel chromatography.

To **2**-OH (1 equiv) in CH_2Cl_2 (3 mL) were added dicyclohexylcarbodiimide (1.05 equiv), 4-(dimethylamino)pyridine (0.25 equiv) and 2-naphthoic acid (1 equiv). The reaction mixture was stirred at room temperature and monitored by TLC. Upon completion of the reaction, the reaction mixture was purified silica gel chromatography.

Characterization of 2 (Table 1 and 2)

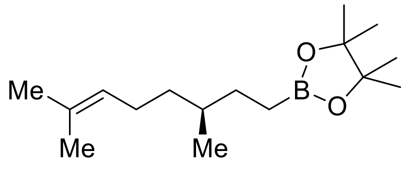

2a

***(R)*-2-(3,7-dimethyloct-6-en-1-yl)-4,4,5,5-tetramethyl-1,3,2-dioxaborolane:** By following the general procedure, **2a** was obtained in 90% yield. ^{1}H NMR (500 MHz, $CDCl_3$) δ 5.02 (t, J = 7.0 Hz, 1H), 1.94–1.84 (m, 2H), 1.60 (s, 3H), 1.52 (s, 3H), 1.40–1.33 (m, 1H), 1.30–1.23 (m, 3H), 1.17 (s, 12H), 1.06–1.02 (m, 1H), 0.78 (d, J = 6.0 Hz, 3H), 0.72–0.62 (m, 2H); ^{13}C NMR (125 MHz, $CDCl_3$) δ 130.9, 125.1, 82.8, 36.7, 34.6, 31.6, 30.9, 29.7, 25.7, 25.6, 24.8, 22.7; 19.1; 17.6; 14.1, 8.5 (C–B); IR (neat) 1460, 1365, 1320, 1140, 970 cm^{-1}; HRMS (ESI) calcd for [$C_{16}H_{31}BO_2$+H^+]: 266.2526, found: 266.2520; 99% ee were measured by chiral HPLC on AD-H column with the corresponding naphthoate derivative (*i*PrOH:hexane = 1:99, 0.5 mL/min); t_R = 14.75 min (major), t_R = 15.72 min (major).

[rac]

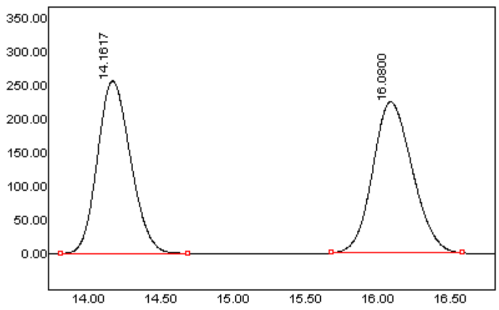

time (min) area area ratio

RT[분]	면적[mV*sec]	면적비[%]
14.1617	4082.9652	49.77
16.0800	4120.5590	50.23

[chiral]

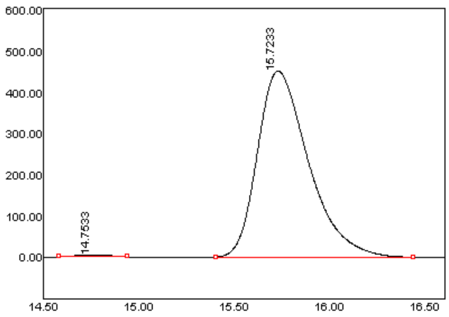

RT[분]	면적[mV*sec]	면적비[%]
14.7533	40.5508	0.48
15.7233	8491.1000	99.52

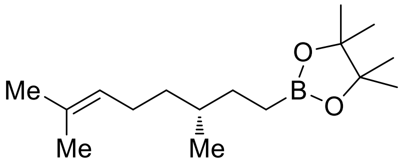

2b

***(S)*-2-(3,7-dimethyloct-6-en-1-yl)-4,4,5,5-tetramethyl-1,3,2-dioxaborolane:** By following the general procedure, **2b** was obtained in 98% yield. ^{1}H NMR (500 MHz, $CDCl_3$) δ 5.09 (t, *J* = 7.5 Hz, 1H), 2.01–1.91 (m, 2H), 1.67 (s, 3H), 1.60 (s, 3H), 1.46–1.41 (m, 1H), 1.36–1.27 (m, 3H), 1.24 (s, 12H), 1.13–1.09 (m, 1H), 0.85 (d, *J* = 6.5 Hz, 3H), 0.81–0.71 (m, 2H); ^{13}C NMR (125 MHz, $CDCl_3$) δ 130.9, 125.1, 82.8, 36.7, 34.6, 30.9, 25.7, 25.6, 24.9, 24.8, 19.1, 17.6; IR (neat) 1460, 1365, 1320, 1140, 970 cm^{-1}; HRMS (ESI) calcd for $[C_{16}H_{31}BO_2+H^+]$: 266.2526, found: 266.2520; 97% ee were measured by chiral HPLC on AD-H column with the corresponding naphthoate derivative (*i*PrOH:hexane = 1:99, 0.5 mL/min); t_R = 14.70 min (major), t_R = 16.02 min (major).

[rac]

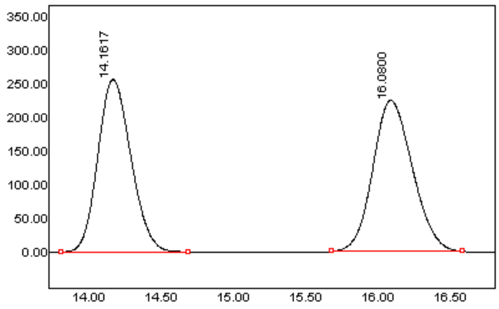

RT[분]	면적[mV*sec]	면적비[%]
14.1617	4082.9652	49.77
16.0800	4120.5590	50.23

[chiral]

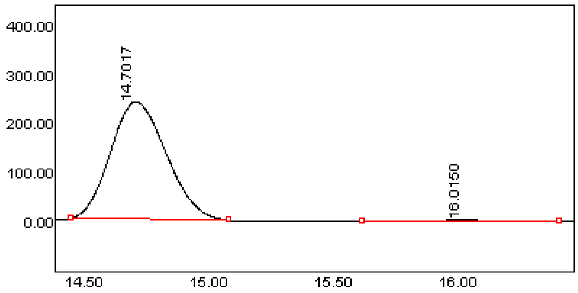

RT[분]	면적[mV*sec]	면적비[%]
14.7017	3747.3891	98.64
16.0150	51.5391	1.36

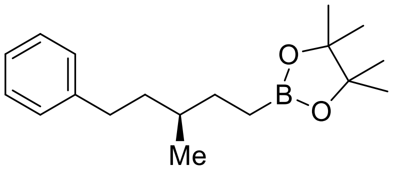

2c

***(R)*-4,4,5,5-tetramethyl-2-(3-methyl-5-phenylpentyl)-1,3,2-dioxaborolane:** By following the general procedure, **2c** was obtained in 96% yield. The characterization data for **2c** was concordant with that previously reported in the literature.[2] ^{1}H NMR (500 MHz, $CDCl_3$) δ 7.27–7.24 (m, 2H), 7.18–7.13 (m, 2H), 2.67–2.53 (m, 2H), 1.68–1.60 (m, 1H), 1.52–1.45 (m, 1H), 1.44–1.37 (m, 2H), 1.27–1.25 (m, 1H), 1.23 (s, 12H), 0.92 (d, *J* = 6.0 Hz, 3H), 0.83–0.71 (m, 2H); ^{13}C NMR (125 MHz, $CDCl_3$) δ 143.2, 128.4, 128.2, 125.5, 82.9, 38.5, 34.6, 33.5, 30.8, 24.8, 24.8, 19.1, 8.4 (C–B); >99% ee were measured by chiral HPLC on IA column with the corresponding alcohol (*i*PrOH:hexane = 5:95, 0.5 mL/min); t_R = 7.68 min (minor), t_R = 11.49 min (major).

[rac]

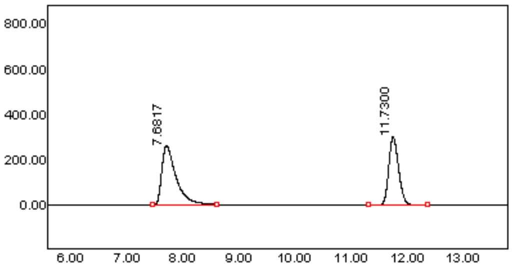

RT[분]	면적[mV*sec]	면적비[%]
7.6817	3626.1234	49.74
11.7300	3664.6992	50.26

[chiral]

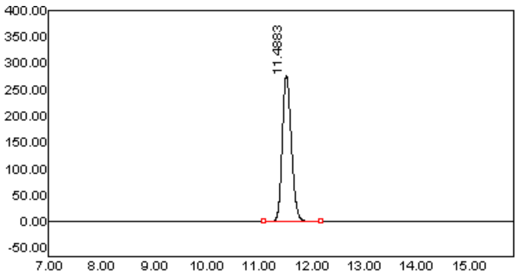

RT[분]	면적[mV*sec]	면적비[%]
11.4883	3338.0492	100.00

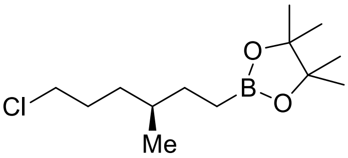

2d

***(R)*-2-(6-chloro-3-methylhexyl)-4,4,5,5-tetramethyl-1,3,2-dioxaborolane:** By following the general procedure, **2d** was obtained in 78% yield. The characterization data for **2d** was concordant with that previously reported in the literature.[2] ^{1}H NMR (500 MHz, $CDCl_3$) δ 3.53 (t, *J* = 7.0 Hz, 2H), 1.82–1.71 (m, 2H), 1.48–1.41 (m, 2H), 1.38–1.29 (m, 2H), 1.24 (s, 12H), 0.87 (d, *J* = 6.5 Hz, 3H), 0.81–0.71 (m, 2H); ^{13}C NMR (125 MHz, $CDCl_3$) δ 82.9, 45.5; 34.4, 33.8, 30.7, 30.3, 24.9, 24.8, 19.1; 98% ee was measured by chiral HPLC on OD-H column with the corresponding naphthoate derivative (*i*PrOH:hexane = 1:99, 0.5 mL/min); t_R = 28.26 min (major), t_R = 29.18 min (major).

[rac]

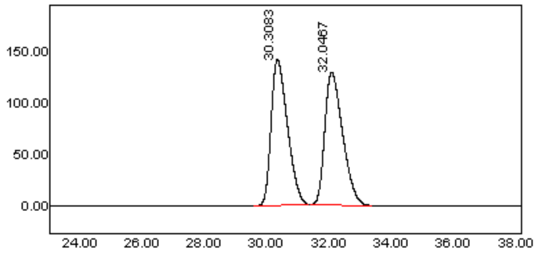

RT[분]	면적[mV*sec]	면적비[%]
30,3083	5197,0836	50,10
32,0467	5177,1141	49,90

[chiral]

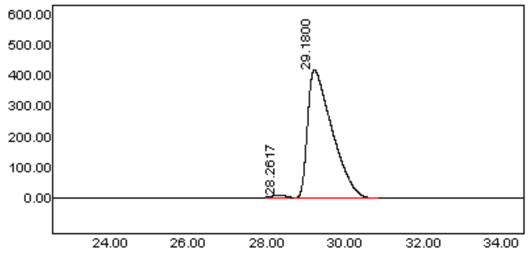

RT[분]	면적[mV*sec]	면적비[%]
28,2617	174,2427	0,95
29,1800	18095,2438	99,05

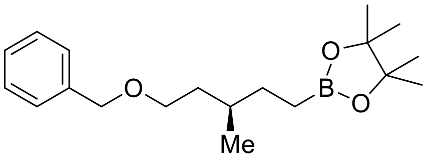

2e

***(R)*-2-(5-(benzyloxy)-3-methylpentyl)-4,4,5,5-tetramethyl-1,3,2-dioxaborolane:** By following the general procedure, **2e** was obtained in 77% yield. ^{1}H NMR (500 MHz, $CDCl_3$) δ 7.34–7.33 (m, 4H), 7.28–7.24 (m, 1H), 4.49 (d, *J* = 2.5 Hz, 2H), 3.54–3.47 (m, 2H), 1.72–1.65 (m, 1H), 1.53–1.38 (m, 3H), 1.27–1.26 (m, 1H), 1.24 (s, 12H); 0.87 (d, *J* = 6.5 Hz, 3H), 0.82–0.70 (m, 1H); ^{13}C NMR (125 MHz, $CDCl_3$) δ 138.8, 128.3, 127.6, 127.4, 82.9, 72.8, 68.9, 36.4, 32.0, 31.1, 24.8, 19.2, 8.7 (C–B); IR (neat) 2972, 1450, 1378, 1275, 1136 cm^{-1}; HRMS (ESI) calcd for [$C_{19}H_{31}BO_3$+H^+]: 319.2444, found: 319.2440; 98% ee was measured by chiral HPLC on AS-H column with the corresponding alcohol (*i*PrOH:hexane = 1:99, 0.5 mL/min); t_R = 45.64 min (minor), t_R = 48.69 min (major).

[rac]

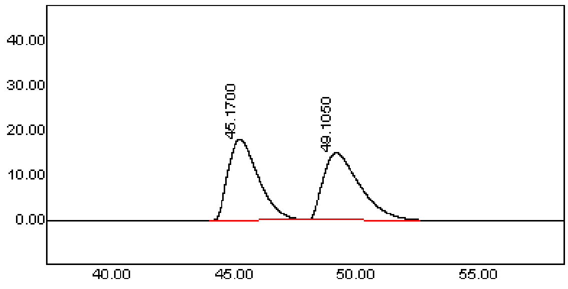

RT[분]	면적[mV*sec]	면적비[%]
45,1700	1559,2449	50,07
49,1050	1555,1926	49,93

[chiral]

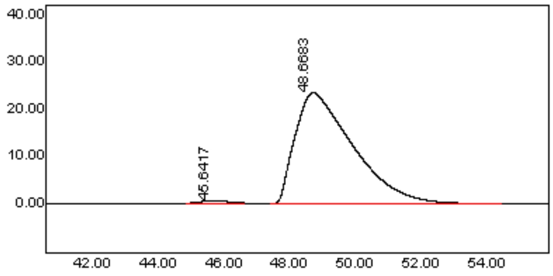

RT[분]	면적[mV*sec]	면적비[%]
45,6417	31,5001	1,08
48,6683	2877,5557	98,92

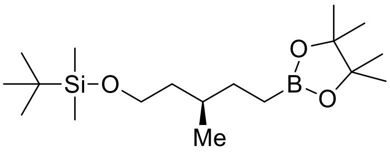

2f

***(R)*-tert-butyldimethyl((3-methyl-5-(4,4,5,5-tetramethyl-1,3,2-dioxaborolan-2-yl)pentyl)oxy)silane:** By following the general procedure, **2f** was obtained in 88% yield. ^{1}H NMR (500 MHz, $CDCl_3$) δ 3.57 (d, *J* = 7.0 Hz, 2H), 1.57–1.42 (m, 3H), 1.35–1.31

(m, 2H), 1.24 (s, 12H), 0.89 (s, 9H), 0.85 (d, J = 6.5 Hz, 3H), 0.80–0.70 (m, 2H); ^{13}C NMR (125 MHz, $CDCl_3$) δ 82.8, 63.7, 34.8, 32.6, 30.9, 30.5, 26.0, 24.9, 24.8, 19.2, 18.4; IR (neat) 2987, 1444, 1285, 1140 cm^{-1}; HRMS (ESI) calcd for $[C_{19}H_{41}BO_3Si+H^+]$: 357.2996, found: 357.2995; 98% ee was measured by chiral HPLC on OD-H column with the corresponding naphthoate derivative (*i*PrOH:hexane = 1:99, 1.0 mL/min); t_R = 13.49 min (minor), t_R = 15.89 min (major).

[rac]

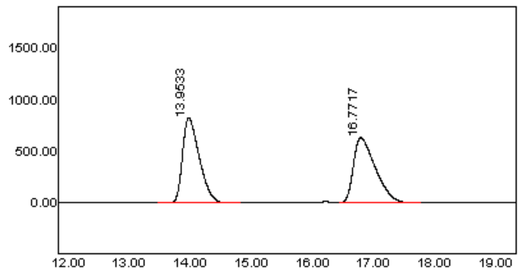

RT[분]	면적[mV*sec]	면적비[%]
13,9533	15760,7000	49,99
16,7717	15769,1813	50,01

[chiral]

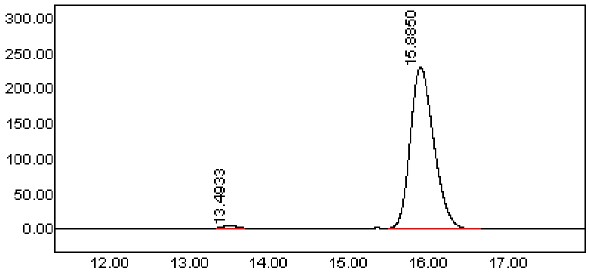

RT[분]	면적[mV*sec]	면적비[%]
13,4933	46,4950	0,97
15,8850	4722,8703	99,03

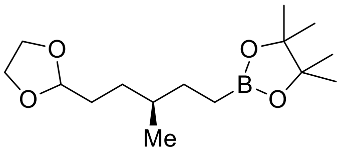

2g

***(R)*-2-(5-(1,3-dioxolan-2-yl)-3-methylpentyl)-4,4,5,5-tetramethyl-1,3,2-dioxaborolane:** By following the general procedure, **2g** was obtained in 70% yield. ^1H NMR (500 MHz, $CDCl_3$) δ 4.82 (t, J = 5.0 Hz, 1H), 3.97–3.92 (m, 2H), 3.87–3.82 (m, 2H), 1.71–1.62 (m, 2H), 1.26–1.21 (m, 15H), 0.87 (d, J = 6.5 Hz, 3H), 0.80–0.70 (m, 2H); ^{13}C NMR (125 MHz, $CDCl_3$) δ 105.0, 82.9, 64.8, 34.8, 31.5, 30.7, 30.6, 24.9, 24.8, 19.7; IR (neat) 2980, 1465, 1366, 1280, 1140 cm^{-1}; HRMS (ESI) calcd for $[C_{15}H_{29}BO_4+H^+]$: 285.2237, found: 285.2235; 97% ee was measured by chiral HPLC on AS-H column with the corresponding naphthoate derivative (*i*PrOH:hexane = 1:99, 1.0 mL/min); t_R = 12.53 min (major), t_R = 15.17 min (minor).

[rac]

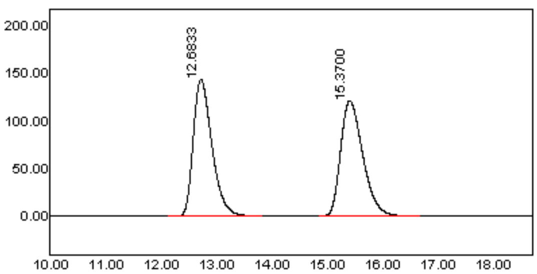

RT[분]	면적[mV*sec]	면적비[%]
12,6833	3374,1855	49,87
15,3700	3392,4441	50,13

[chiral]

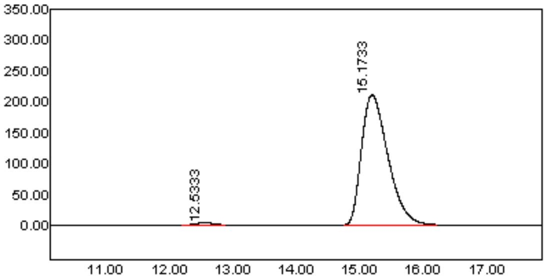

RT[분]	면적[mV*sec]	면적비[%]
12,5333	83,2931	1,35
15,1733	6078,1453	98,65

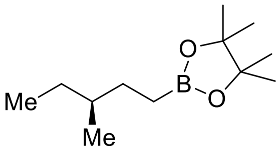

2h

***(S)*-4,4,5,5-tetramethyl-2-(3-methylpentyl)-1,3,2-dioxaborolane:** By following the general procedure, **2h** was obtained in 71% yield. ^{1}H NMR (500 MHz, $CDCl_3$) δ 1.47–1.37 (m, 2H), 1.36–1.29 (m, 2H), 1.24 (s, 12H), 1.13–1.09 (m, 1H), 0.87–0.83 (m, 6H), 0.79–0.70 (m, 2H); ^{13}C NMR (125 MHz, $CDCl_3$) δ 82.8, 36.6, 30.5, 29.0, 24.9, 24.8, 18.8, 11.5; IR (neat) 2980, 1735, 1370, 1236, 1042 cm^{-1}; HRMS (ESI) calcd for $[C_{12}H_{25}BO_2+H^+]$: 213.2025, found: 213.2030; 98% ee was measured by HPLC on OZ-H column with the corresponding naphthoate derivative (*i*PrOH:hexane = 1:99, 1.0 mL/min); t_R = 16.37 min (minor), t_R = 17.89 min (major).

[rac]

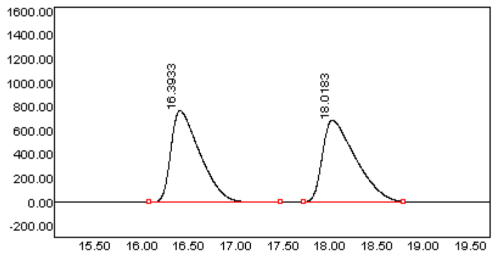

[chiral]

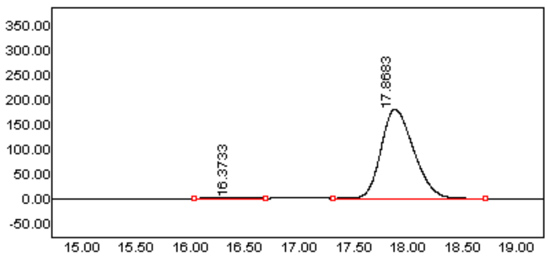

RT[분]	면적[mV*sec]	면적비[%]
16.3933	16568.0969	49.61
18.0183	16826.6266	50.39

RT[분]	면적[mV*sec]	면적비[%]
16.3733	33.4609	0.89
17.8683	3744.1039	99.11

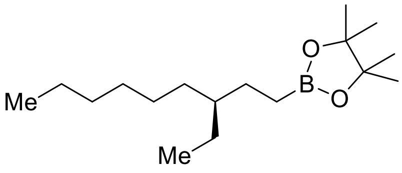

2i

***(R)*-2-(3-ethylnonyl)-4,4,5,5-tetramethyl-1,3,2-dioxaborolane:** By following the general procedure, **2i** was obtained in 45% yield. ^{1}H NMR (500 MHz, $CDCl_3$) δ 1.39–1.32 (m, 4H), 1.29–1.24 (m, 28H), 1.69–1.06 (m, 3H), 0.89–0.81 (m, 8H), 0.72 (t, *J* = 8.5 Hz, 2H); ^{13}C NMR (125 MHz, $CDCl_3$) δ 82.8, 40.9, 32.7, 32.0, 29.8, 29.7, 27.1, 26.7, 25.37, 24.8, 22.7, 14.1, 10.9; IR (neat) 2988, 1740, 1374, 1232, 1055 cm^{-1}; HRMS (ESI) calcd for $[C_{17}H_{35}BO_2+H^+]$: 283.2808, found: 283.2810; 33% ee was measured by chiral HPLC on OJ-H column with the corresponding naphthoate derivative (*i*PrOH:hexane = 1:99, 0.5 mL/min); t_R = 12.92 min (major), t_R = 13.46 min (minor).

[rac]

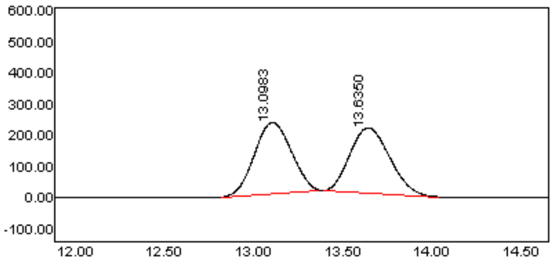

[chiral]

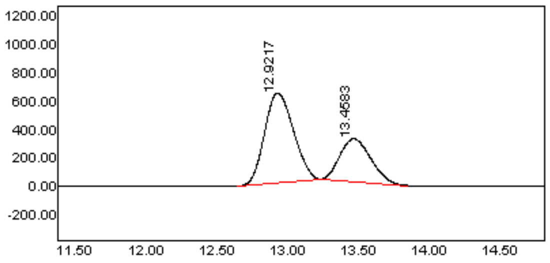

RT[분]	면적[mV*sec]	면적비[%]
13.0983	3283.5785	50.30
13.6350	3245.0188	49.70

RT[분]	면적[mV*sec]	면적비[%]
12.9217	8923.7172	66.50
13.4583	4494.8379	33.50

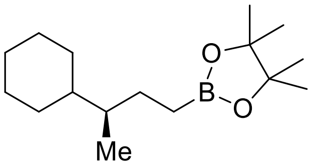

2j

***(R)*-2-(3-cyclohexylbutyl)-4,4,5,5-tetramethyl-1,3,2-dioxaborolane:** By following the general procedure, **2j** was obtained in 84% yield. ^{1}H NMR (500 MHz, $CDCl_3$) δ 1.72–7.70 (m, 2H), 1.64–1.56 (m, 3H), 1.51–1.45 (m, 1H), 1.40–1.33 (m, 1H), 1.24 (s, 12H), 1.22–1.16 (m, 4H), 1.34–0.96 (m, 3H), 0.94–0.82 (m, 1H), 0.80–0.79 (m, 3H), 0.71–0.65 (m, 1H); ^{13}C NMR (125 MHz, $CDCl_3$) δ 82.8, 42.3, 40.3, 30.9, 28.5, 28.1, 27.0, 26.9, 26.8, 24.9, 24.8, 15.6, 9.3 (C–B); IR (neat) 2975, 1732, 1368, 1235, 1052 cm^{-1}; HRMS (ESI) calcd for [$C_{16}H_{31}BO_2$+H^+]: 267.2495, found: 267.2495; 99% ee was measured by chiral HPLC on OD-H column with the corresponding naphthoate derivative (*i*PrOH:hexane = 1:99, 1.0 mL/min); t_R = 10.08 min (minor), t_R = 11.17 min (major).

[rac]

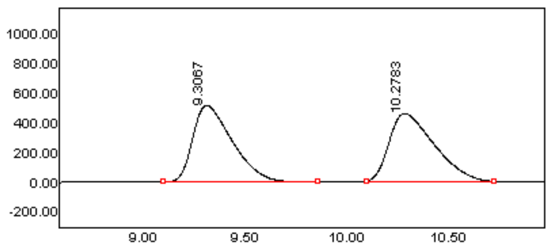

[chiral]

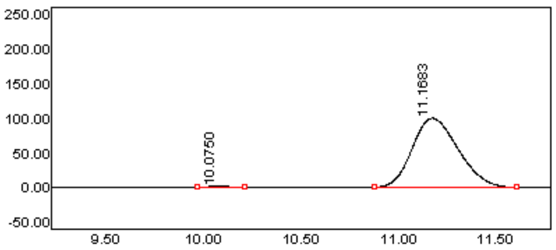

RT[분]	면적[mV*sec]	면적비[%]
9.3067	6990.1125	49.85
10.2783	7031.3109	50.15

RT[분]	면적[mV*sec]	면적비[%]
10.0750	8.8713	0.54
11.1683	1636.0305	99.46

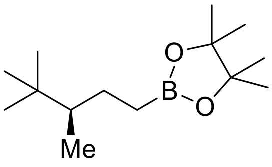

2k

***(R)*-4,4,5,5-tetramethyl-2-(3,4,4-trimethylpentyl)-1,3,2-dioxaborolane:** By following the general procedure, **2k** was obtained in 62% yield. ^{1}H NMR (500 MHz, $CDCl_3$) δ 1.69–1.64 (m, 1H), 1.25 (s, 12H), 1.05–0.91 (m, 3H), 0.84 (s, 9H), 0.81 (d, *J* = 7.0 Hz, 3H), 0.66–0.59 (m, 1H); ^{13}C NMR (125 MHz, $CDCl_3$) δ 82.8, 45.6, 33.2, 27.4, 25.6, 24.9, 24.8, 13.6; IR (neat) 2980, 1735, 1377, 1230, 1045 cm^{-1}; HRMS (ESI) calcd for [$C_{14}H_{29}BO_2$+H^+]: 241.2338, found: 241.2335; 99% ee was measured by chiral HPLC on OD-H column with the corresponding naphthoate derivative (*i*PrOH:hexane = 1:99, 1.0 mL/min); t_R = 16.35 min (minor), t_R = 17.64 min (major).

[rac] [chiral]

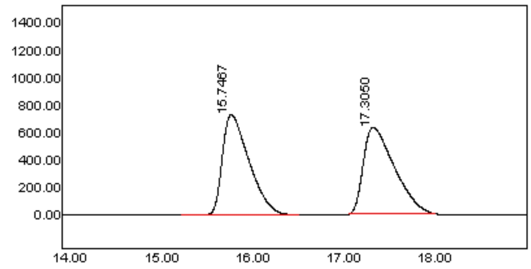

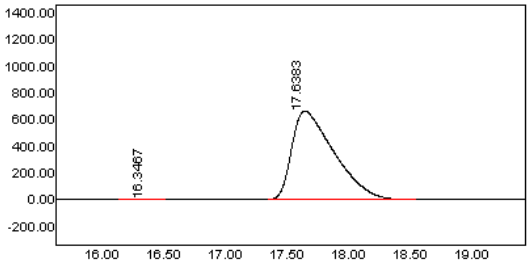

RT[분]	면적[mV*sec]	면적비[%]
15.7467	14816.9547	49.92
17.3050	14867.2969	50.08

RT[분]	면적[mV*sec]	면적비[%]
16.3467	80.3266	0.50
17.6383	15830.9781	99.50

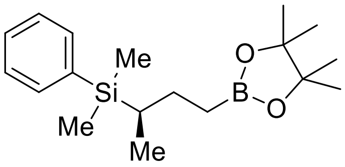

2l

***(R)*-dimethyl(phenyl)(4-(4,4,5,5-tetramethyl-1,3,2-dioxaborolan-2-yl)butan-2-yl)silane:** By following the general procedure, **2l** was obtained in 74% yield. ^{1}H NMR (500 MHz, $CDCl_3$) δ 7.51–7.47 (m, 2H), 7.34–7.32 (m, 3H), 1.70–1.64 (m, 1H), 1.23 (s, 12H), 1.21–1.17 (m, 1H), 0.95–0.90 (m, 4H), 0.82–0.77 (m, 1H), 0.73–0.67 (m, 1H), 0.25 (s, 3H), 0.24 (s, 3H); ^{13}C NMR (125 MHz, $CDCl_3$) δ 139.0, 133.9, 128.6, 127.6, 82.8, 25.8, 24.9, 24.8, 21.7, 13.6, 4.6, 4.9; IR (neat) 2978, 2955, 1377, 1315, 1112 cm^{-1}; HRMS (ESI) calcd for [$C_{18}H_{31}BO_2Si+H^+$]: 318.2264, found: 318.2265; >99% ee was measured by chiral HPLC on AD-H column with the corresponding alcohol (*i*PrOH:hexane = 1:99, 0.5 mL/min); t_R = 18.48 min (major), t_R = 19.38 min (minor).

[rac] [chiral]

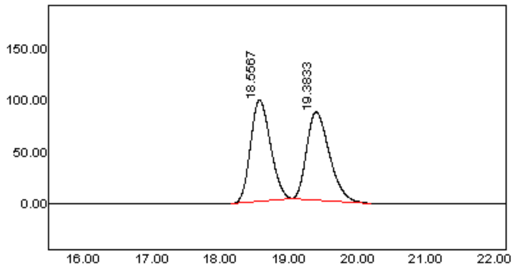

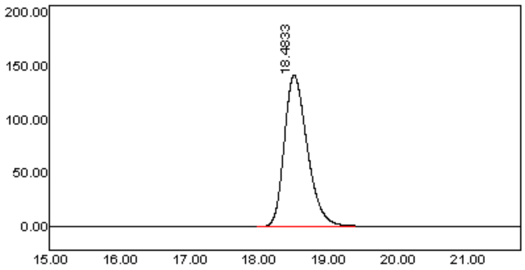

RT[분]	면적[mV*sec]	면적비[%]
18.5567	1976.0721	50.23
19.3833	1958.0600	49.77

RT[분]	면적[mV*sec]	면적비[%]
18.4833	3241.0523	100.00

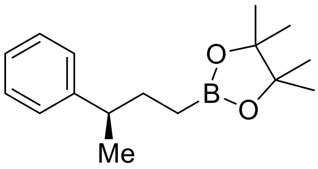

2m

***(R)*-4,4,5,5-tetramethyl-2-(3-phenylbutyl)-1,3,2-dioxaborolane:** By following the general procedure, **2m** was obtained in 79% yield. ^{1}H NMR (500 MHz, $CDCl_3$) δ 7.28–7.25 (m, 2H), 7.18–7.14 (m, 3H), 2.65–2.58 (m, 1H), 1.69–1.64 (m, 2H), 1.24 (d, *J* = 7.0 Hz, 3H), 1.22 (s, 12H),

0.75–0.62 (m, 2H); ^{13}C NMR (125 MHz, $CDCl_3$) δ 147.57, 128.2, 127.2, 125.7, 82.9, 42.2, 32.7, 24.8, 21.6; IR (neat) 1735, 1368, 1235, 1155 cm^{-1}; HRMS (ESI) calcd for [$C_{16}H_{25}BO_2+H^+$]: 261.2025, found: 261.2022; 99% ee was measured by chiral HPLC on AD-H column with the corresponding alcohol (*i*PrOH:hexane = 1:99, 1.0 mL/min); t_R = 7.98 min (minor), t_R = 9.62 min (major).

[rac]

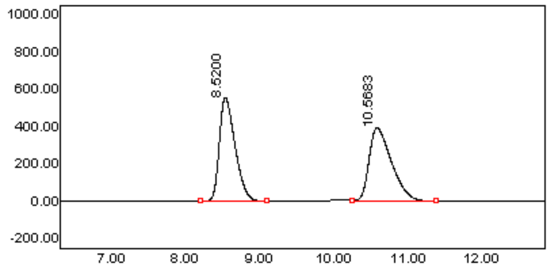

RT[분]	면적[mV*sec]	면적비[%]
8,5200	8196,3641	50,07
10,5683	8171,8422	49,93

[chiral]

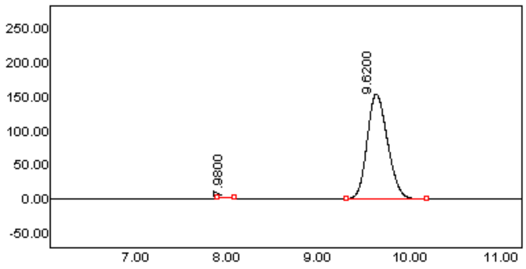

RT[분]	면적[mV*sec]	면적비[%]
7,9800	10,4106	0,44
9,6200	2372,9852	99,56

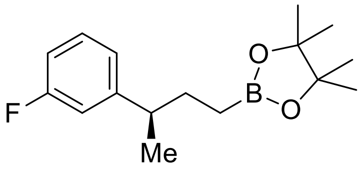

2n

***(R)*-2-(3-(3-fluorophenyl)butyl)-4,4,5,5-tetramethyl-1,3,2-dioxaborolane:** By following the general procedure, **2n** was obtained in 78% yield. ^{1}H NMR (500 MHz, $CDCl_3$) δ 7.23–7.19 (m, 1H), 6.94 (d, *J* = 7.5 Hz, 1H), 6.88–6.82 (m, 2H), 2.66–2.59 (m, 1H), 1.68–1.63 (m, 2H), 1.24–1.22 (m, 15H), 0.74–0.62 (m, 2H); ^{13}C NMR (125 MHz, $CDCl_3$) δ 163.0 (d, *J* = 242.5 Hz), 150.3 (d, *J* = 7.5 Hz), 129.5 (d, *J* = 7.5 Hz), 122.9 (d, *J* = 2.5 Hz), 113.8 (d, *J* = 21.3 Hz), 112.5 (d, *J* = 21.3 Hz), 83.0, 42.0, 41.9, 32.5, 24.8, 21.5; 19F NMR (470 MHz, $CDCl_3$) δ -114.0; IR (neat) 2978, 2925, 1360, 1325, 1142 cm^{-1}; HRMS (ESI) calcd for [$C_{16}H_{24}BFO_2+H^+$]: 279.1931, found: 279.1932; >99% ee was measured by chiral HPLC on AD-H column with the corresponding alcohol (*i*PrOH:hexane = 1:99, 1.0 mL/min); t_R = 11.70 min (major), t_R = 12.81 min (minor).

[rac]

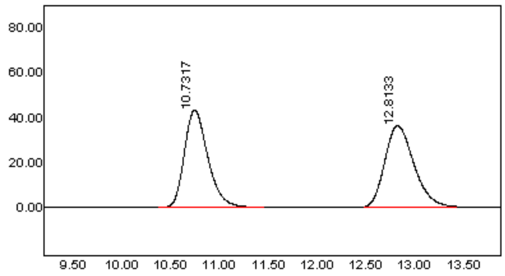

RT[분]	면적[mV*sec]	면적비[%]
10,7317	724,4968	49,55
12,8133	737,7582	50,45

[chiral]

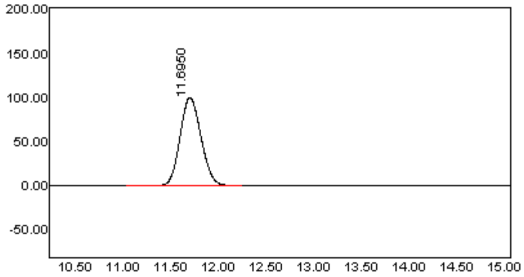

RT[분]	면적[mV*sec]	면적비[%]
11,6950	1523,7111	100,00

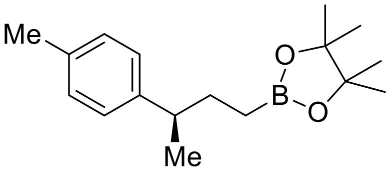

2o

***(R)*-4,4,5,5-tetramethyl-2-(3-(p-tolyl)butyl)-1,3,2-dioxaborolane:** By following the general procedure, **27n** was obtained in 71% yield. ^{1}H NMR (500 MHz, $CDCl_3$) δ 7.09–7.05 (m, 4H), 2.61–2.54 (m, 1H), 2.30 (s, 3H), 1.67–1.62 (m, 2H), 1.22–1.21 (m, 15H), 0.73–0.64 (m, 2H); ^{13}C NMR (125 MHz, $CDCl_3$) δ 144.5, 135.1, 128.9, 127.0, 82.9, 41.7, 32.8, 24.9, 24.8, 21.7, 21.0; IR (neat) 2975, 2927, 1360, 1322, 1144 cm^{-1}; HRMS (ESI) calcd for [$C_{17}H_{27}BO_2+H^+$]: 275.2182, found: 275.2185; >99% ee was measured by chiral HPLC on AS-H column with the corresponding alcohol (*i*PrOH:hexane = 1:99, 0.5 mL/min); t_R = 27.66 min (minor), t_R = 30.39 min (major).

[rac]

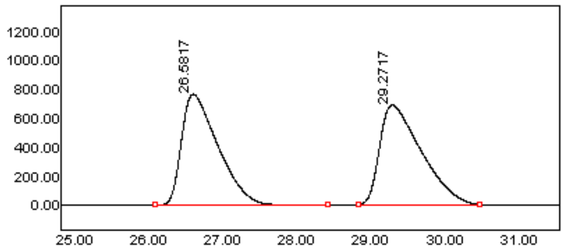

RT[분]	면적[mV*sec]	면적비[%]
26.5817	26396.2687	49.74
29.2717	26673.7875	50.26

[chiral]

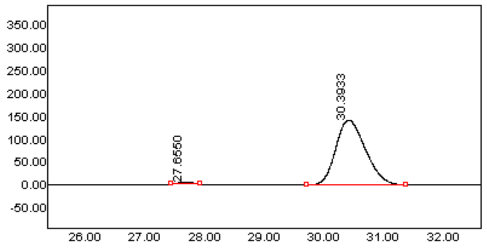

RT[분]	면적[mV*sec]	면적비[%]
27.6550	11.4514	0.23
30.3933	4954.1793	99.77

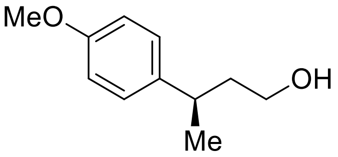

27p

***(R)*-2-(3-(4-methoxyphenyl)butyl)-4,4,5,5-tetramethyl-1,3,2-dioxaborolane:** By following the general procedure, **27o** was obtained in 59% yield. The characterization data for **2q** was concordant with that previously reported in the literature.[3] ^{1}H NMR (500 MHz, $CDCl_3$) δ 7.13 (d, *J* = 8.5 Hz, 2H), 6.85 (d, *J* = 8.5 Hz, 2H), 3.79 (s, 3H), 3.61–3.51 (m, 2H), 2.88–2.81 (m, 1H), 1.87–1.78 (m, 2H), 1.25 (d, *J* = 7.0 Hz, 3H); ^{13}C NMR (125 MHz, $CDCl_3$) δ 127.8, 113.9, 61.3, 55.3, 41.2, 35.7, 22.6; >99% ee was measured by chiral HPLC on OD-H column with the corresponding alcohol (*i*PrOH:hexane = 1:99, 1.0 mL/min); t_R = 13.83 min (minor), t_R = 15.29 min (major).

[rac]

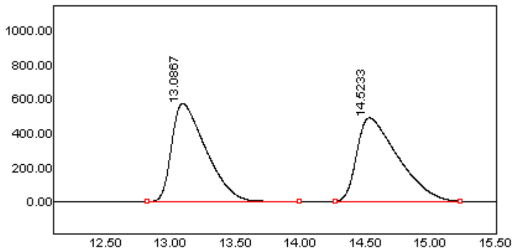

[chiral]

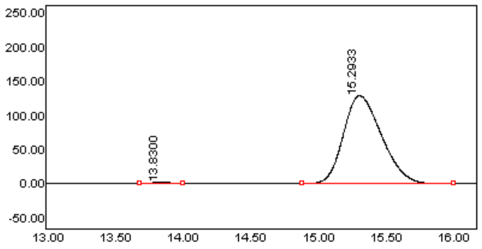

RT[분]	면적[mV*sec]	면적비[%]
13,0867	10495,8531	49,65
14,5233	10641,9281	50,35

RT[분]	면적[mV*sec]	면적비[%]
13,8300	6,3536	0,25
15,2933	2529,4346	99,75

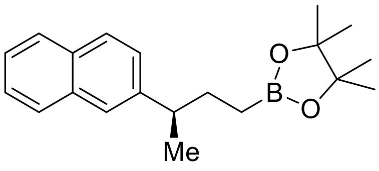

2q

***(R)*-4,4,5,5-tetramethyl-2-(3-(naphthalen-2-yl)butyl)-1,3,2-dioxaborolane:** By following the general procedure, **2q** was obtained in 75% yield. The characterization data for **2q** was concordant with that previously reported in the literature.[2] ^{1}H NMR (500 MHz, $CDCl_3$) δ 7.79–7.75 (m, 3H), 7.59 (s, 1H), 7.42–7.38 (m, 2H), 7.34 (dd, *J* = 8.5 Hz, 1.5 Hz, 1H), 2.83–2.76 (m, 1H), 1.80–1.72 (m, 2H), 1.33 (d, *J* = 7.0 Hz, 3H), 1.21 (s, 12H), 0.77–0.65 (m, 2H); ^{13}C NMR (125 MHz, $CDCl_3$) δ 145.0, 133.6, 132.2, 127.8, 127.6, 127.5, 126.0, 125.7, 125.4, 125.0, 82.9, 42.3, 32.6, 24.9, 24.8, 21.6, 9.6 (C–B); >99% ee was measured by chiral HPLC on OZ-H column with the corresponding alcohol (*i*PrOH:hexane = 1:99, 1.0 mL/min); t_R = 11.00 min (minor), t_R = 13.31 min (major).

[rac]

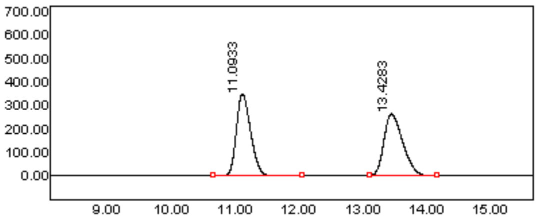

RT[분]	면적[mV*sec]	면적비[%]
11,0933	5415,9574	50,16
13,4283	5380,3441	49,84

[chiral]

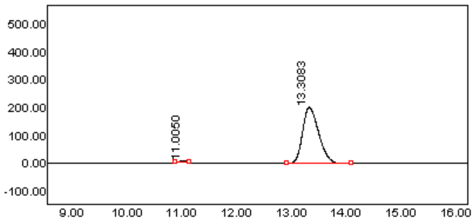

RT[분]	면적[mV*sec]	면적비[%]
11,0050	14,6631	0,35
13,3083	4121,6098	99,65

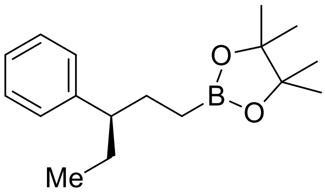

2r

***(R)*-4,4,5,5-tetramethyl-2-(3-phenylpentyl)-1,3,2-dioxaborolane:** By following the general procedure, **2r** was obtained in 44% yield. ^{1}H NMR (500 MHz, $CDCl_3$) δ 7.27–7.24 (m, 2H), 7.17–7.14 (m, 1H), 7.13–7.11 (m, 2H), 2.35–2.30 (m, 1H), 1.77–1.54 (m, 4H), 1.21 (s, 12H), 0.76 (t, *J* = 7.5 Hz, 3H), 0.67–0.57 (m, 2H); ^{13}C NMR (125 MHz, $CDCl_3$) δ 145.7, 128.1, 127.9, 125.7, 82.9, 50.3, 30.8, 29.1, 24.9, 24.8, 12.3; IR (neat) 3028, 2925, 1448, 1367, 1142 cm^{-1}; HRMS (ESI) calcd for $[C_{17}H_{27}BO_2+H^+]$: 275.2182, found: 275.2180; 74% ee was measured by chiral HPLC on OD-H column with the corresponding naphthoate derivative (*i*PrOH:hexane = 2:98, 1.0 mL/min); t_R = 12.32 min (major), t_R = 15.21 min (minor).

[rac]

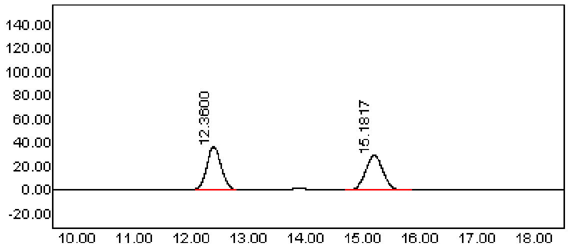

RT[분]	면적[mV*sec]	면적비[%]
12.3600	627.7141	50.20
15.1817	622.8269	49.80

[chiral]

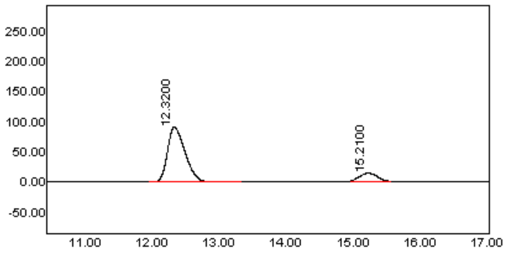

RT[분]	면적[mV*sec]	면적비[%]
12.3200	1655.8832	87.05
15.2100	246.3528	12.95

Detection of the Chiral Olefin Intermediate (Scheme 2)

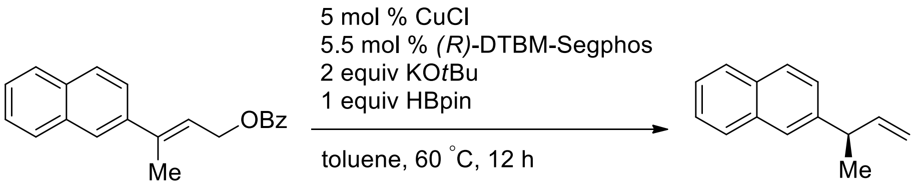

1q **1q'**

64% yield

A mixture of CuCl (5 mol %, 0.025 mmol), *(R)*-DTBM-Segphos (5.5 mol %, 0.0275 mmol) and KO*t*Bu (1 mmol) in anhydrous toluene (2 mL) were stirred for 5 min in a Schlenk tube under an atmosphere of nitrogen. Pinacolborane (0.5 mmol) was added to the reaction mixture and stirred for another 15 min at room temperature. Substrate **1q** dissolved in toluene (1 mL) was added. The reaction mixture was sealed, stirred at 60 °C and monitored by TLC. Upon completion of the reaction, the reaction mixture was diluted with diethyl ether (10 mL). After the aqueous layer was extracted with diethyl ether, the combined organic layers were dried over Na_2SO_4, and concentrated in vacuo. The product was purified by silica gel chromatography using hexanes/ethyl acetate as the eluent. The characterization data for **1q'** was concordant with that previously reported in the literature.[4] ^{1}H NMR (500 MHz, $CDCl_3$) δ 7.84–7.78 (m, 3H), 7.66 (s, 1H), 7.48–7.35 (m, 3H), 6.15–6.00 (m, 1H), 5.12–5.05 (m, 2H), 3.70–3.61 (m, 1H), 1.47 (d, *J* = 7.0 Hz, 3H); ^{13}C NMR (125 MHz, $CDCl_3$) δ 143.3, 135.2, 133.8, 132.5, 128.0, 127.8, 127.7, 126.5, 126.0, 125.4, 113.5, 43.4, 20.8.

Determination of Absolute Configuration of 3 (Scheme 3)

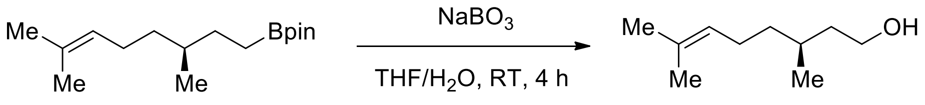

2a **3**

95% yield
99% ee

Sodium perborate (0.9 mmol) was added to **2**a (0.3 mmol) in THF (2 mL) and water (2 mL). The reaction mixture was stirred vigorously for 4 h at room temperature. The reaction was quenched with water and then, extracted with diethyl ether. The combined organic layers were dried over Na_2SO_4 and concentrated in vacuo. The product **3** was purified by silica gel chromatography (hexane/ethyl acetate) in 95% yield. The characterization data for **3** was concordant with that previously reported in the literature.[5] ^{1}H NMR (500 MHz, $CDCl_3$) δ 5.10 (t, J = 7.0 Hz, 1H), 3.73–3.64 (m, 2H), 2.05–1.92 (m, 2H), 1.68 (s, 3H), 1.61–1.57 (m, 5H), 1.41–1.33 (m, 2H), 1.22–1.15 (m, 2H), 0.91 (d, J = 6.5 Hz, 3H); ^{13}C NMR (125 MHz, $CDCl_3$) δ 131.3, 124.7, 61.2, 39.9, 37.2, 29.2, 25.7, 25.5, 19.5, 17.7; $[\alpha]_D$ = -3.5 (c = 1.2, $CHCl_3$) (lit.[5] $[\alpha]_D$ = -4.9 (c = 0.5, $CHCl_3$) for *(S)*-isomer with 99% ee); 99% ee were measured by chiral HPLC on AD-H column with the corresponding naphthoate derivative (*i*PrOH:hexane = 1:99, 1.0 mL/min); t_R = 14.75 min (minor), t_R = 15.72 min (major).

[rac]

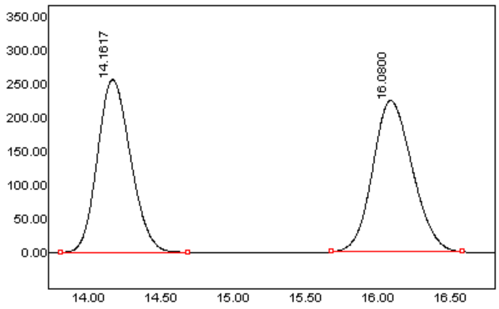

RT[분]	면적[mV*sec]	면적비[%]
14.1617	4082.9652	49.77
16.0800	4120.5590	50.23

[chiral]

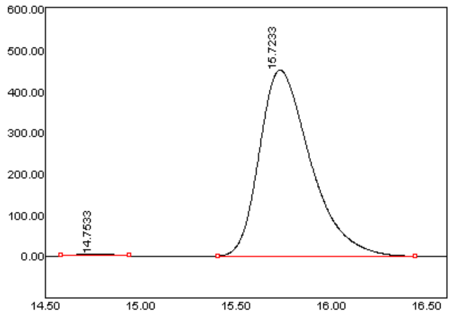

RT[분]	면적[mV*sec]	면적비[%]
14.7533	40.5508	0.48
15.7233	8491.1000	99.52

Suzuki-Miyaura Cross-Coupling Reaction of 2a (Scheme 3)

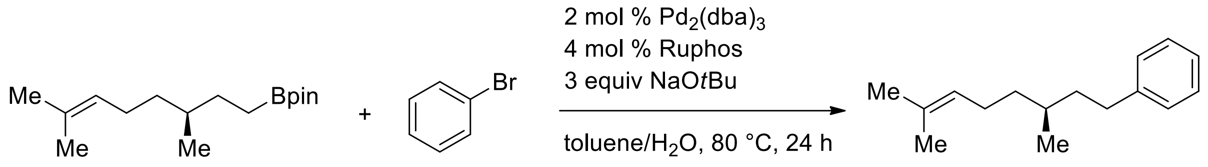

2a **4**

82% yield
99% ee

To a Schlenck tube, $Pd_2(dba)_3$ (2 mol%, 0.006 mmol), Ruphos (4 mol%, 0.012 mmol), **2a** (0.25 mmol), bromobenzene (0.3 mmol) and NaO*t*Bu (3 equiv, 0.9 mmol) were added. The mixture was dilu`ted with THF (0.5 mL) and H_2O (0.05 mL) under an atmosphere of nitrogen. The mixture was stirred for 24 h at 80 °C, and monitored by TLC. Upon completion of the reaction, the reaction mixture was diluted with diethyl ether (10 mL). After the aqueous layer was extracted with diethyl ether and the combined organic layers were dried over Na_2SO_4, and concentrated in vacuo. The product **4** was purified by silica gel chromatography (hexanes) in 82% yield. The characterization data for **4** was concordant with that previously reported in the literature.[6] ^{1}H NMR (500 MHz, $CDCl_3$) δ 7.28–7.25 (m, 2H), 7.18–7.15 (m, 3H), 5.12–5.08 (m, 1H), 2.68–2.62 (m, 1H), 2.59–2.53 (m, 1H), 2.03–1.92 (m, 2H), 1.68 (s, 3H), 1.66–1.62 (m, 1H), 1.60 (s, 3H), 1.47–1.36 (m, 3H), 1.20–1.16 (m, 1H), 0.94 (d, J = 6.5 Hz, 3H); ^{13}C NMR (125 MHz, $CDCl_3$) δ 143.2, 131.1, 128.4, 128.3, 125.5, 124.9, 38.9, 37.0, 33.5, 32.2, 25.7, 25.5, 19.6, 17.7; 99% ee was measured by chiral HPLC on AS-H column (*i*PrOH:hexane = 1:99, 0.5 mL/min); t_R = 23.60 min (minor), t_R = 28.71 min (major).

[rac]

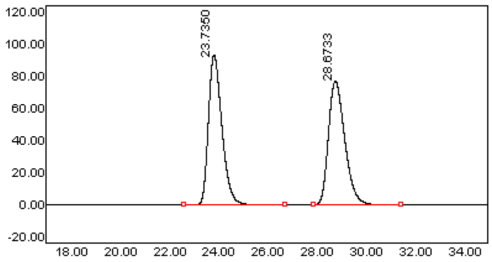

RT[분]	면적[mV*sec]	면적비[%]
23,7350	3556,9203	50,02
28,6733	3553,5957	49,98

[chiral]

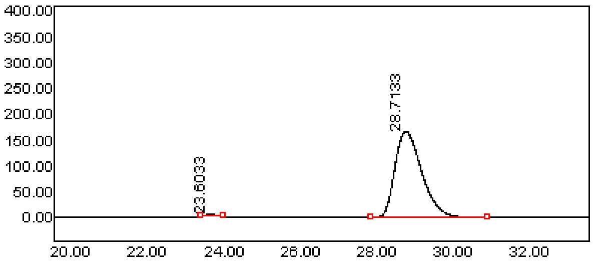

RT[분]	면적[mV*sec]	면적비[%]
23,6033	34,1650	0,42
28,7133	8179,5750	99,58

Amination of 2m (Scheme 3)

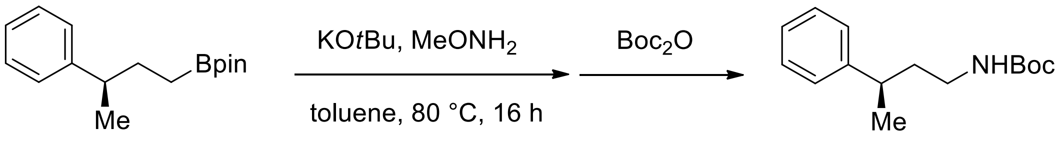

2m **5**

62% yield
99% ee

To a Schlenck tube, KO*t*Bu (1.5 equiv, 0.45 mmol), $MeONH_2$ (1.5 equiv, 0.45 mmol), **2m** (0.3 mmol), were added, and the mixture diluted with toluene (1.5 mL) under an atmosphere of nitrogen. The mixture was stirred for 16 h at 80 °C, and monitored by TLC. The reaction mixture was then cooled to room temperature and under an atmosphere of nitrogen, Boc_2O (1.5 equiv, 0.45 mmol) was added. The reaction mixture was stirred for 1 h at room temperature, and monitored by TLC. Upon completion of the reaction, the reaction mixture was diluted with ethyl acetate (10 mL). After the aqueous layer was extracted with ethyl acetate, the combined organic layers were dried over Na_2SO_4, and concentrated in vacuo. The product **5** was purified by silica gel chromatography in 62% yield. ^1H NMR (500 MHz, $CDCl_3$) δ 7.31–7.26 (m, 2H), 7.20–7.17 (m, 3H), 4.41 (brs, 1H), 3.06–2.97 (m, 2H), 2.78–2.71 (m, 1H), 1.81–1.73 (m, 2H), 1.43 (s, 9H), 1.26 (d, J = 7.0 Hz, 3H); ^{13}C NMR (125 MHz, $CDCl_3$) δ 146.6, 128.5, 126.9, 126.2, 39.1, 38.3, 37.7, 29.7, 28.4, 22.3, 14.1; >99% ee was measured by chiral HPLC on AS-H column (*i*PrOH:hexane = 1:99, 0.5 mL/min); t_R = 15.59 min (major), t_R = 17.23 min (minor).

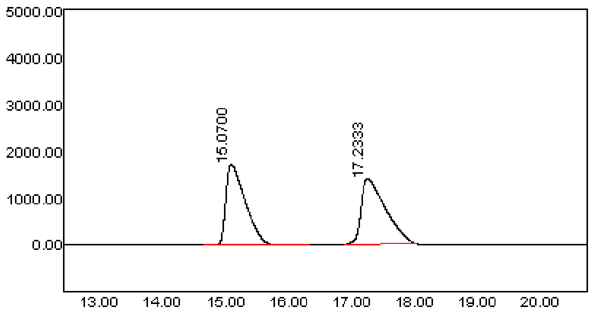

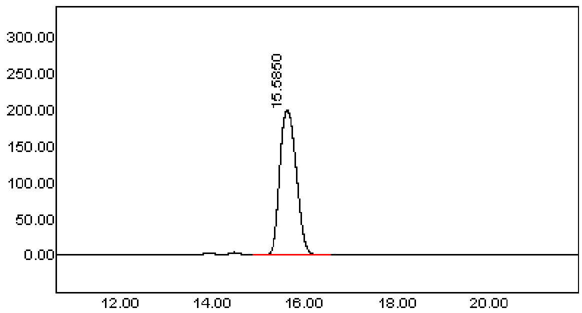

RT[분]	면적[mV*sec]	면적비[%]
15.0700	37414.2250	49.69
17.2333	37880.5781	50.31

RT[분]	면적[mV*sec]	면적비[%]
15.5850	4942.7570	100.00

Hydrolysis of Allylic Acetate

The reaction of **1m**-OAc under the standard reaction conditions afforded the corresponding allylic alcohol product by hydrolysis. Proposed mechanism of hydrolysis of **1m**-OAc to generate an allylic alcohol is shown in Scheme S1-b. Moreover, no hydrolysis occurred absence of KO*t*Bu base (Scheme S1-c). This result indicates that KO*t*Bu base leads to hydrolysis of the starting allyic acetate.

a) Reaction of **1m**-OAc under the standard reaction conditions

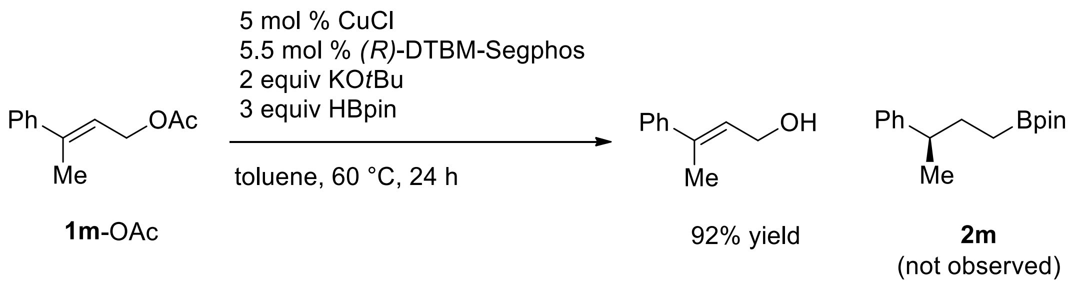

b) Proposed mechanism of hydrolysis

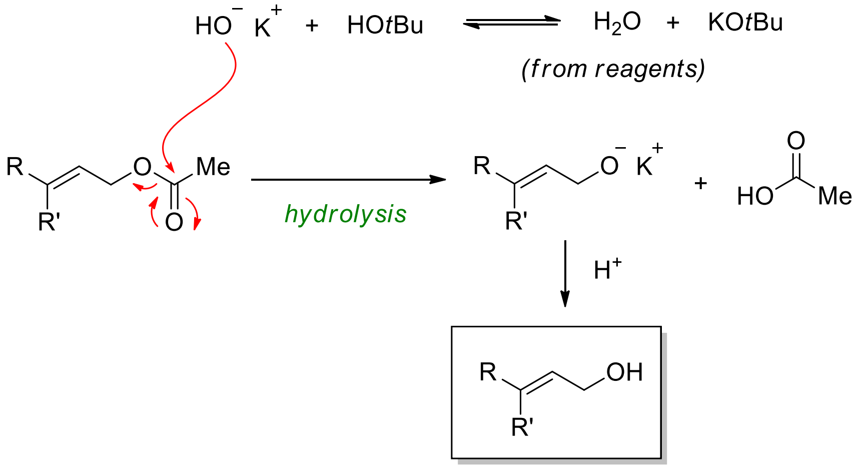

c) Reaction without KO*t*Bu base

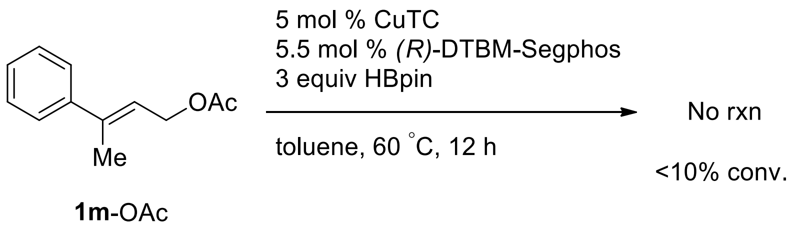

CuTC = copper thiophene-2-carboxylate

Scheme S1. Hydrolysis of allylic acetate

Reaction of *α*- or *β*- substituted allylic benzoate

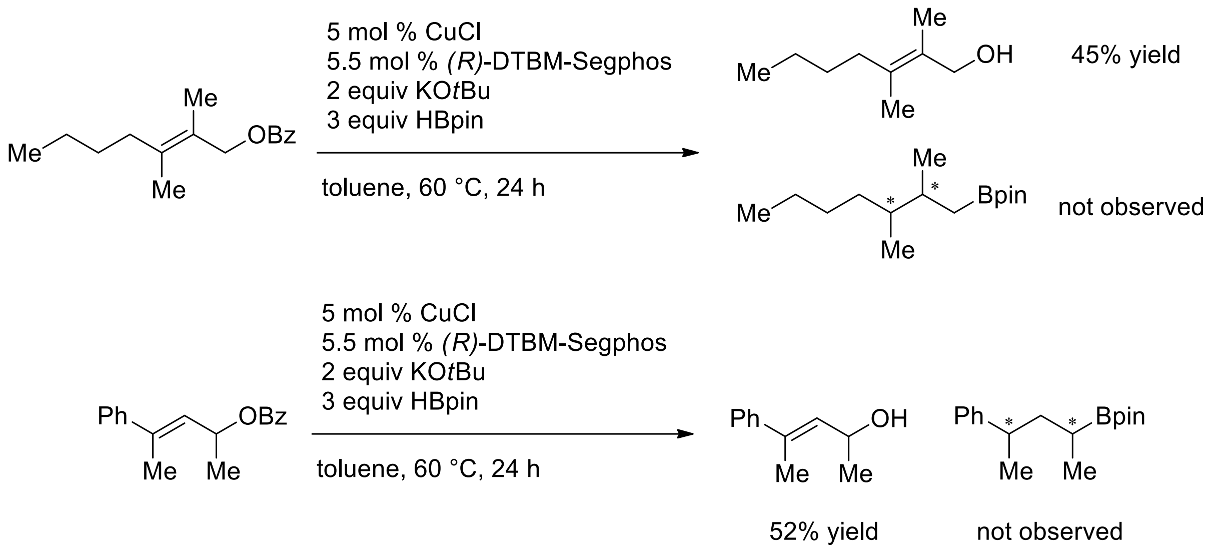

Scheme S2. Reaction of *α*- or *β*-substituted allylic benzoate

Computational Details

To gain an insight into the origin of high enantioselectivity, we performed DFT calculations at the M06-2X/6-13G* level using a suite of Gaussian 09 programs.[7] Images of the 3D structures were rendered using CYLView.[8]

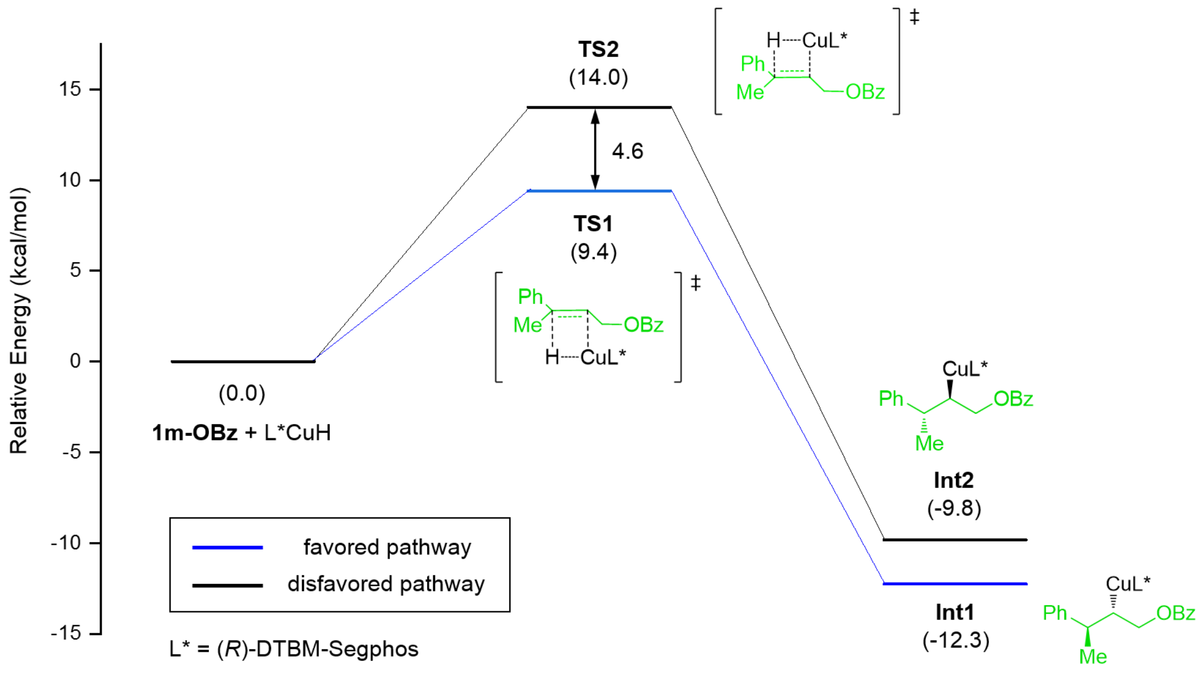

Figure S1. Computed energy profiles of hydrocupration of 1m-OBz.

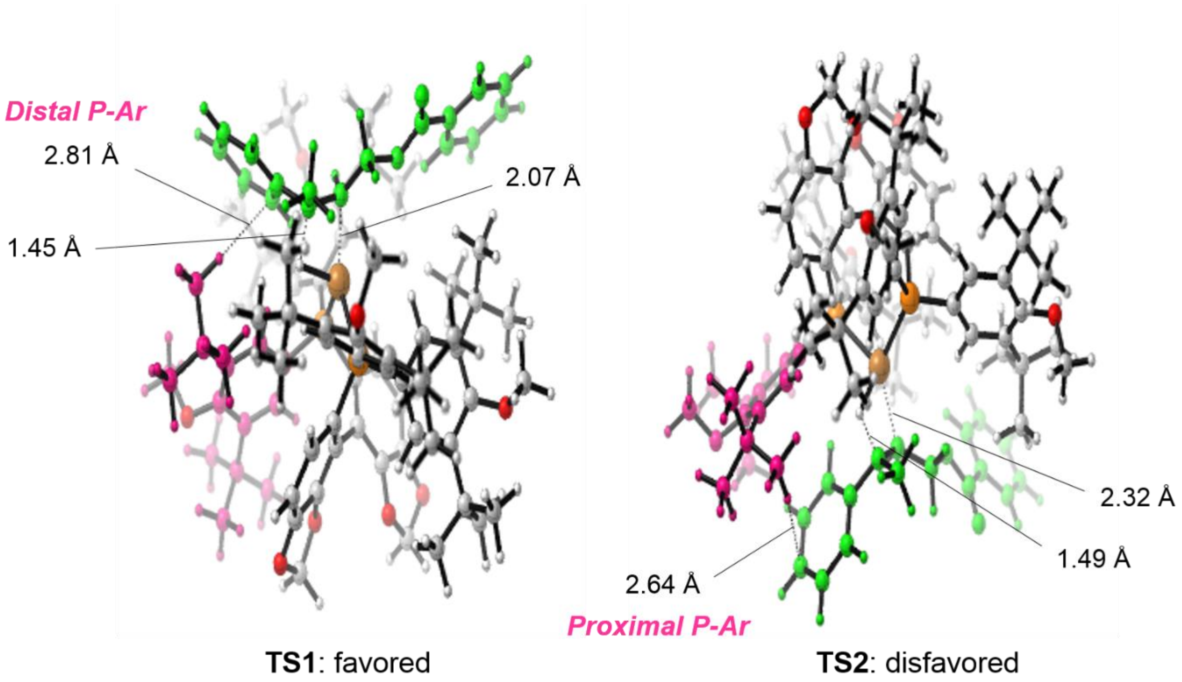

Figure S2. Transition state structures of the hydrocupration step.

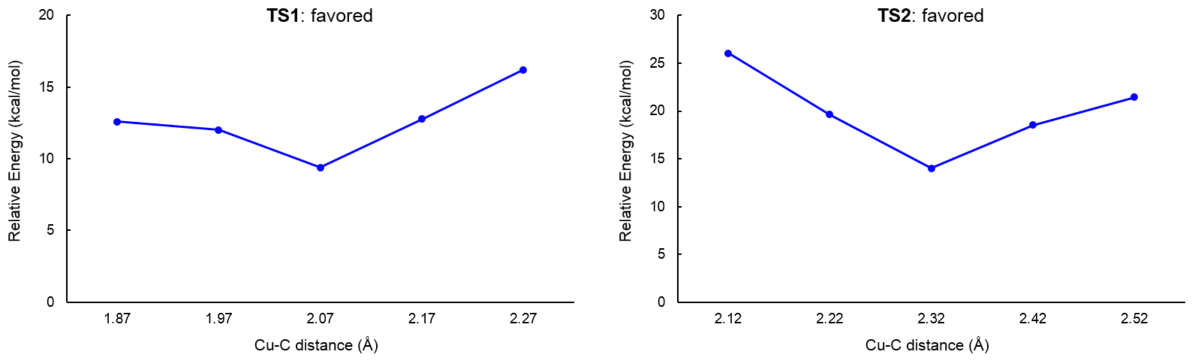

Figure S3. Evaluation of energy barriers for transition states

Cartesian Coordinates and Calculated Thermodynamic Properties of Selected Molecules

1m-OBz

Single-Point Energy: -807.531647

Thermal Correction to Enthalpy: 0.308139

Thermal Correction to Free Energy: 0.242593

C	2.30652900	0.92205200	0.22760500
C	1.14782200	0.61007100	-0.36914300
H	1.09610100	-0.28734700	-0.98283400
C	3.48459300	0.02409300	0.07630500
C	4.77907200	0.55385200	0.01195200
C	3.33373300	-1.36656700	0.00591400
C	5.88419800	-0.27592000	-0.14766500
H	4.92478200	1.62854000	0.07103500
C	4.43731000	-2.19745200	-0.14913200
H	2.34134300	-1.79744300	0.10167300
C	5.71783200	-1.65511200	-0.23042600
H	6.87801400	0.15739600	-0.20814300
H	4.29788600	-3.27341100	-0.19399000
H	6.58008400	-2.30423300	-0.34801100
C	-0.10970500	1.41567600	-0.35433000
H	-0.13960000	2.16774800	0.43806300
H	-0.26444200	1.93983000	-1.30487800
C	2.52415800	2.18064900	1.03226200
H	3.14919600	1.97289700	1.90571800
H	3.03889500	2.94422900	0.43713800
H	1.58601900	2.61757200	1.37822300
O	-1.20196400	0.50145200	-0.16633900
C	-2.42273900	1.05503000	-0.25398100
O	-2.60102900	2.23261900	-0.46207800
C	-3.51832800	0.06101900	-0.06388400
C	-4.83164400	0.52745900	-0.14287500

C	-3.26341300	-1.28852600	0.18546200
C	-5.89087500	-0.35586800	0.02632100
H	-4.99691100	1.58236100	-0.33732300
C	-4.32674100	-2.16882200	0.35469700
H	-2.23784600	-1.63675600	0.24559000
C	-5.63814700	-1.70399400	0.27478500
H	-6.91312000	0.00458600	-0.03447900
H	-4.13302500	-3.21921100	0.54906400
H	-6.46536300	-2.39517600	0.40729300

DTBM-Segphos-CuH

Single-Point Energy: -5803.764000

Thermal Correction to Enthalpy: 1.699458

Thermal Correction to Free Energy: 1.472397

C	-0.93095800	-2.57340100	3.36263400
C	-0.24043000	-1.38372800	3.17713600
C	-0.41857900	-0.55813800	2.08477900
C	-1.38767500	-0.98560900	1.13629200
C	0.35654500	0.71465100	2.07455500
C	0.12344500	1.58927900	3.11720200
C	0.80257000	2.78876100	3.28148400
C	1.76688900	3.19219300	2.38992700
C	2.03777100	2.31786700	1.32727100
C	1.37285000	1.10066400	1.15868200
O	-0.52059000	-3.16207500	4.52905000
C	0.23887200	-2.16025400	5.19500600
O	-0.80112300	1.45655500	4.12250200
C	-0.43707900	2.44318500	5.07861200
O	0.33528900	3.42657700	4.40042400
O	0.63631700	-1.20704800	4.21802600
P	-1.64723100	-0.04639500	-0.41723600
P	1.70558900	0.09986700	-0.33805000

H	2.29547300	4.13103300	2.50860700
H	2.80326000	2.60206100	0.61221800
H	1.12614800	-2.61371100	5.64024800
H	-0.39147200	-1.66756600	5.94783500
H	0.17519200	1.97886900	5.86395400
H	-1.33889700	2.90512000	5.48347600
C	3.35137100	0.64613000	-0.89082100
C	4.51506900	0.56855800	-0.12063600
C	3.40977200	1.21881500	-2.15170400
C	5.74534600	0.97492100	-0.62518700
H	4.44434200	0.17993900	0.88878800
C	4.60846600	1.68859100	-2.70751900
H	2.48720200	1.27547700	-2.72484700
C	5.77380700	1.46316600	-1.95825700
C	1.90964600	-1.61283500	0.24207100
C	1.28597800	-2.56955200	-0.54453000
C	2.60400500	-2.01904800	1.38350700
C	1.41103800	-3.94181000	-0.30732700
H	0.67558900	-2.22370100	-1.37575500
C	2.80318900	-3.37031200	1.65793400
H	2.97502200	-1.26113600	2.06627500
C	2.27598900	-4.31043900	0.73476100
C	-3.25485900	-0.65884600	-1.01486600
C	-4.44040100	-0.54545300	-0.30026500
C	-3.24709400	-1.37064400	-2.21321100
C	-5.64378500	-1.08191400	-0.76603400
H	-4.42338100	-0.03797100	0.65967200
C	-4.41540100	-1.90961900	-2.75163300
H	-2.30164800	-1.46799100	-2.73771400
C	-5.61569800	-1.67805700	-2.04421300
C	-1.91813300	1.67795500	0.10003100
C	-1.28460700	2.62827700	-0.68587400
C	-2.67037300	2.09860700	1.19919400
C	-1.45398200	4.00276500	-0.49354600
H	-0.63001000	2.27688000	-1.48015000

C	-2.91887700	3.45109000	1.42267700
H	-3.04528000	1.35104800	1.89101800
C	-2.37438700	4.37840600	0.49675300
C	-2.06658100	-2.19123000	1.33082700
C	-1.85151000	-3.01606200	2.44415100
H	-2.38948900	-3.94724100	2.57975900
H	-2.79948800	-2.50703200	0.59574800
C	0.56133500	-4.87663200	-1.19289700
C	0.53637100	-6.35392300	-0.76753000
H	0.29513900	-6.46230000	0.29291100
H	-0.24031300	-6.86152700	-1.35034500
H	1.47519600	-6.87661400	-0.95698000
C	1.02545300	-4.77196900	-2.65592300
H	0.94956100	-3.74438000	-3.02834800
H	2.06568300	-5.09509900	-2.77149900
H	0.39992200	-5.40918600	-3.29146600
C	-0.90474600	-4.39736400	-1.10957900
H	-1.28021600	-4.49738500	-0.08619200
H	-1.03252900	-3.35195700	-1.41176600
H	-1.52833400	-5.01242400	-1.76893800
C	3.49875600	-3.82161700	2.95457400
C	4.81144400	-4.57770800	2.68621400
H	5.34580000	-4.72561100	3.63183200
H	4.62639400	-5.56141900	2.25333200
H	5.46565400	-4.00742100	2.01657900
C	3.83782400	-2.61839400	3.84568600
H	4.57942600	-1.95933800	3.37951700
H	2.94878400	-2.02306900	4.07880700
H	4.26562900	-2.98221700	4.78570800
C	2.53966000	-4.73221700	3.74225200
H	2.31315100	-5.64284600	3.18435900
H	2.99651100	-5.01667500	4.69807000
H	1.59585300	-4.21617400	3.94751300
C	6.99752700	0.98826700	0.26954400
C	4.49824100	2.42836700	-4.05747600

C	5.74973600	3.21477800	-4.48184000
H	5.48241900	3.84021100	-5.34058000
H	6.10344800	3.86772900	-3.67940800
H	6.58034300	2.57960900	-4.79143600
C	3.36844100	3.47583100	-3.93029700
H	2.39014700	3.02336000	-3.74584700
H	3.58335900	4.17705800	-3.11660800
H	3.29462200	4.04458900	-4.86337200
C	4.10609400	1.43656100	-5.16647100
H	4.87455300	0.67135500	-5.31703500
H	3.16582900	0.92734200	-4.92950800
H	3.97360700	1.97118800	-6.11407900
C	7.49692900	2.43901600	0.39617700
H	8.38519500	2.47229200	1.03790900
H	7.75988900	2.85032700	-0.58083100
H	6.72599400	3.07255300	0.84802400
C	6.67442300	0.48081700	1.68277200
H	5.89618200	1.08017900	2.16670300
H	6.35279200	-0.56732000	1.67446200
H	7.57584600	0.54721600	2.29991300
C	8.13447900	0.10674800	-0.27628800
H	7.77095700	-0.89523000	-0.53063700
H	8.60220600	0.55165600	-1.15475400
H	8.90894400	-0.00361300	0.49118400
C	-3.67957200	3.92039500	2.67518100
C	-0.57849400	4.93174700	-1.35920900
C	-0.94338800	4.75934500	-2.84356900
H	-1.98251900	5.04746300	-3.03675900
H	-0.29542200	5.39093500	-3.46200200
H	-0.81536600	3.72183700	-3.17113300
C	0.89152100	4.49901300	-1.16183100
H	1.19193700	4.64197800	-0.11865300
H	1.06909900	3.44924700	-1.42133300
H	1.53991200	5.11205700	-1.79758700
C	-0.62163200	6.42416500	-0.99337100

H	-1.56031100	6.91011100	-1.26414700
H	-0.45075300	6.58244800	0.07415500
H	0.17560200	6.93115700	-1.54816800
C	-2.76847800	4.86434200	3.48031000
H	-2.53588900	5.76668100	2.91148500
H	-3.26691900	5.16095900	4.41129700
H	-1.82430900	4.36979600	3.73146800
C	-4.99041300	4.64960800	2.33290800
H	-5.56803200	4.80867200	3.25090700
H	-4.80127600	5.62660700	1.88695400
H	-5.60618100	4.05510000	1.64806500
C	-4.03949200	2.73245000	3.57829600
H	-4.74747700	2.05005800	3.09252700
H	-3.15227200	2.15940000	3.86716000
H	-4.51761700	3.10937800	4.48833600
C	-6.84945900	-1.02389400	0.19772500
C	-4.35804300	-2.79675900	-4.00721900
C	-2.91552900	-2.93977500	-4.51669500
H	-2.49129600	-1.98208000	-4.83758800
H	-2.25214300	-3.36765400	-3.75681600
H	-2.91065600	-3.61485100	-5.37840800
C	-4.86628600	-4.20119700	-3.63341500
H	-5.89785700	-4.16565800	-3.27543600
H	-4.82579500	-4.85734900	-4.51072900
H	-4.23752000	-4.64017500	-2.85072500
C	-5.20081200	-2.24632900	-5.17086500
H	-6.26962100	-2.35079000	-4.97959000
H	-4.96865200	-1.19304300	-5.36315800
H	-4.97050100	-2.81020200	-6.08172400
C	-8.06578600	-1.87738700	-0.20199100
H	-8.76007500	-1.89427200	0.64543400
H	-8.61166400	-1.48072400	-1.05838000
H	-7.77751400	-2.90654600	-0.42958600
C	-6.38894800	-1.57850500	1.56492000
H	-6.03191300	-2.60873500	1.46162000

H	-5.58765800	-0.98870400	2.01761200
H	-7.23310600	-1.57677200	2.26270800
C	-7.29747300	0.43511000	0.38941100
H	-6.47459400	1.06156100	0.75135500
H	-7.65962900	0.87100200	-0.54745500
H	-8.11095500	0.48495700	1.12225600
O	-2.77730600	5.69505300	0.59459600
C	-3.74330700	6.02610600	-0.39302300
H	-3.97428500	7.08557300	-0.26234100
H	-3.35381500	5.85678600	-1.40275100
H	-4.65408900	5.42956600	-0.26587800
O	-6.79664300	-2.06143500	-2.64576100
O	7.01098200	1.72270900	-2.51179900
O	2.64220700	-5.63271400	0.88794300
C	7.42759700	0.69754000	-3.40286100
H	6.71750700	0.57378200	-4.22653000
H	8.39805100	1.00386600	-3.79946200
H	7.52690400	-0.26048000	-2.88066400
C	3.64523700	-6.01505200	-0.04251700
H	3.85689400	-7.07054700	0.14182500
H	3.30223900	-5.88435700	-1.07449000
H	4.55748700	-5.42419800	0.09925600
C	-7.48614100	-0.95941000	-3.21843800
H	-8.40699500	-1.35631900	-3.65108800
H	-7.73223500	-0.20691700	-2.46113100
H	-6.88427300	-0.48334700	-4.00013200
Cu	0.07465700	0.03437200	-1.97930200
H	0.11019600	0.04995900	-3.55286800

TS1 (Favored Transition State)

Single-Point Energy: -6611.274438

Thermal Correction to Enthalpy: 2.023527

Thermal Correction to Free Energy: 1.776289

C	4.27648000	1.08318900	-3.27246600
C	2.94759600	1.03554800	-2.87527000
C	1.92059600	0.72444100	-3.77863000
C	2.28135600	0.46717300	-5.10344900
C	3.61627400	0.52149700	-5.50991600
C	4.62097900	0.82704100	-4.59969800
H	5.04027000	1.30984400	-2.53216600
H	2.70537000	1.24744800	-1.83920900
H	1.52131400	0.22906200	-5.84052100
H	3.86490800	0.32024400	-6.54804200
H	5.65885100	0.86033600	-4.91746400
C	0.48351300	0.68826500	-3.30069000
H	0.54835000	-0.54185300	-2.53469000
C	0.07082400	1.89220200	-2.49912700
H	0.92621900	2.55719600	-2.35653100
C	-1.09308100	2.67911900	-3.03384400
H	-1.99684200	2.08108300	-3.18720600
H	-0.89111400	3.20426400	-3.98070600
O	-1.41409300	3.72191200	-2.07615100
C	-2.32276200	4.61151000	-2.49009700
O	-2.94083000	4.51872700	-3.52677500
C	-0.51793600	0.22498800	-4.34949300
H	-1.49522000	0.04216800	-3.89378100
H	-0.64099300	0.98705100	-5.12970200
H	-0.19404400	-0.70568000	-4.82573300
Cu	-0.01530800	0.39972200	-1.06271400
P	-1.54347200	-1.04709300	-0.15956100
P	1.61283300	0.21399100	0.57150200
C	-0.73952300	-2.48330500	0.67554900
C	-2.70707100	-1.80267000	-1.34864600
C	-2.62766800	-0.34276300	1.12184100
C	0.82930600	0.17257000	2.22944300
C	2.79710700	1.60711700	0.62196300
C	2.69928600	-1.24840400	0.49757200

C	-0.00155500	-2.26823400	1.87251700
C	-0.81014400	-3.76579500	0.12802700
C	-4.08040700	-1.78157600	-1.16425400
C	-2.18888400	-2.38237400	-2.51200600
C	-2.76744600	1.03648500	1.13697500
C	-3.32301000	-1.11388700	2.05953700
C	0.12212100	-0.99184400	2.63123200
C	0.87258400	1.28962100	3.06566200
C	4.16947000	1.41804500	0.79087200
C	2.32345300	2.88229700	0.33244300
C	2.84581700	-1.83173300	-0.75150700
C	3.41956800	-1.76370300	1.57949200
C	0.56855900	-3.38842000	2.44196000
C	-0.19266600	-4.88167200	0.71501200
H	-1.38945900	-3.92667600	-0.77429000
C	-4.97339500	-2.26751200	-2.12924600
H	-4.47551100	-1.36241700	-0.24594300
C	-3.01821700	-2.89024300	-3.50711400
H	-1.10919300	-2.42325200	-2.63136700
C	-3.63922000	1.68779700	2.02029900
H	-2.17772700	1.62211700	0.43447500
C	-4.24773900	-0.53361300	2.92390400
H	-3.13380100	-2.18219200	2.09555600
C	-0.42566900	-0.95714500	3.89726800
C	0.27248300	1.30781500	4.33419000
H	1.41489500	2.17159100	2.73912300
C	5.08234000	2.44946800	0.58352100
H	4.52672700	0.43219600	1.05594900
C	3.18365500	3.97571500	0.16853400
H	1.25206000	3.02187300	0.18954500
C	3.74024100	-2.88144000	-0.99156000
H	2.24063700	-1.45580000	-1.57219800
C	4.37103300	-2.76602500	1.39820000
H	3.23377000	-1.35749800	2.56929400
C	0.48389400	-4.66023700	1.89008300

O	1.30196000	-3.45227100	3.59951800
H	-0.27061100	-5.87069900	0.27875400
C	-4.41503000	-2.72664300	-3.33287600
C	-6.46738200	-2.28360500	-1.74050700
C	-2.43437000	-3.67017000	-4.69810400
C	-4.44901500	0.86567000	2.81828900
C	-3.57707900	3.22666600	2.05554700
C	-4.93840100	-1.35714500	4.02456300
C	-0.35495400	0.15096600	4.73246300
O	-1.11249400	-1.95639100	4.53986000
H	0.32531800	2.17760600	4.97878300
C	4.56342900	3.70383400	0.18791800
C	6.58020700	2.23308200	0.86334000
C	2.52389200	5.36113900	0.00999500
C	4.57008800	-3.24807900	0.07903300
C	3.66924000	-3.54193700	-2.38368200
C	5.10487600	-3.39107500	2.59696900
O	1.15262300	-5.54618300	2.69464600
C	1.33283400	-4.83751300	3.91448700
O	-5.22837800	-3.02695300	-4.40663400
C	-7.37984000	-3.10260200	-2.66915400
C	-6.59076700	-2.94274400	-0.34782000
C	-6.99872300	-0.84321600	-1.64061000
C	-0.91828200	-3.85868900	-4.54268700
C	-3.06632600	-5.07431600	-4.73127600
C	-2.67951100	-2.96508800	-6.04256500
O	-5.48508100	1.41281800	3.54716200
C	-4.13696000	3.80700300	0.74681000
C	-2.08724100	3.62171000	2.18019600
C	-4.28479700	3.88632900	3.25067600
C	-4.58201300	-0.73178100	5.38619600
C	-6.46850200	-1.40311300	3.87078800
C	-4.43631400	-2.80783000	4.02101300
O	-0.98038100	-0.14098100	5.91773400
C	-1.08521200	-1.55902000	5.90386000

O	5.46224200	4.67467900	-0.20188200
C	7.01307500	3.20248200	1.97825700
C	6.85115900	0.80147400	1.35220000
C	7.46450000	2.46533600	-0.37387700
C	3.47529400	6.56809200	0.08017000
C	1.54792800	5.54310900	1.19407500
C	1.73215600	5.41853400	-1.30765000
O	5.62360100	-4.11426900	-0.13199300
C	4.43311700	-4.86911300	-2.52075000
C	4.14271000	-2.55439800	-3.46337500
C	2.18564300	-3.87749800	-2.66289500
C	6.63063100	-3.20220800	2.52799500
C	4.63422100	-2.76817300	3.91890700
C	4.77691600	-4.89532500	2.63411300
H	2.30156500	-5.09067800	4.34562100
H	0.50202300	-5.07093800	4.59527900
C	-5.63973600	-1.85798400	-5.10206700
H	-8.35955100	-3.19775000	-2.18794000
H	-7.54007800	-2.63625500	-3.64174300
H	-6.98143700	-4.10662400	-2.83681900
H	-6.21016800	-3.96919200	-0.37132000
H	-6.04777600	-2.39741200	0.42951100
H	-7.64534800	-2.97436300	-0.05264400
H	-6.42820900	-0.25903100	-0.91021700
H	-6.94000500	-0.32222400	-2.60148900
H	-8.04819600	-0.85316500	-1.32430100
H	-0.37560200	-2.90753300	-4.57338300
H	-0.66532500	-4.36559900	-3.60441700
H	-0.54880700	-4.47752900	-5.36681700
H	-4.14848300	-5.02111600	-4.86206100
H	-2.64306300	-5.64979700	-5.56277500
H	-2.85103100	-5.61321700	-3.80203800
H	-3.73129400	-3.00395400	-6.32908000
H	-2.35684500	-1.91822700	-6.00300700
H	-2.10263800	-3.46588600	-6.82830300

C	-6.61963100	1.68287300	2.73716700
H	-5.18312900	3.52137300	0.59357900
H	-4.08180700	4.90180700	0.76410500
H	-3.56133300	3.45480700	-0.11609600
H	-1.65171600	3.18784800	3.08709400
H	-1.48982800	3.28753800	1.32489000
H	-2.00348200	4.71212800	2.23733800
H	-5.37303100	3.84090100	3.18963300
H	-3.97832000	3.43504300	4.19838700
H	-4.00419200	4.94549400	3.26495200
H	-5.04779600	0.24839900	5.50403000
H	-4.92483100	-1.38114800	6.20091300
H	-3.49892600	-0.59729600	5.47108400
H	-6.88253900	-2.12494800	4.58408300
H	-6.92584200	-0.43496400	4.07750000
H	-6.75624400	-1.72705200	2.86392100
H	-4.70746500	-3.32962300	3.09586000
H	-3.34997200	-2.86036400	4.14336600
H	-4.90317500	-3.34713100	4.85173400
H	-0.19725600	-1.99254100	6.38632700
H	-2.01034400	-1.86442100	6.39141700
C	5.53516400	4.79017100	-1.61737400
H	8.07582400	3.05875500	2.20564600
H	6.86092200	4.24056800	1.67372000
H	6.44014600	3.01718500	2.89307300
H	6.29065400	0.56605100	2.26367400
H	6.59633000	0.05345900	0.59124200
H	7.91697300	0.69866100	1.58075200
H	7.11104800	1.88133000	-1.23166600
H	7.49999700	3.51961200	-0.64970000
H	8.48874400	2.14416500	-0.15238200
H	2.86433100	7.47420400	0.15970400
H	4.12997700	6.51722600	0.95378500
H	4.09959200	6.67932300	-0.80729100
H	0.78684500	4.75889400	1.24607700

H	2.09488600	5.54328700	2.14341600
H	1.03375800	6.50560500	1.09420000
H	2.39284200	5.30362500	-2.17391500
H	0.97329100	4.63087100	-1.36727500
H	1.22105000	6.38476100	-1.39630000
C	6.73024200	-3.46685100	-0.74281600
H	4.17930700	-5.56366600	-1.71547000
H	4.14881500	-5.32842500	-3.47391600
H	5.51707100	-4.74633900	-2.53184100
H	3.52669600	-1.64879400	-3.47496500
H	5.18232200	-2.24574300	-3.30816400
H	4.07215400	-3.02205600	-4.45251000
H	1.78665500	-4.54846000	-1.89423300
H	1.55340300	-2.98380400	-2.69551000
H	2.10076000	-4.37426700	-3.63620200
H	7.07774500	-3.51319200	3.47929300
H	7.07909300	-3.80682400	1.73926200
H	6.89056800	-2.14935500	2.36481200
H	4.89306400	-1.70470200	3.98070800
H	3.55301500	-2.86731100	4.05430100
H	5.13425600	-3.27803600	4.74890000
H	5.20570000	-5.41183000	1.77301800
H	5.18349800	-5.34641000	3.54757000
H	3.69322700	-5.05370000	2.61449300
H	-6.22544200	-2.19321600	-5.96071500
H	-6.25700000	-1.21403300	-4.46681500
H	-4.77349800	-1.28158600	-5.44559900
H	-7.39861400	2.05857300	3.40429600
H	-6.39492700	2.43726800	1.97597600
H	-6.96891100	0.77137500	2.23692700
H	4.56284900	5.05345900	-2.04631100
H	6.25775500	5.58107100	-1.82970500
H	5.86911700	3.85009100	-2.07076600
H	7.50733400	-4.22415500	-0.86700300
H	6.46052200	-3.05467700	-1.72101900

H	7.10453700	-2.65284700	-0.11060700
C	-2.50620100	5.74140300	-1.52673300
C	-3.55048400	6.63721000	-1.75831900
C	-1.68562600	5.89755800	-0.41083300
C	-3.77901100	7.68129700	-0.86832200
H	-4.17255600	6.49171500	-2.63580100
C	-1.91023500	6.94767700	0.47375900
H	-0.89064800	5.18221000	-0.23378900
C	-2.95940400	7.83686500	0.24886900
H	-4.59545500	8.37499400	-1.04387500
H	-1.27411300	7.06415700	1.34692400
H	-3.13849000	8.65139600	0.94450000

TS2 (Disfavored Transition State)

Single-Point Energy: -6611.227501

Thermal Correction to Enthalpy: 2.023641

Thermal Correction to Free Energy: 1.776333

C	-0.43941100	0.86099700	-3.44409200
H	-0.60707600	-0.41653300	-2.69443300
C	-0.22839100	2.09900000	-2.65143600
C	0.92069800	2.95872800	-3.09427800
H	0.80049700	3.35474100	-4.11140500
H	1.89000700	2.44465300	-3.05910700
O	1.01320900	4.11582400	-2.22630900
C	1.49144100	5.23682000	-2.78472200
O	1.91133700	5.31586200	-3.91703600
Cu	-0.10023200	0.31406100	-1.17496500
P	-1.72378100	-0.48659100	0.25571000
P	1.78101100	-0.45192400	-0.08408700
C	-1.30776500	-2.10148200	1.03854300
C	-3.35479800	-0.77057500	-0.51265000
C	-2.08980300	0.61078700	1.66122600

C	1.48524200	-0.17559900	1.72158300
C	3.44333000	0.27965400	-0.33854300
C	2.06385100	-2.25213300	-0.23024000
C	-0.22455500	-2.14481900	1.95627100
C	-2.02873100	-3.26471300	0.76208100
C	-4.47688800	-0.03293300	-0.14181300
C	-3.43872300	-1.61638300	-1.61340300
C	-1.70380200	1.93458200	1.52124900
C	-2.74525200	0.20512900	2.82791500
C	0.56219400	-0.97878500	2.44537400
C	2.13058700	0.88296800	2.36670800
C	4.61306000	-0.46422100	-0.18796400
C	3.53919500	1.64723400	-0.58102800
C	1.53237800	-2.86606500	-1.35380200
C	2.78041000	-3.02651400	0.69062000
C	0.00785300	-3.36278100	2.56110200
C	-1.74633200	-4.49890400	1.36811100
H	-2.87050900	-3.22035200	0.08143700
C	-5.70125400	-0.19303500	-0.78987000
H	-4.38407900	0.67568100	0.67041800
C	-4.63241800	-1.82858600	-2.30951700
H	-2.53499300	-2.12226500	-1.94286100
C	-2.01921400	2.90759000	2.47839000
H	-1.13973700	2.21172500	0.63242800
C	-3.12236900	1.13067800	3.79721800
H	-2.95775600	-0.85028400	2.96626900
C	0.42329800	-0.68129200	3.78732000
C	1.94896500	1.17628700	3.72656900
H	2.82685400	1.49644000	1.80547600
C	5.87316400	0.11213800	-0.31580800
H	4.53181700	-1.52072700	0.02224000
C	4.77754900	2.30635900	-0.64659000
H	2.61727200	2.21855300	-0.68531000
C	1.74082900	-4.22108200	-1.64028200
H	0.91759300	-2.26508500	-2.02034700

C	3.09153400	-4.35957500	0.42785300
H	3.10512200	-2.56430400	1.61884600
C	-0.72458500	-4.51021700	2.28615000
O	0.95796800	-3.65594900	3.50566800
H	-2.32754800	-5.38700800	1.14878000
C	-5.77311100	-1.16102700	-1.81880500
C	-6.89203100	0.71448000	-0.42568900
C	-4.56624600	-2.75636700	-3.54321200
C	-2.83098000	2.49508000	3.54477000
C	-1.39902300	4.30282000	2.27834600
C	-3.73539100	0.68194300	5.13489900
C	1.09367200	0.35529300	4.42200800
O	-0.37525200	-1.32099700	4.70401200
H	2.47365200	1.99365900	4.20771600
C	5.92945300	1.49626900	-0.59732200
C	7.13985100	-0.72278100	-0.05081700
C	4.76856700	3.84955300	-0.71614500
C	2.62406500	-4.90776000	-0.79344700
C	0.93321100	-4.81744400	-2.81059700
C	3.83635500	-5.22686000	1.45931700
O	-0.25704100	-5.54053800	3.05998000
C	0.53746500	-4.89871100	4.05023900
O	-7.01900400	-1.42524200	-2.35206400
C	-8.11485100	-0.07473400	0.07415300
C	-6.51448900	1.70875800	0.68426000
C	-7.29425400	1.53436500	-1.66476700
C	-3.33275500	-2.38746600	-4.39666500
C	-4.39991400	-4.21191200	-3.07151600
C	-5.76170800	-2.64282000	-4.50479300
O	-3.37639800	3.43161100	4.39954000
C	-2.00886600	4.96369300	1.03027500
C	0.11880400	4.09929800	2.06212900
C	-1.52644200	5.26163700	3.47342900
C	-2.83954600	1.20034800	6.27583500
C	-5.16970200	1.19911800	5.33751400

C	-3.78348400	-0.84964800	5.22987400
O	0.74461600	0.37210500	5.74804400
C	0.14978800	-0.90309400	5.95566300
O	7.18164500	2.03891700	-0.79467300
C	7.87654400	-0.13810500	1.16804900
C	6.78259000	-2.18031700	0.27761000
C	8.09484600	-0.75493500	-1.25696400
C	6.05997200	4.51262400	-0.20521600
C	3.63622700	4.38001900	0.19315200
C	4.46502500	4.31383500	-2.14899200
O	3.06507600	-6.16963300	-1.13583900
C	0.93777400	-6.35220400	-2.89717000
C	1.41036900	-4.22430000	-4.14678900
C	-0.54340500	-4.41003100	-2.60237100
C	5.18621400	-5.74875200	0.93590800
C	4.13131000	-4.43354400	2.74018800
C	2.94262400	-6.42339600	1.83383400
H	1.41090300	-5.51218800	4.27273000
H	-0.07660800	-4.71183100	4.94247400
C	-7.58888600	-2.62140200	-1.84279000
H	-8.84360900	0.62071700	0.50547900
H	-8.60932500	-0.60743500	-0.73847200
H	-7.83253500	-0.78996200	0.85495200
H	-6.25907100	1.19851900	1.62070800
H	-5.67010700	2.34467300	0.39782500
H	-7.37117600	2.36135700	0.88116600
H	-6.47735400	2.19876700	-1.96662300
H	-7.54999000	0.88238500	-2.50361900
H	-8.16580700	2.15753100	-1.43166100
H	-3.43132200	-1.37829700	-4.80202200
H	-2.38788600	-2.44053300	-3.85071000
H	-3.26297000	-3.08964300	-5.23546300
H	-5.24691700	-4.55239300	-2.46717000
H	-4.31215600	-4.87918800	-3.93697700
H	-3.49211300	-4.32252600	-2.46633900

H	-6.68417600	-3.07835300	-4.12059100
H	-5.95581500	-1.59696800	-4.75932700
H	-5.50828300	-3.17687700	-5.42714400
C	-4.51391300	4.06597100	3.83363700
H	-3.09523900	5.06712300	1.12423900
H	-1.59044500	5.96657300	0.88706800
H	-1.80488000	4.37924000	0.12578500
H	0.56062000	3.59686300	2.92933500
H	0.34758500	3.49699800	1.17591400
H	0.61244400	5.07037500	1.94214100
H	-2.53995200	5.63703800	3.62329100
H	-1.19615400	4.79209400	4.40343200
H	-0.88469800	6.12931300	3.28070800
H	-2.88643900	2.28824700	6.35065300
H	-3.16260700	0.77134800	7.23210200
H	-1.79529100	0.92371600	6.09719100
H	-5.60642100	0.72393400	6.22355500
H	-5.19132100	2.27759700	5.49701700
H	-5.80384300	0.95122900	4.47847200
H	-4.46141600	-1.28194500	4.48548200
H	-2.79256400	-1.29463200	5.09469500
H	-4.15778100	-1.13259400	6.21921700
H	0.92372800	-1.61509700	6.27709300
H	-0.65865200	-0.81787600	6.68084900
C	7.49459000	2.27717000	-2.15906000
H	8.77848200	-0.72603900	1.37569200
H	8.17113800	0.89785600	0.98889800
H	7.23630800	-0.17249200	2.05603800
H	6.14040500	-2.25264300	1.16195300
H	6.27406400	-2.67626200	-0.55779400
H	7.70196400	-2.73526600	0.49032600
H	7.56184600	-1.02072800	-2.17669100
H	8.59623600	0.20200800	-1.39936200
H	8.86924700	-1.51169800	-1.08772200
H	5.87365100	5.58623600	-0.09478800

H	6.35008400	4.11419500	0.77203600
H	6.90837300	4.39522800	-0.87867600
H	2.64107400	4.11831900	-0.17572200
H	3.74261100	4.00100800	1.21670900
H	3.68040400	5.47415400	0.22549400
H	5.28275500	4.10405300	-2.84353300
H	3.57378800	3.81307900	-2.53489300
H	4.28226700	5.39470000	-2.16621300
C	4.07231000	-6.13126300	-2.13558700
H	0.68635700	-6.80667500	-1.93520800
H	0.18137600	-6.65484000	-3.62977400
H	1.89212400	-6.76530000	-3.22653500
H	1.33775400	-3.13147400	-4.14381600
H	2.45063000	-4.49010000	-4.36150300
H	0.78875200	-4.60224800	-4.96640700
H	-0.91749900	-4.78147500	-1.64112800
H	-0.67823200	-3.32366800	-2.61950000
H	-1.15841800	-4.83142900	-3.40600200
H	5.73800700	-6.21102500	1.76246800
H	5.05693900	-6.50626800	0.16276700
H	5.80071100	-4.93126800	0.54126700
H	4.82721300	-3.60775600	2.55313100
H	3.22123300	-4.02308700	3.18626800
H	4.60357200	-5.10040000	3.46913000
H	2.80031000	-7.09130900	0.98196500
H	3.40584000	-6.99434300	2.64782500
H	1.95572100	-6.08218100	2.16112200
H	-8.58921600	-2.69999700	-2.27445600
H	-7.00087800	-3.49809900	-2.13050300
H	-7.66018000	-2.58995200	-0.75011900
H	-4.89619400	4.75628100	4.58870300
H	-4.25022200	4.62373800	2.92857200
H	-5.28639000	3.33034600	3.57933200
H	6.94796900	3.13894700	-2.55023900
H	8.56654700	2.48324000	-2.20182900

H	7.26465000	1.40054100	-2.77417900
H	4.37497700	-7.16530100	-2.31492500
H	3.69453600	-5.69469700	-3.06600100
H	4.93634100	-5.54611500	-1.79963300
C	-1.76608300	0.85742200	-4.16855200
C	-2.95654700	1.05283400	-3.45632300
C	-1.84628400	0.74902400	-5.55958400
C	-4.18073600	1.13480500	-4.10721900
H	-2.91936700	1.14182800	-2.37302300
C	-3.07255500	0.84608100	-6.21897200
H	-0.94741900	0.59870100	-6.14839500
C	-4.24529600	1.03770800	-5.49802400
H	-5.08946900	1.26411500	-3.52625800
H	-3.10445400	0.76664500	-7.30187900
H	-5.20119000	1.10647700	-6.00898400
C	0.74037200	0.40758400	-4.29879700
H	1.63484800	0.26987000	-3.68386800
H	0.51981500	-0.54567100	-4.78966400
H	0.97437100	1.14943500	-5.07278300
H	-1.13905600	2.69093700	-2.57214000
C	1.49022900	6.39353900	-1.83920200
C	0.84684800	6.33259900	-0.60461500
C	2.17028400	7.55086200	-2.22016800
C	0.88910400	7.42735100	0.25248600
H	0.32578500	5.42343600	-0.32818300
C	2.21745300	8.64156800	-1.35871000
H	2.65535800	7.57197500	-3.19121600
C	1.57706700	8.58033000	-0.12200400
H	0.38714800	7.38154000	1.21596000
H	2.75240900	9.54008900	-1.65014300
H	1.61311700	9.43269100	0.54985700

References

1. (a) W. Xiong, G. Xu, X. Yu and W. Tang, *Organometallics*, 2019, **38**, 4003; (b) M. Takeda, K. Takatsu, R. Shintani and T. Hayashi, *J. Org. Chem.*, 2014, **79**, 2354.

2. D. Wang, X.-S. Xue, K. N. Houk and Z. Shi, *Angew. Chem. Int. Ed.*, 2018, **57**, 16861.

3. D. A. Gandamana, B. Wang, C. Tejo, B. Bolte, F. Gagosz and S. Chiba, *Angew. Chem. Int. Ed.*, 2018, **57**, 6181.

4. S. Movahhed, J. Westphal, M. Dindaroğlu, A. Falk and H.-G. Schmalz, *Chem. Eur. J.* 2016, **22**, 7381.

5. Q. Wang, X. Liu, X. Liu, B. Li, H. Nie, S. Zhang and W. Chen, *Chem. Commun.*, 2014, **50**, 978.

6. A. Krasovskiy, C. Duplais and B. H. Lipshutz, *J. Am. Chem. Soc.*, 2009, **131**, 15592.

7. M. J. Frisch, G. W. Trucks, H. B. Schlegel, G. E. Scuseria, M. A. Robb, J. R. Cheeseman, G. Scalmani, V. Barone, B. Mennucci, G. A. Petersson, H. Nakatsuji, M. Caricato, X. Li, H. P. Hratchian, A. F. Izmaylov, J. Bloino, G. Zheng, J. L. Sonnenberg, M. Hada, M. Ehara, K. Toyota, R. Fukuda, J. Hasegawa, M. Ishida, T. Nakajima, Y. Honda, O. Kitao, H. Nakai, T. Vreven, Jr., J. A. Montgomery, J. E. Peralta, F. Ogliaro, M. Bearpark, J. J. Heyd, E. Brothers, K. N. Kudin, V. N. Staroverov, R. Kobayashi, J. Normand, K. Raghavachari, A. Rendell, J. C. Burant, S. S. Iyengar, J. Tomasi, M. Cossi, N. Rega, J. M. Millam, M. Klene, J. E. Knox, J. B. Cross, V. Bakken, C. Adamo, J. Jaramillo, R. Gomperts, R. E. Stratmann, O. Yazyev, A. J. Austin, R. Cammi, C. Pomelli, J. W. Ochterski, R. L. Martin, K. Morokuma, V. G. Zakrzewski, G. A. Voth, P. Salvador, J. J. Dannenberg, S. Dapprich, A. D. Daniels, Ö. Farkas, J. B. Foresman, J. V. Ortiz, J. Cioslowski and D. J. Fox, Gaussian, Inc., Wallingford, CT, 2009.

8. C. Y. Legault, *CYLView, 1.0b.* University of Sherbrooke **2009**.

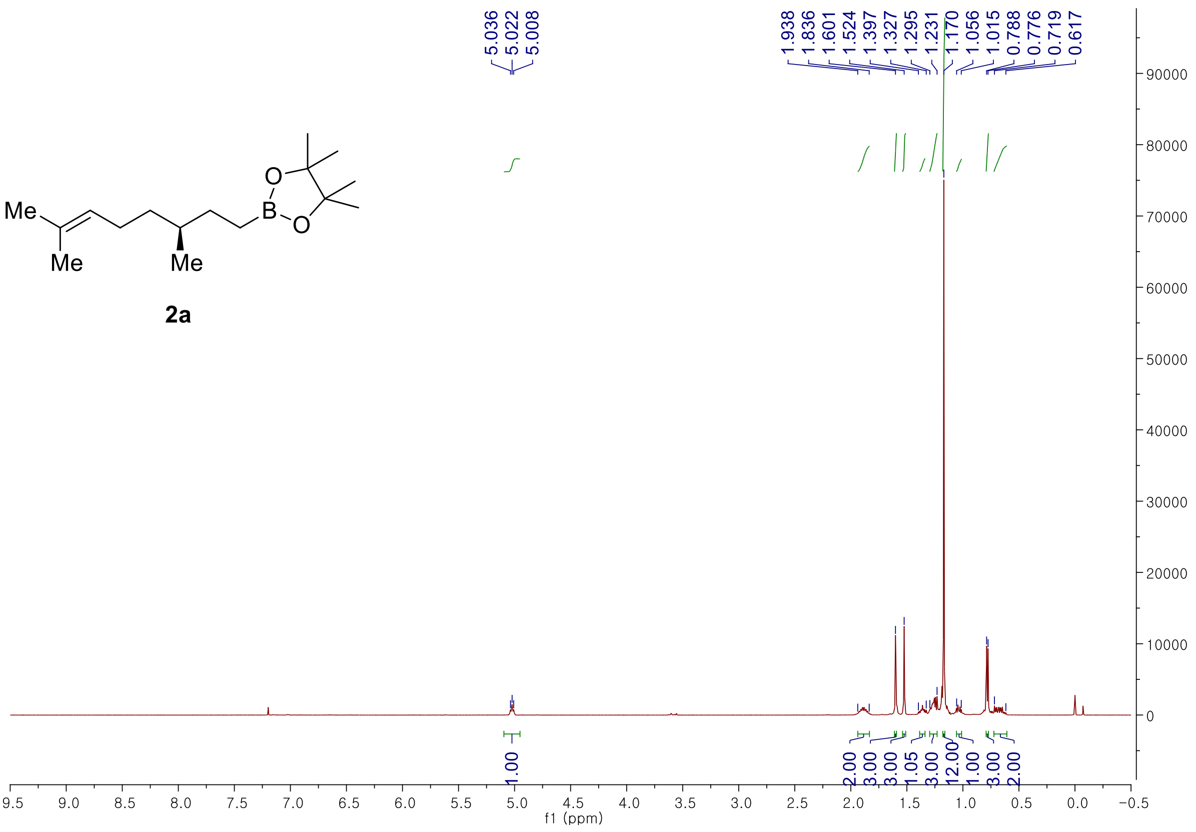
Me
Me
Me
O
B
O
5.036
5.022
5.008
1.938
1.836
1.601
1.524
1.397
1.327
1.295
1.231
1.170
1.056
1.015
0.788
0.776
0.719
0.617
1.00
2.00
3.00
3.00
1.05
3.00
12.00
1.00
3.00
2.00
f1 (ppm)

2a

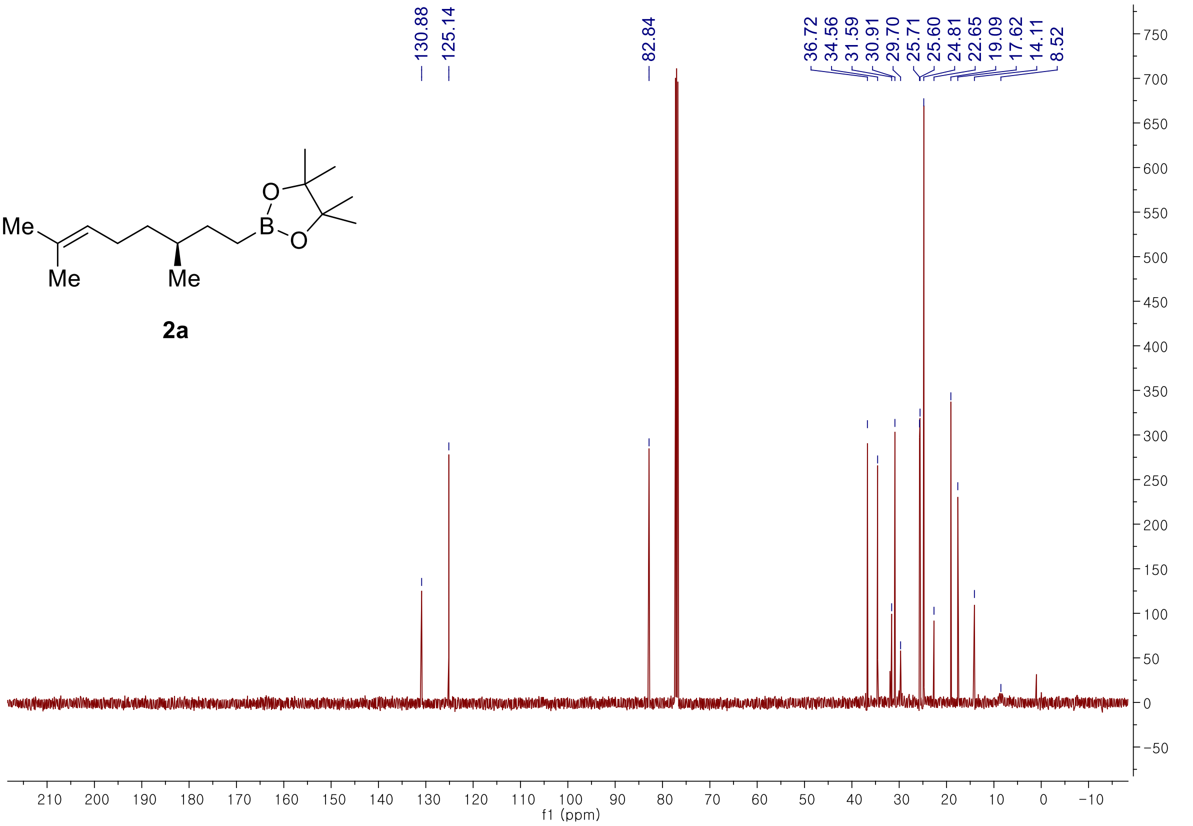
Me
Me
Me
O
B
O
130.88
125.14
82.84
36.72
34.56
31.59
30.91
29.70
25.71
25.60
24.81
22.65
19.09
17.62
14.11
8.52
f1 (ppm)

2a

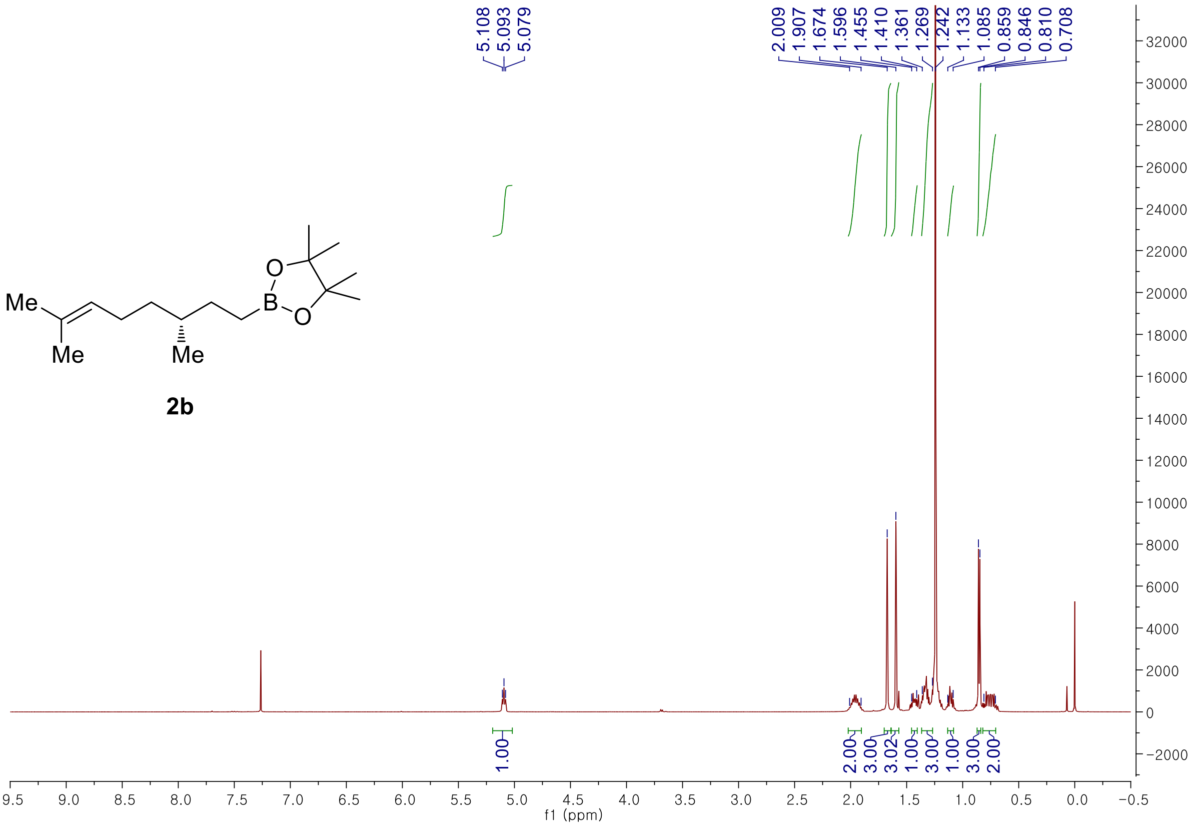

5.108
5.093
5.079
2.009
1.907
1.674
1.596
1.455
1.410
1.361
1.269
1.242
1.133
1.085
0.859
0.846
0.810
0.708
Me
Me
Me
O
B
O
1.00
2.00
3.00
3.02
1.00
3.00
1.00
3.00
2.00
f1 (ppm)

2b

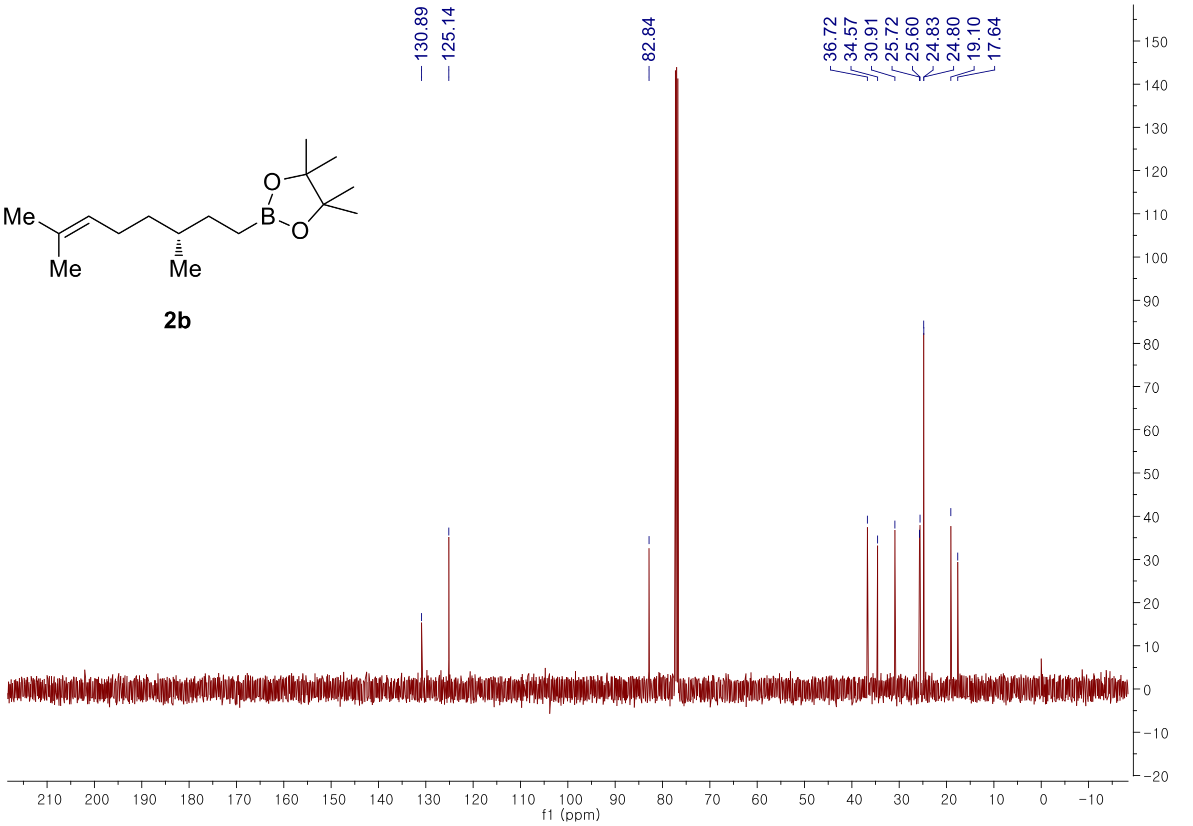

130.89
125.14
82.84
36.72
34.57
30.91
25.72
25.60
24.83
24.80
19.10
17.64
Me
Me
Me
O
B
O
f1 (ppm)

2b

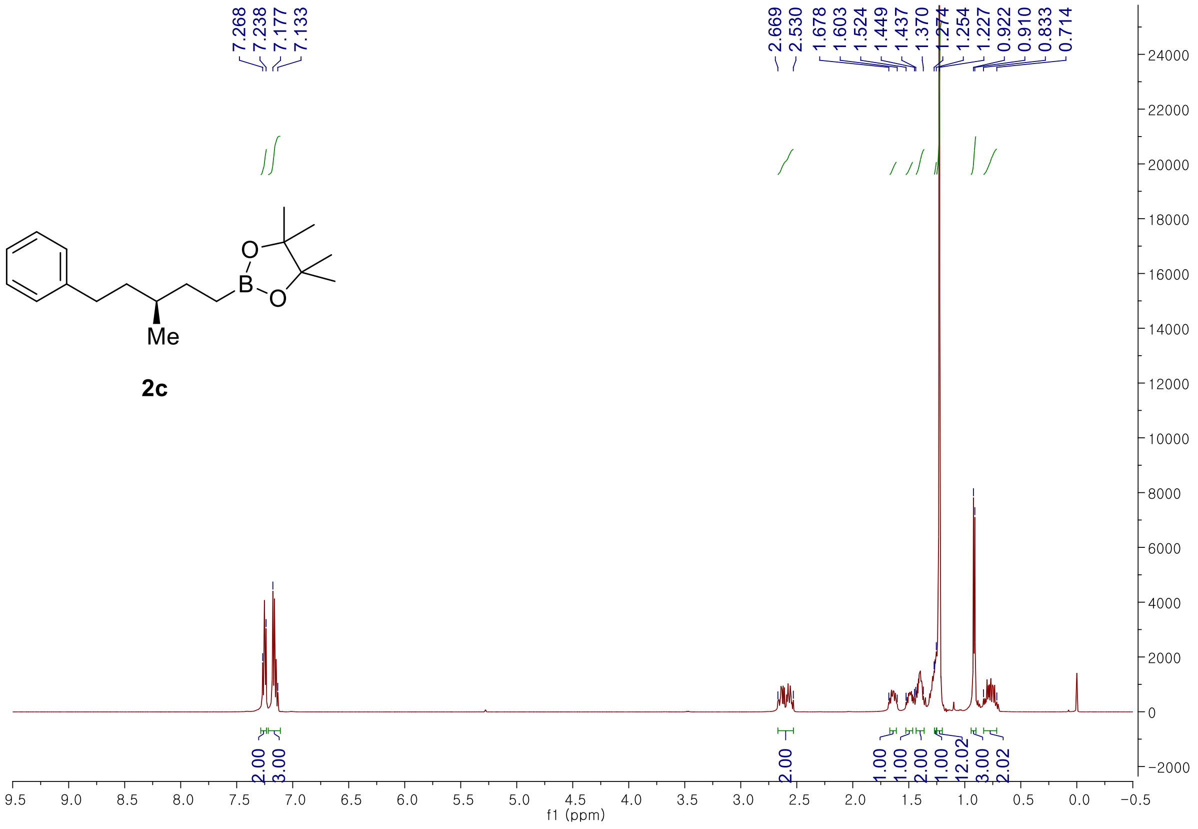
7.268
7.238
7.177
7.133
2.669
2.530
1.678
1.603
1.524
1.449
1.437
1.370
1.274
1.254
1.227
0.922
0.910
0.833
0.714
O
B
O
Me
2c
2.00
3.00
2.00
1.00
1.00
2.00
1.00
12.02
3.00
2.02
f1 (ppm)

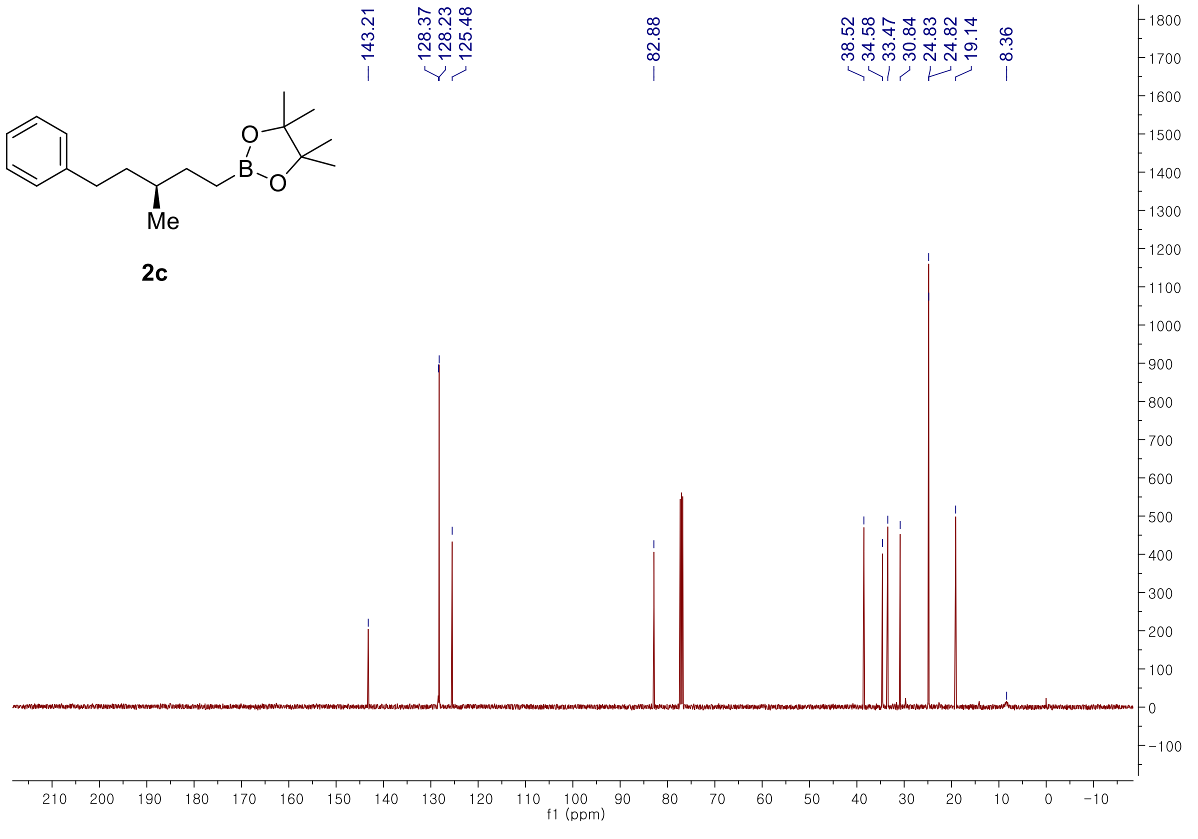
143.21
128.37
128.23
125.48
82.88
38.52
34.58
33.47
30.84
24.83
24.82
19.14
8.36
O
B
O
Me
2c
f1 (ppm)

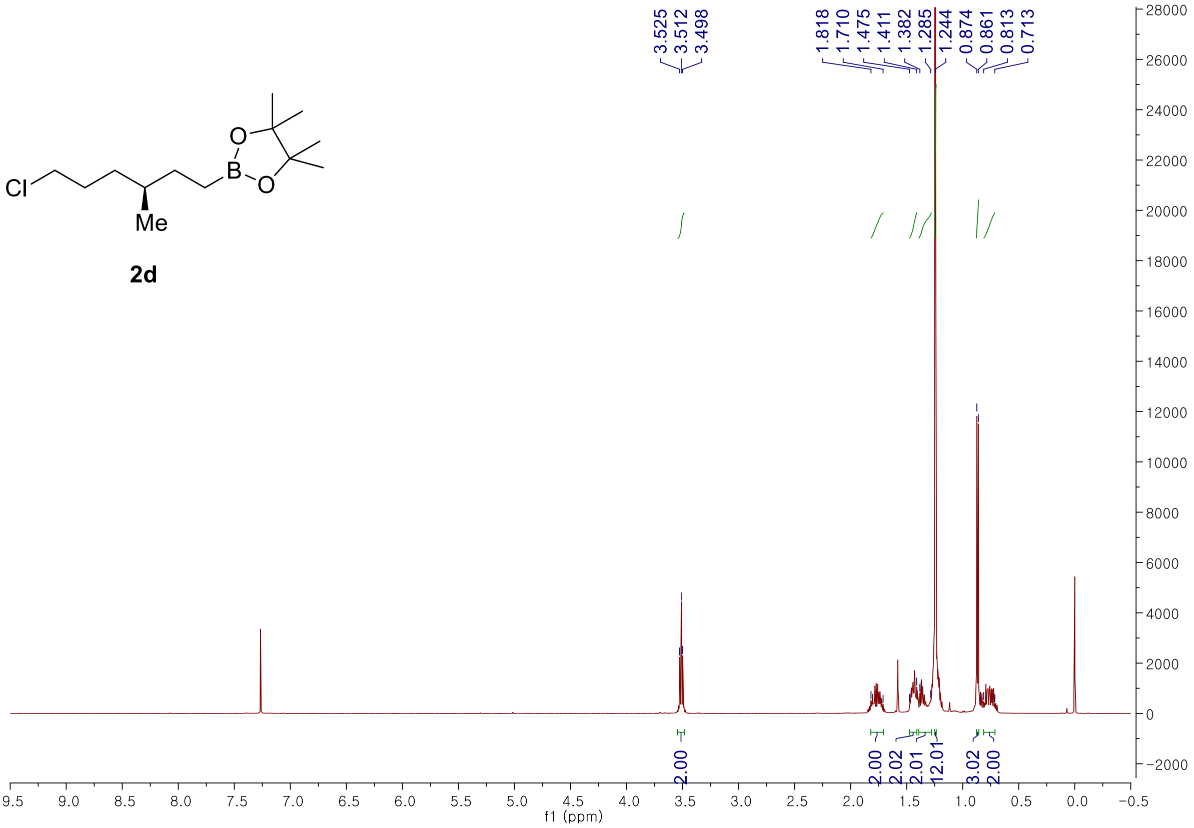
Cl
O
B
O
Me
2d
3.525
3.512
3.498
1.818
1.710
1.475
1.411
1.382
1.285
1.244
0.874
0.861
0.813
0.713
2.00
2.00
2.02
2.01
12.01
3.02
2.00
f1 (ppm)

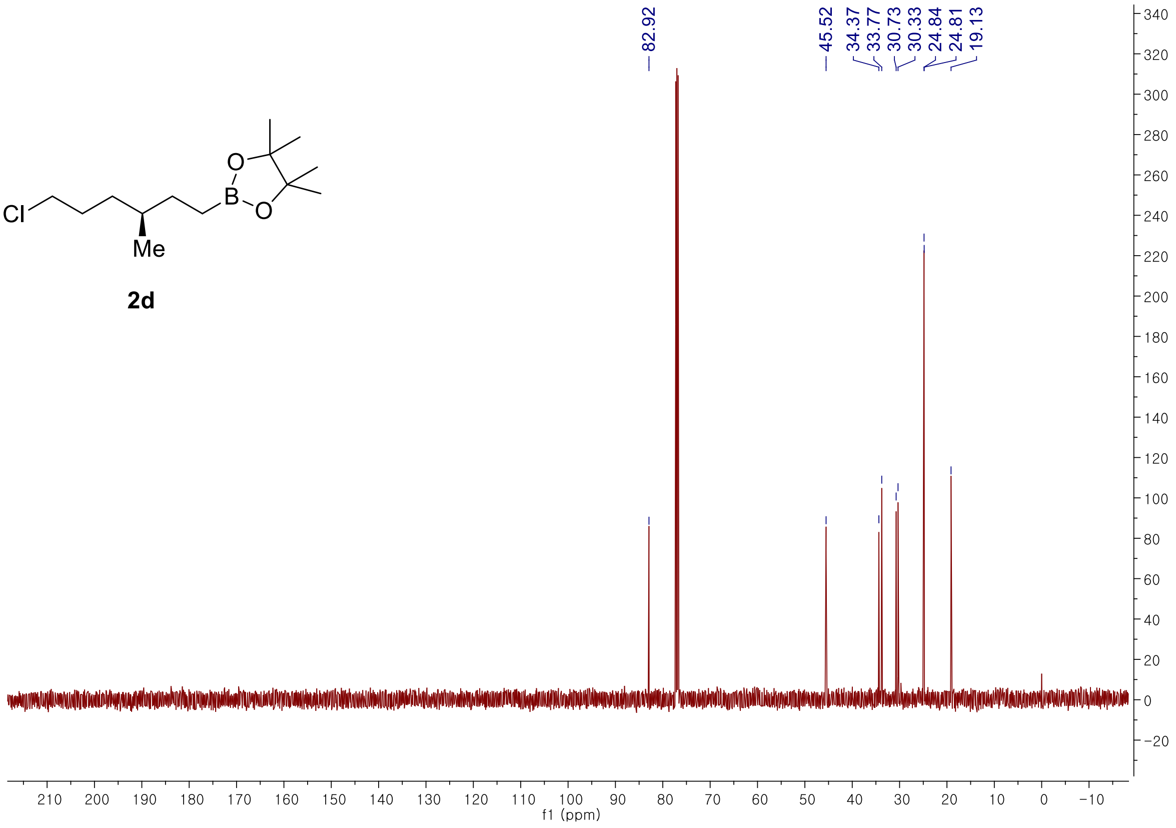
Cl
O
B
O
Me
2d
82.92
45.52
34.37
33.77
30.73
30.33
24.84
24.81
19.13
f1 (ppm)

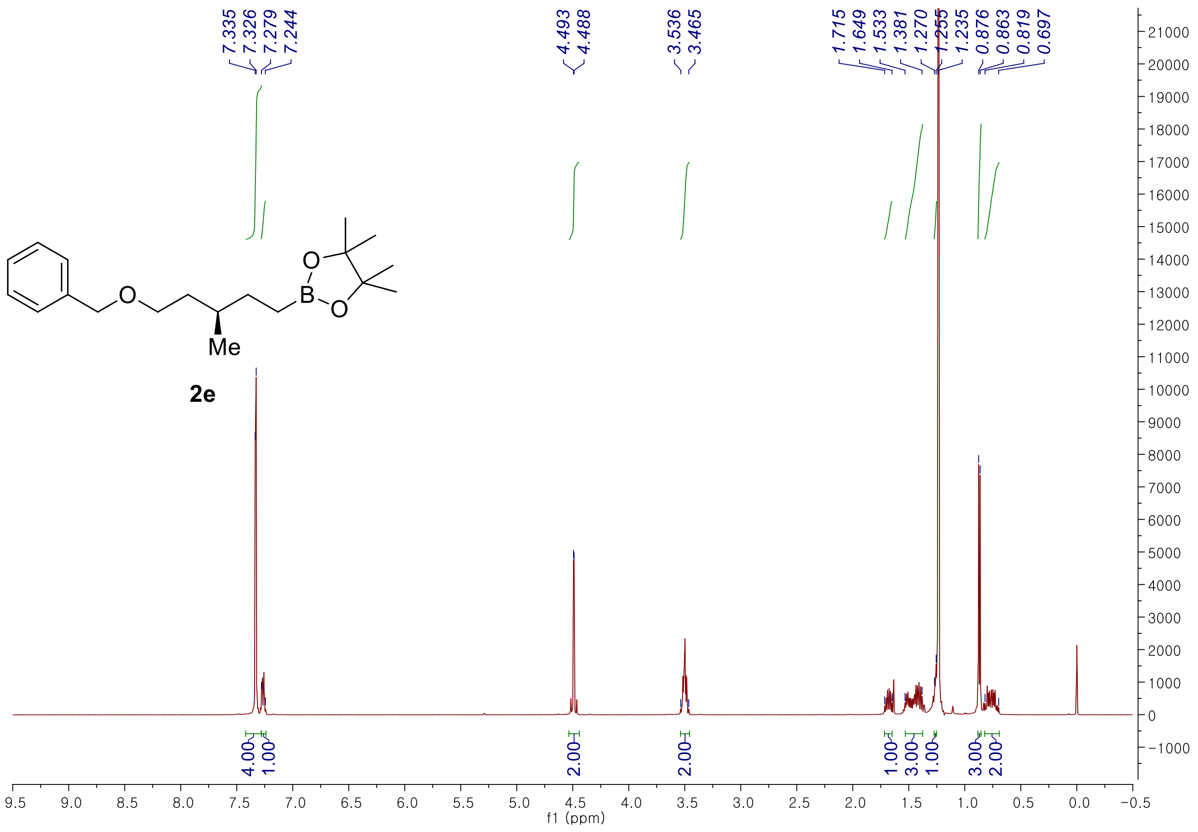
7.335
7.326
7.279
7.244
4.493
4.488
3.536
3.465
1.715
1.649
1.533
1.381
1.270
1.255
1.235
0.876
0.863
0.819
0.697
O
B
O
O
Me
2e
4.00
1.00
2.00
2.00
1.00
3.00
1.00
3.00
2.00
f1 (ppm)

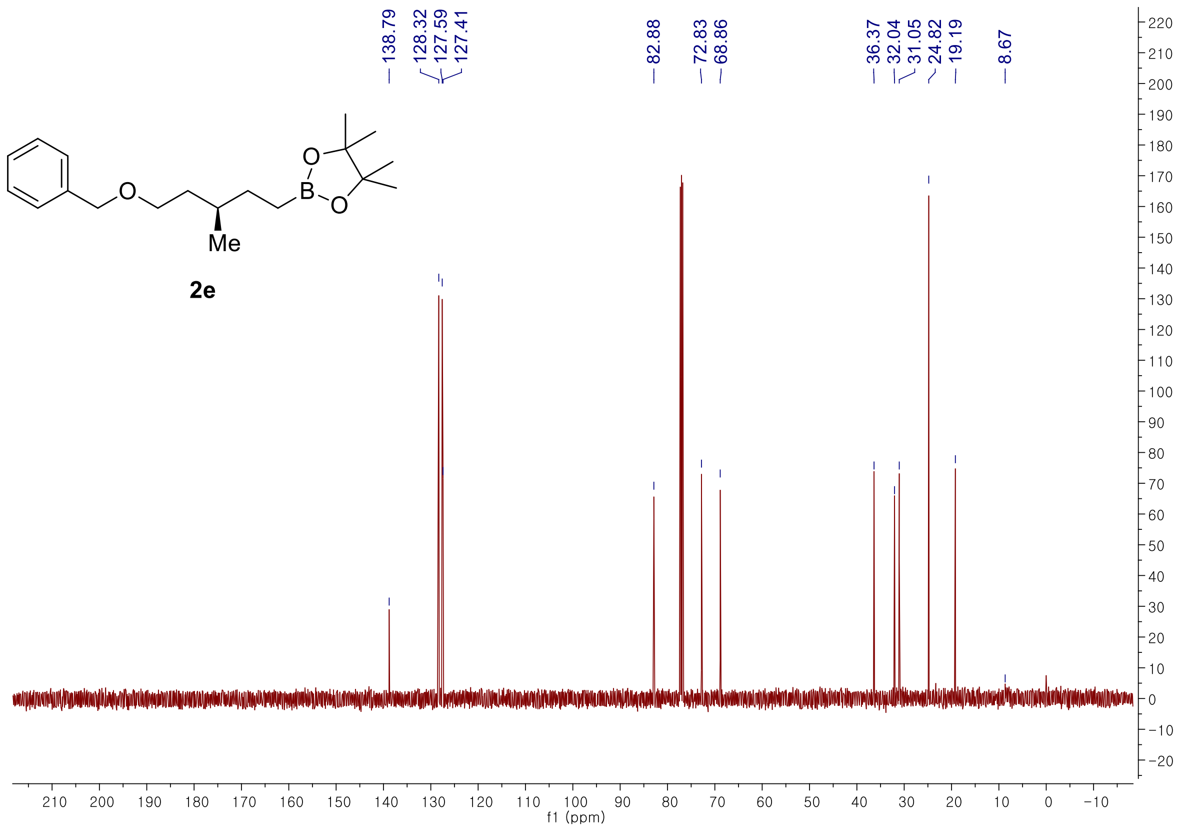
138.79
128.32
127.59
127.41
82.88
72.83
68.86
36.37
32.04
31.05
24.82
19.19
8.67
O
B
O
O
Me
2e
f1 (ppm)

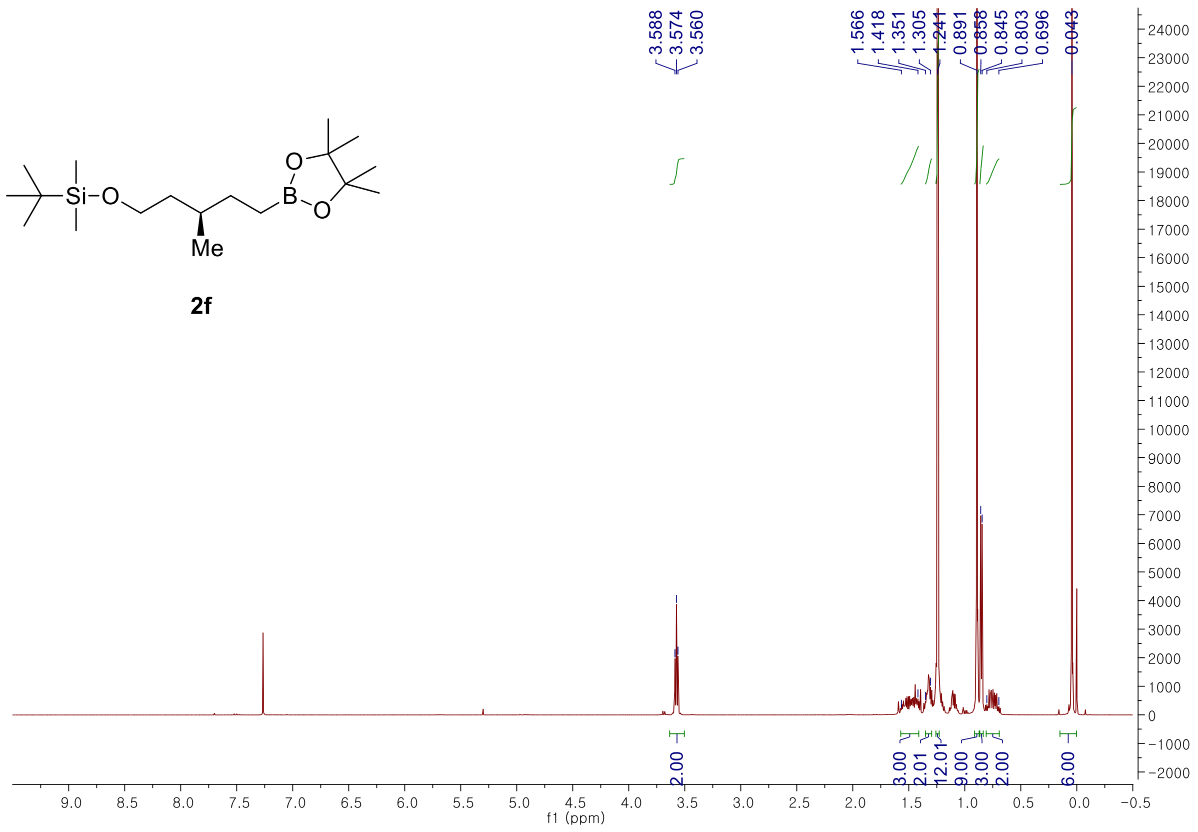
3.588
3.574
3.560
1.566
1.418
1.351
1.305
1.241
0.891
0.858
0.845
0.803
0.696
0.043
2.00
3.00
2.01
12.01
9.00
3.00
2.00
6.00
f1 (ppm)
Si
O
B
Me

2f

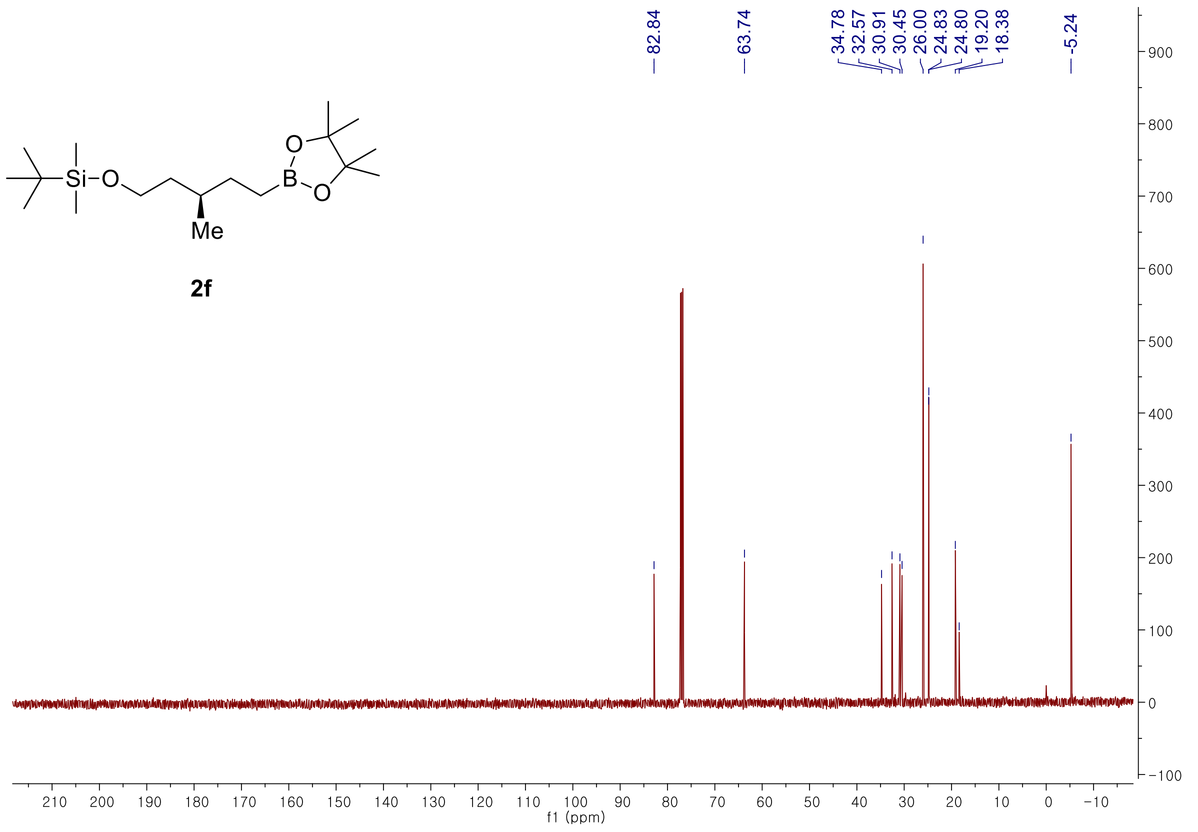
82.84
63.74
34.78
32.57
30.91
30.45
26.00
24.83
24.80
19.20
18.38
-5.24
f1 (ppm)
Si
O
B
Me

2f

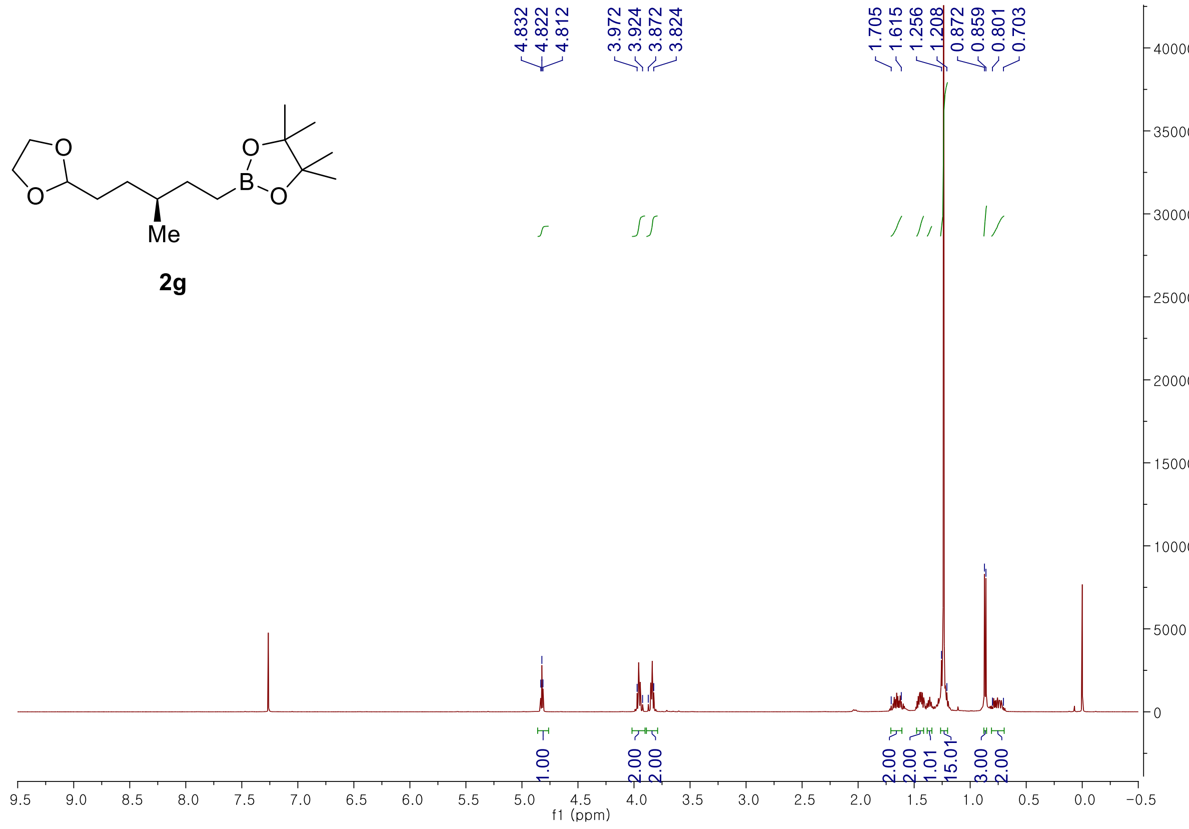
4.832
4.822
4.812
3.972
3.924
3.872
3.824
1.705
1.615
1.256
1.208
0.872
0.859
0.801
0.703
1.00
2.00
2.00
2.00
2.00
1.01
15.01
3.00
2.00
f1 (ppm)
Me
2g

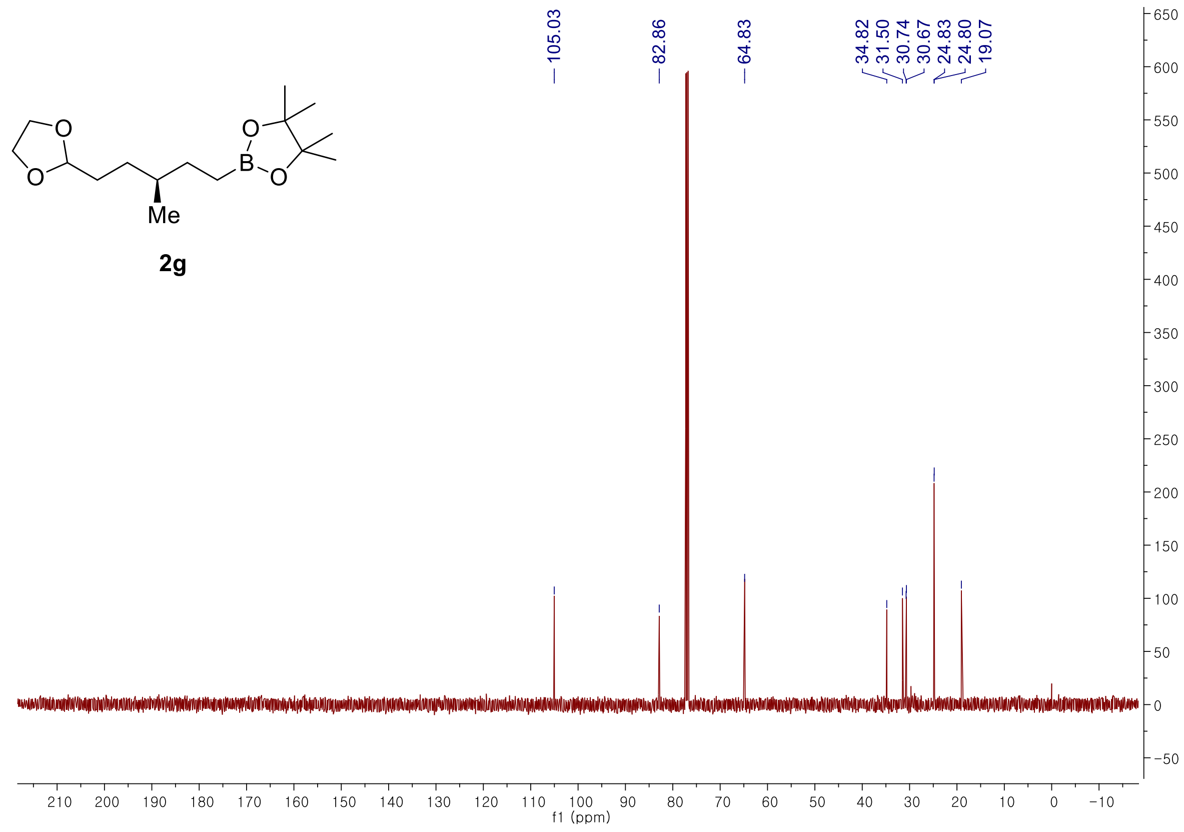
105.03
82.86
64.83
34.82
31.50
30.74
30.67
24.83
24.80
19.07
f1 (ppm)
Me
2g

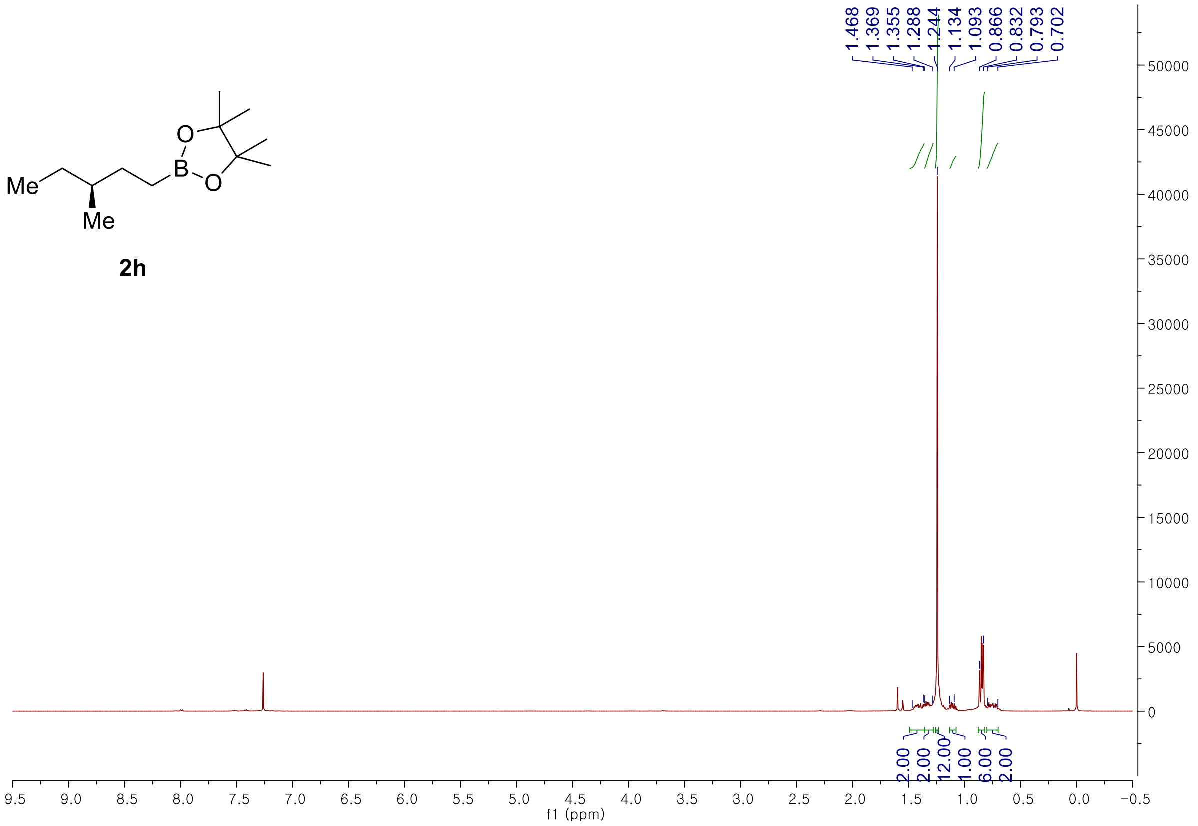

Me
Me
O
B
O
1.468
1.369
1.355
1.288
1.244
1.134
1.093
0.866
0.832
0.793
0.702
2.00
2.00
12.00
1.00
6.00
2.00
f1 (ppm)

2h

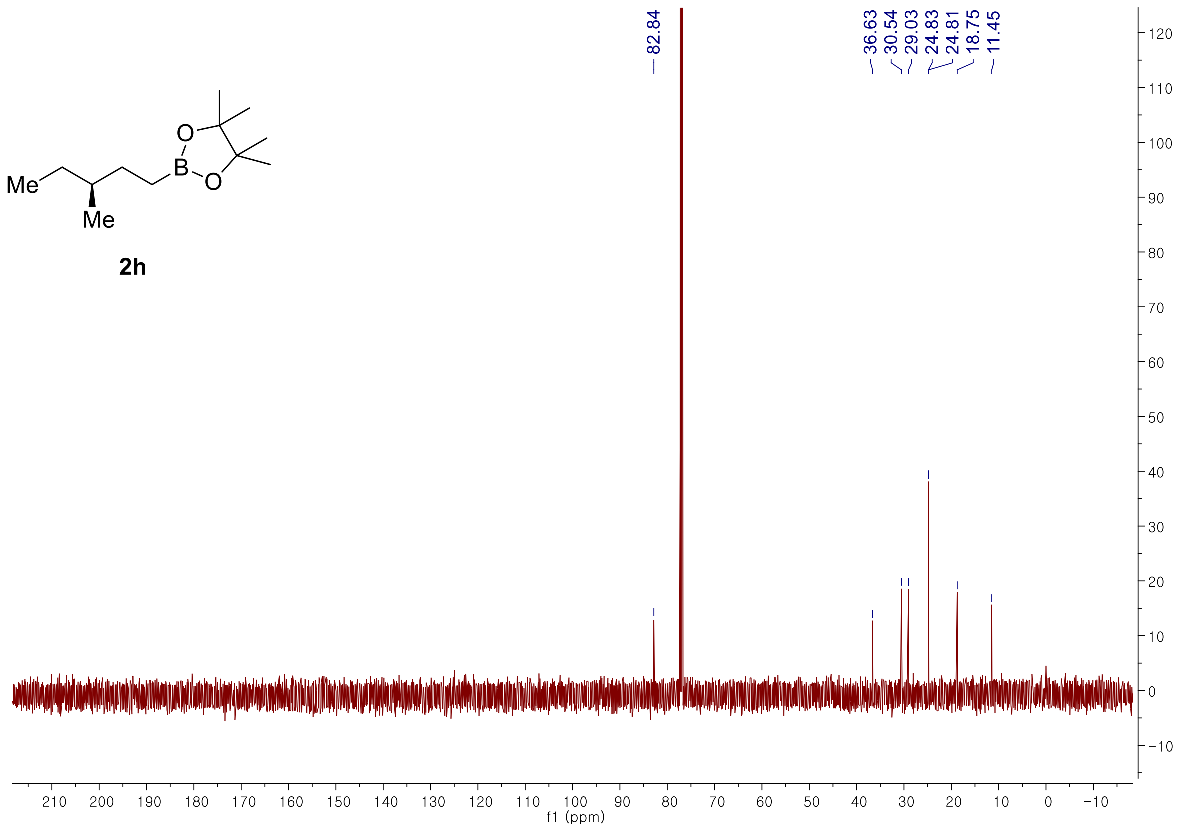

Me
Me
O
B
O
82.84
36.63
30.54
29.03
24.83
24.81
18.75
11.45
f1 (ppm)

2h

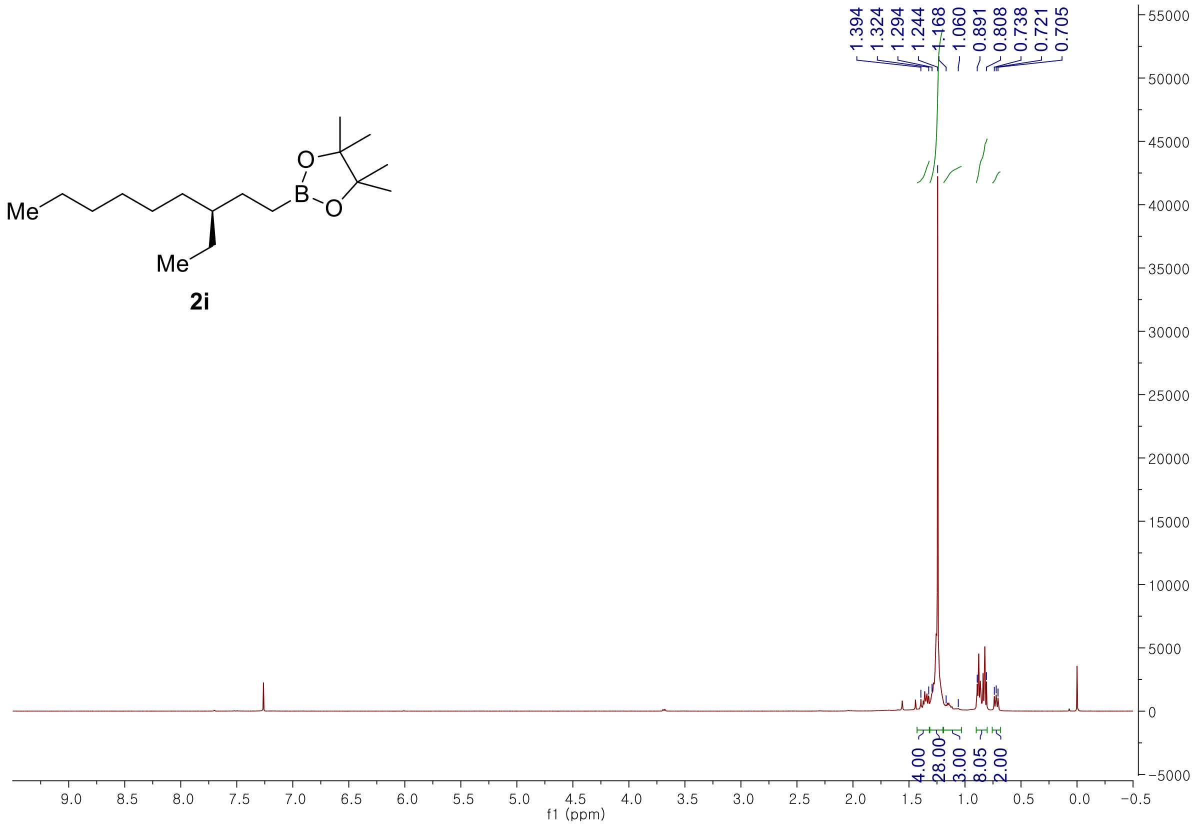

Me
O
B
O
Me
2i
1.394
1.324
1.294
1.244
1.168
1.060
0.891
0.808
0.738
0.721
0.705
4.00
28.00
3.00
8.05
2.00
f1 (ppm)

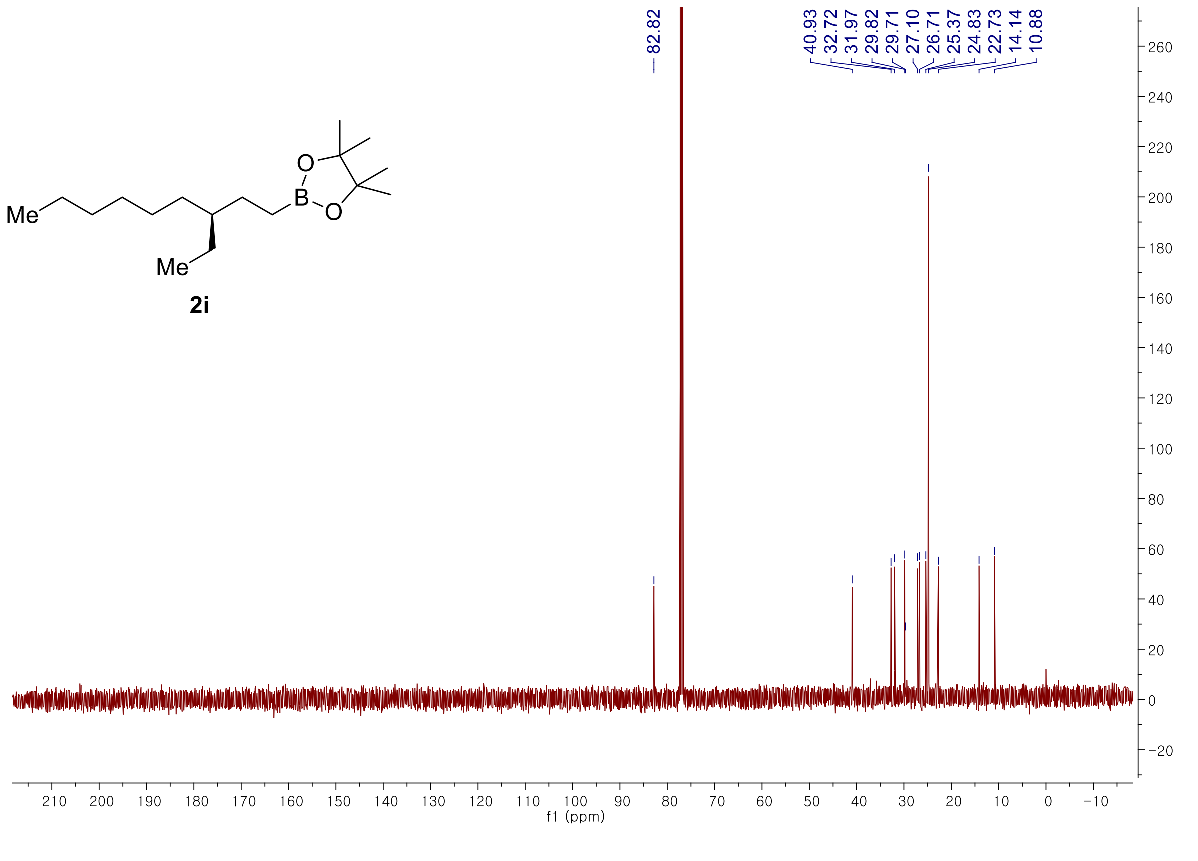

Me
O
B
O
Me
2i
82.82
40.93
32.72
31.97
29.82
29.71
27.10
26.71
25.37
24.83
22.73
14.14
10.88
f1 (ppm)

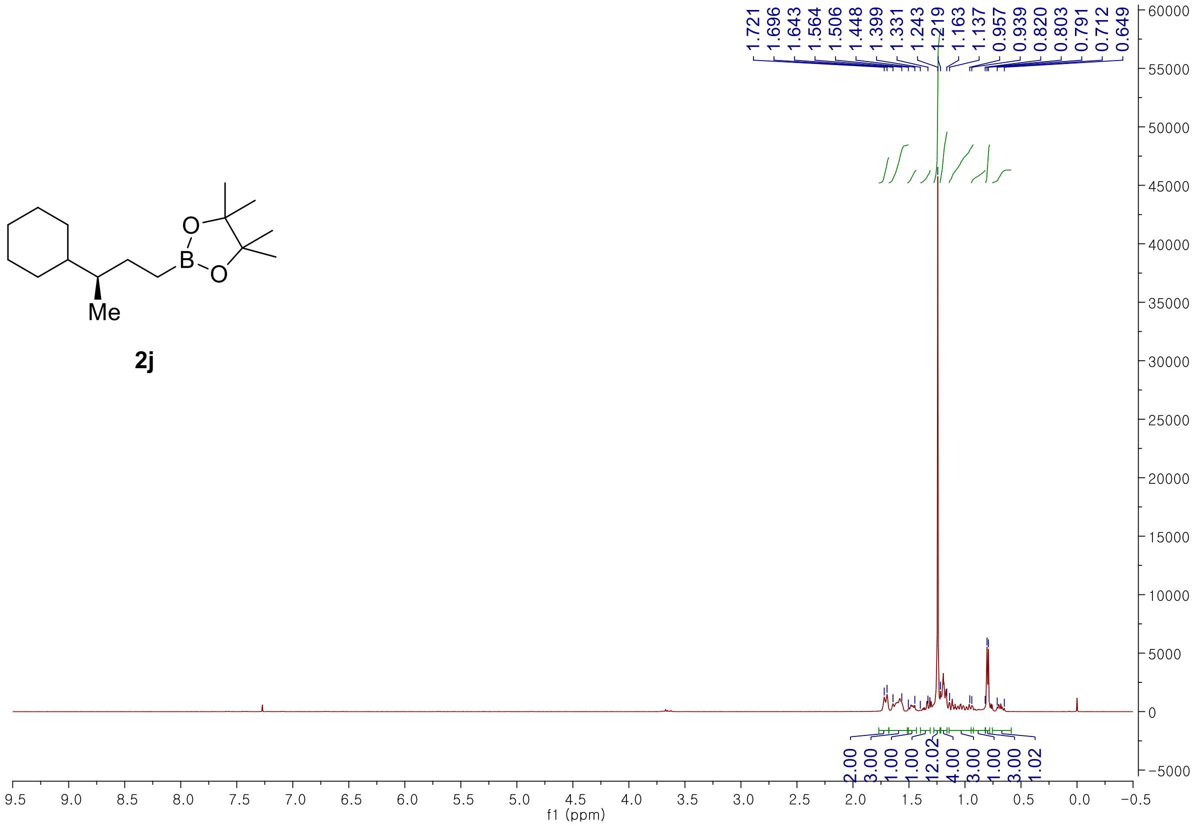

O
B
O
Me
1.721
1.696
1.643
1.564
1.506
1.448
1.399
1.331
1.243
1.219
1.163
1.137
0.957
0.939
0.820
0.803
0.791
0.712
0.649
2.00
3.00
1.00
1.00
12.02
4.00
3.00
1.00
3.00
1.02
f1 (ppm)

2j

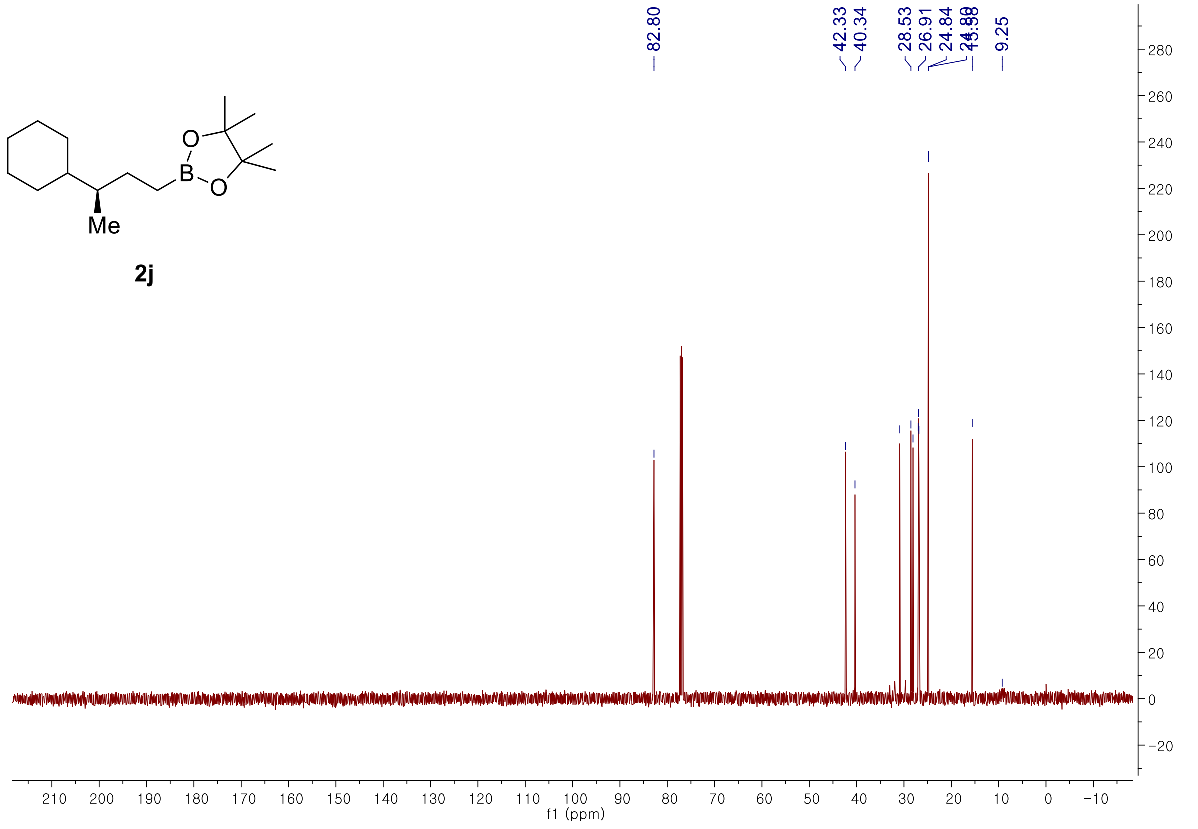

O
B
O
Me
82.80
42.33
40.34
28.53
26.91
24.84
15.98
9.25
f1 (ppm)

2j

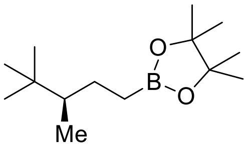

Me

2k

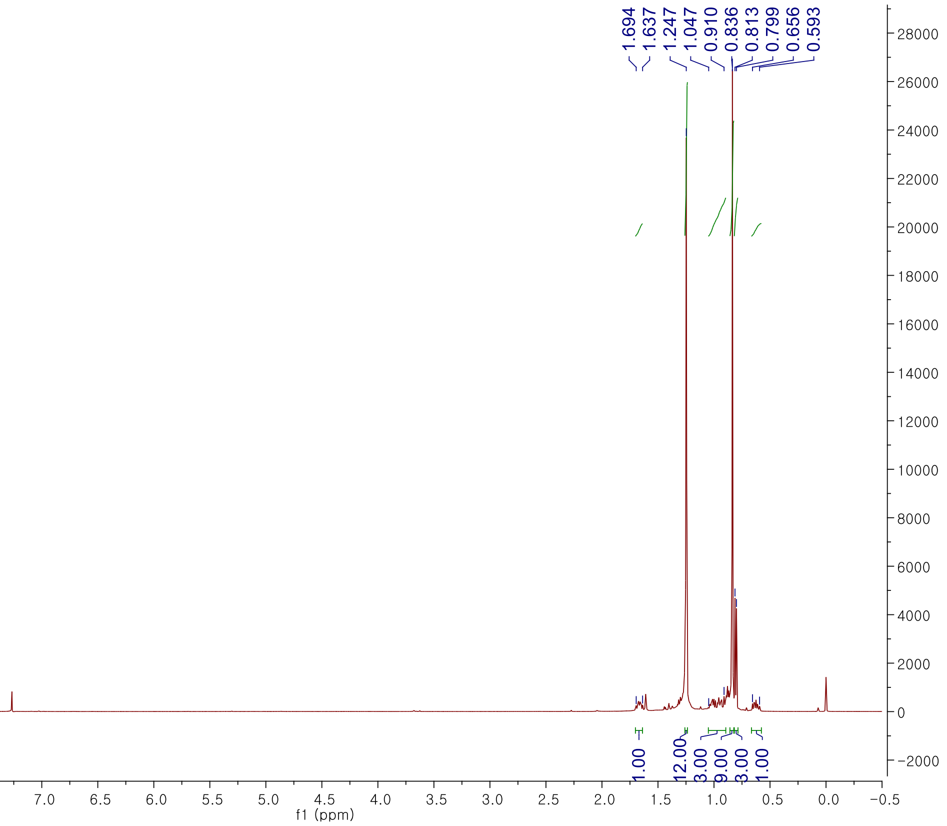

1.694
1.637
1.247
1.047
0.910
0.836
0.813
0.799
0.656
0.593
1.00
12.00
3.00
9.00
3.00
1.00
f1 (ppm)

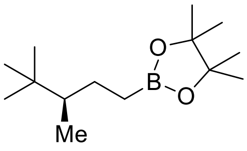

Me

2k

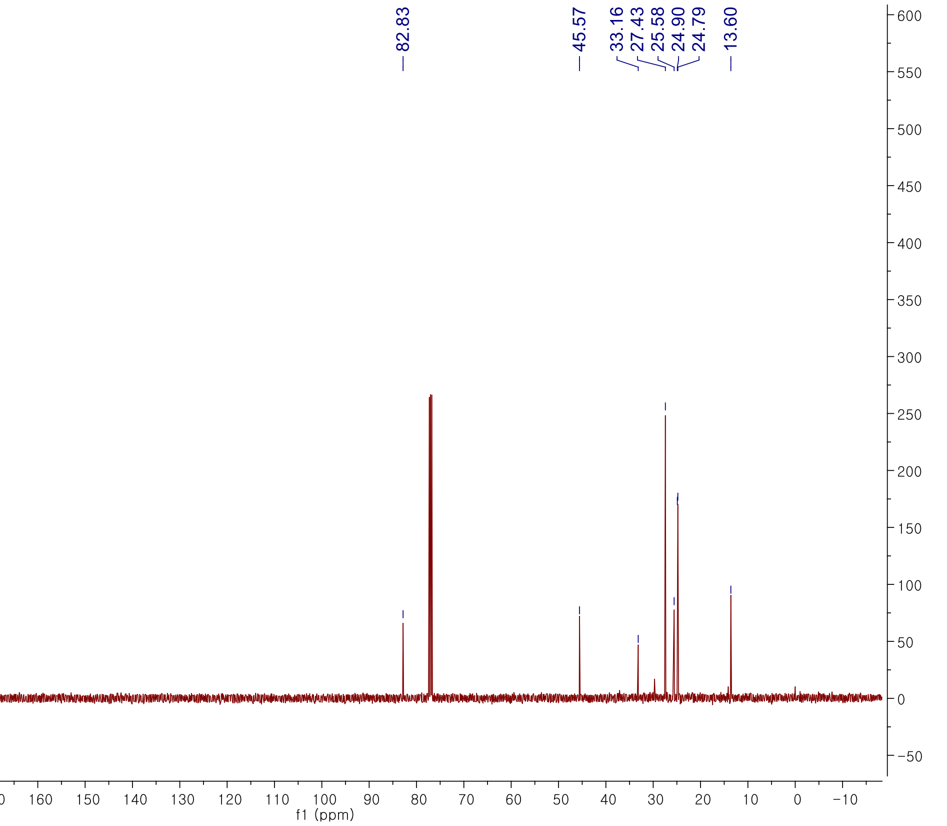

82.83
45.57
33.16
27.43
25.58
24.90
24.79
13.60
f1 (ppm)

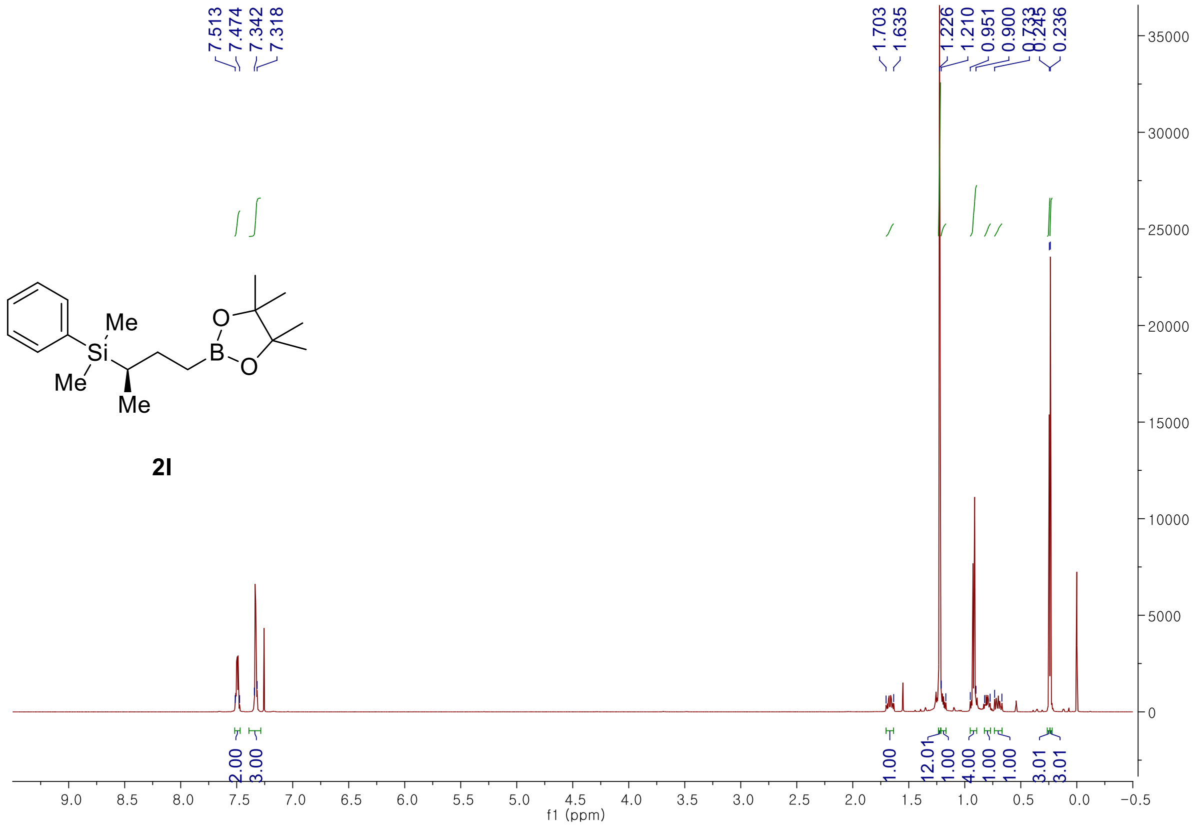
7.513
7.474
7.342
7.318
1.703
1.635
1.226
1.210
0.951
0.900
0.733
0.245
0.236
2.00
3.00
1.00
12.01
1.00
4.00
1.00
1.00
3.01
3.01
f1 (ppm)

2l

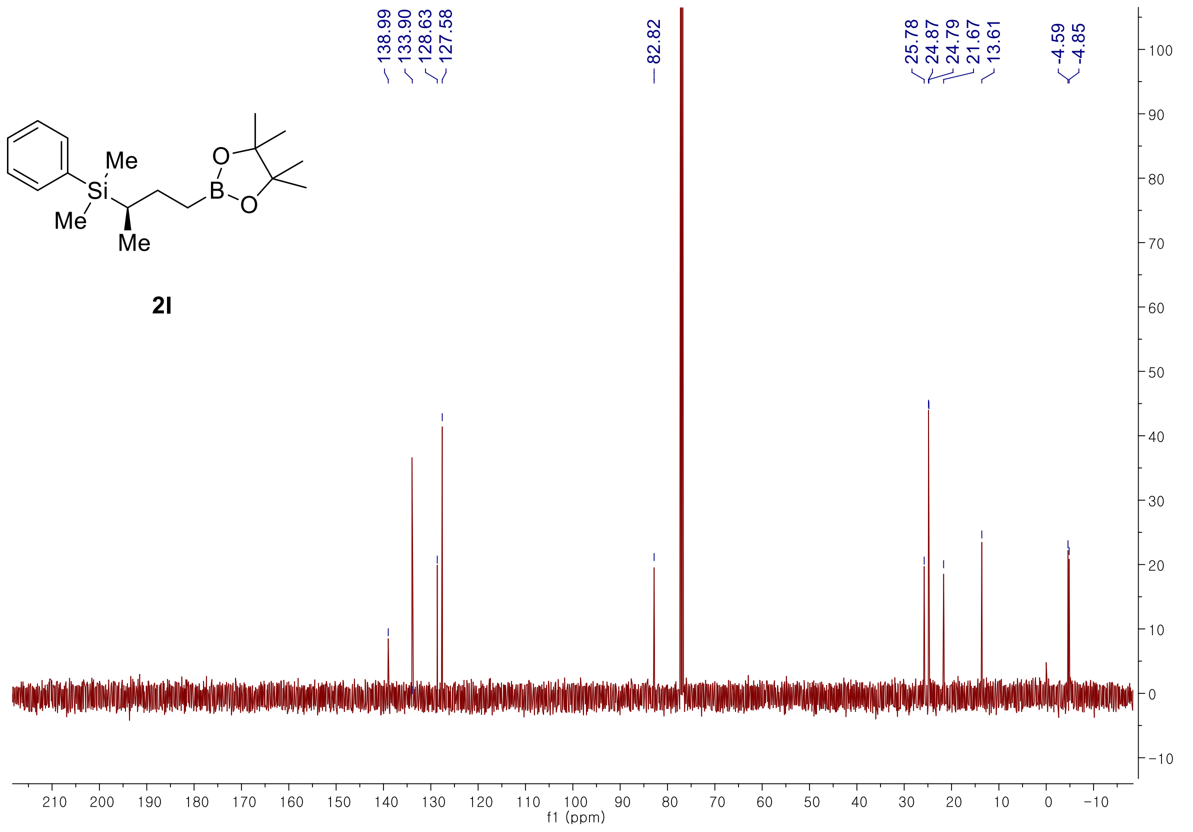
138.99
133.90
128.63
127.58
82.82
25.78
24.87
24.79
21.67
13.61
-4.59
-4.85
f1 (ppm)

2l

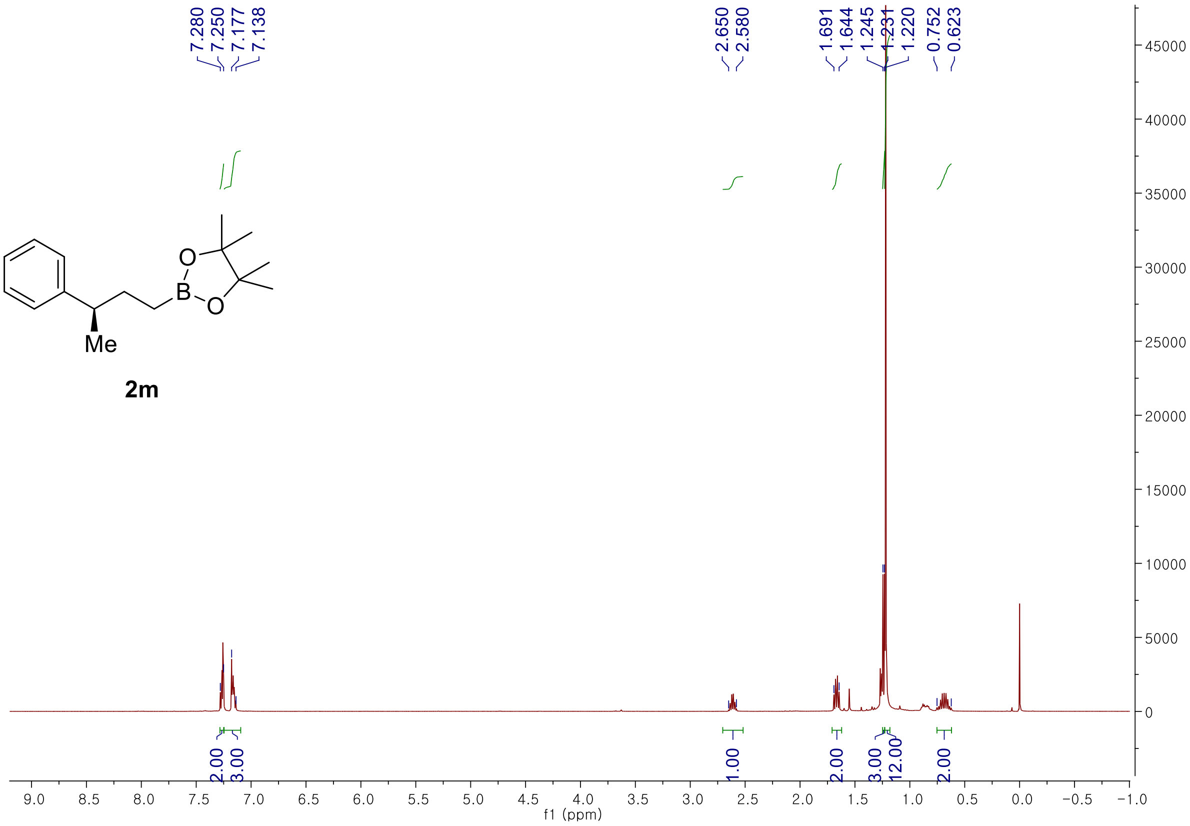
7.280
7.250
7.177
7.138
2.650
2.580
1.691
1.644
1.245
1.231
1.220
0.752
0.623
2.00
3.00
1.00
2.00
3.00
12.00
2.00
f1 (ppm)

2m

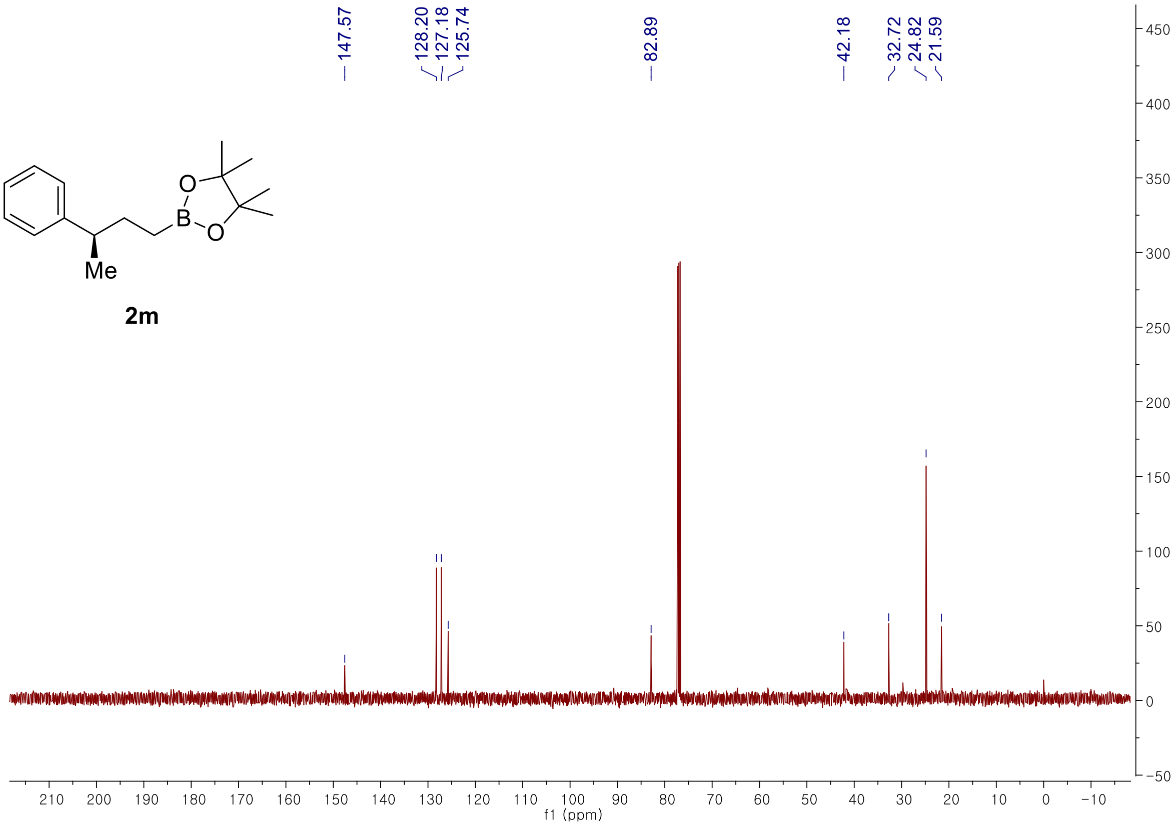
147.57
128.20
127.18
125.74
82.89
42.18
32.72
24.82
21.59
f1 (ppm)

2m

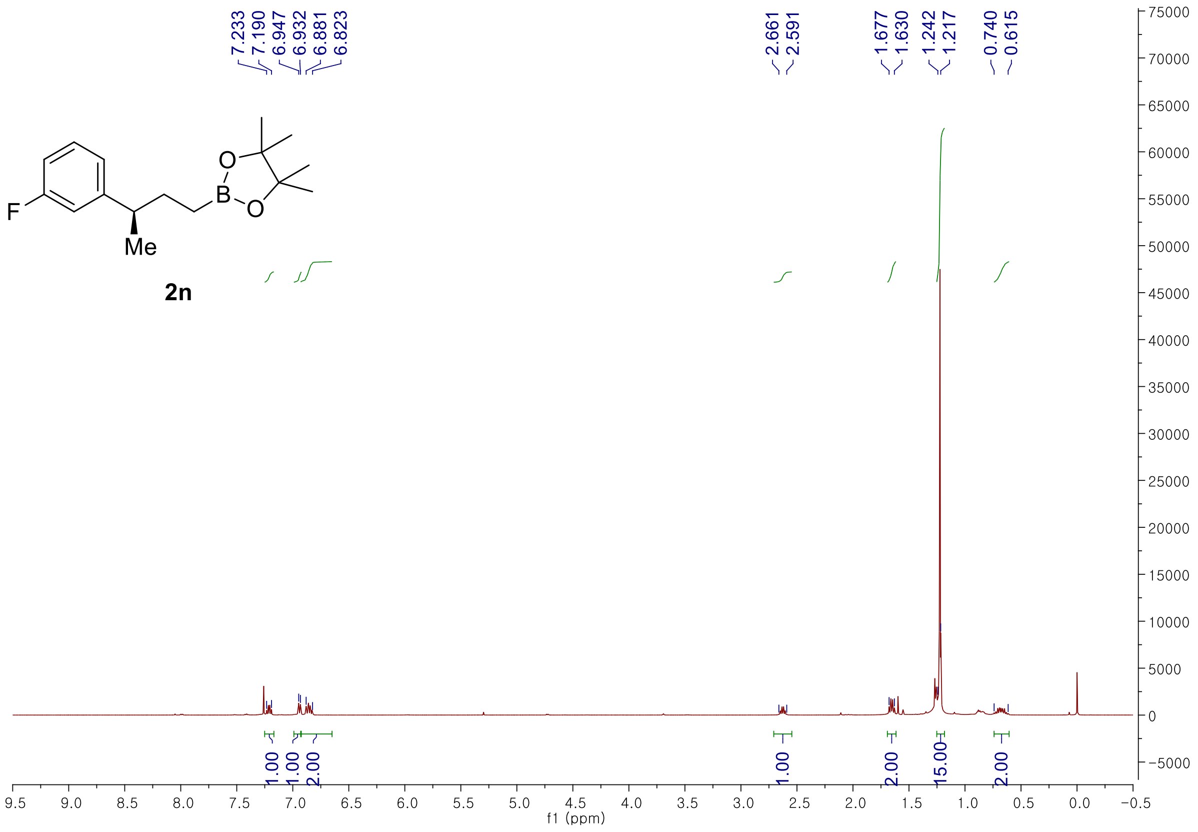
7.233
7.190
6.947
6.932
6.881
6.823
2.661
2.591
1.677
1.630
1.242
1.217
0.740
0.615
F
O
B
O
Me
2n
1.00
1.00
2.00
1.00
2.00
15.00
2.00
f1 (ppm)

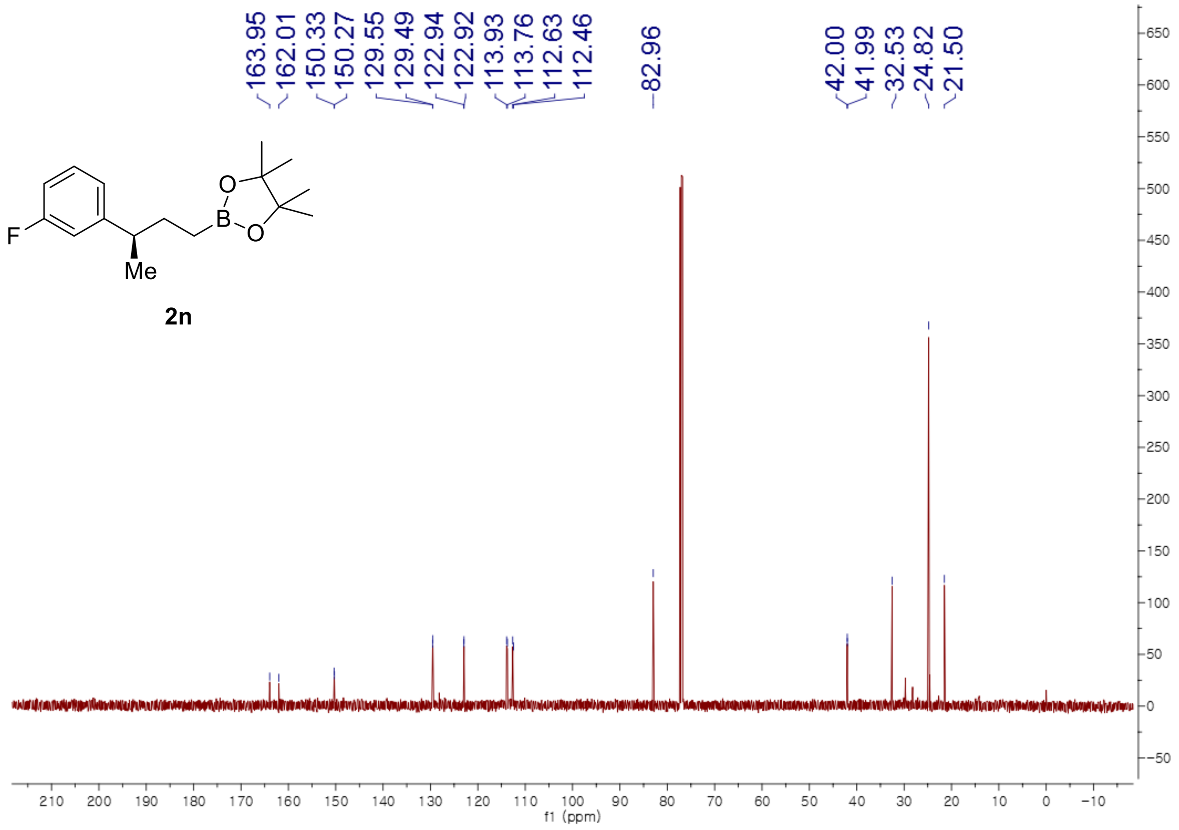
163.95
162.01
150.33
150.27
129.55
129.49
122.94
122.92
113.93
113.76
112.63
112.46
82.96
42.00
41.99
32.53
24.82
21.50
F
O
B
O
Me
2n
f1 (ppm)

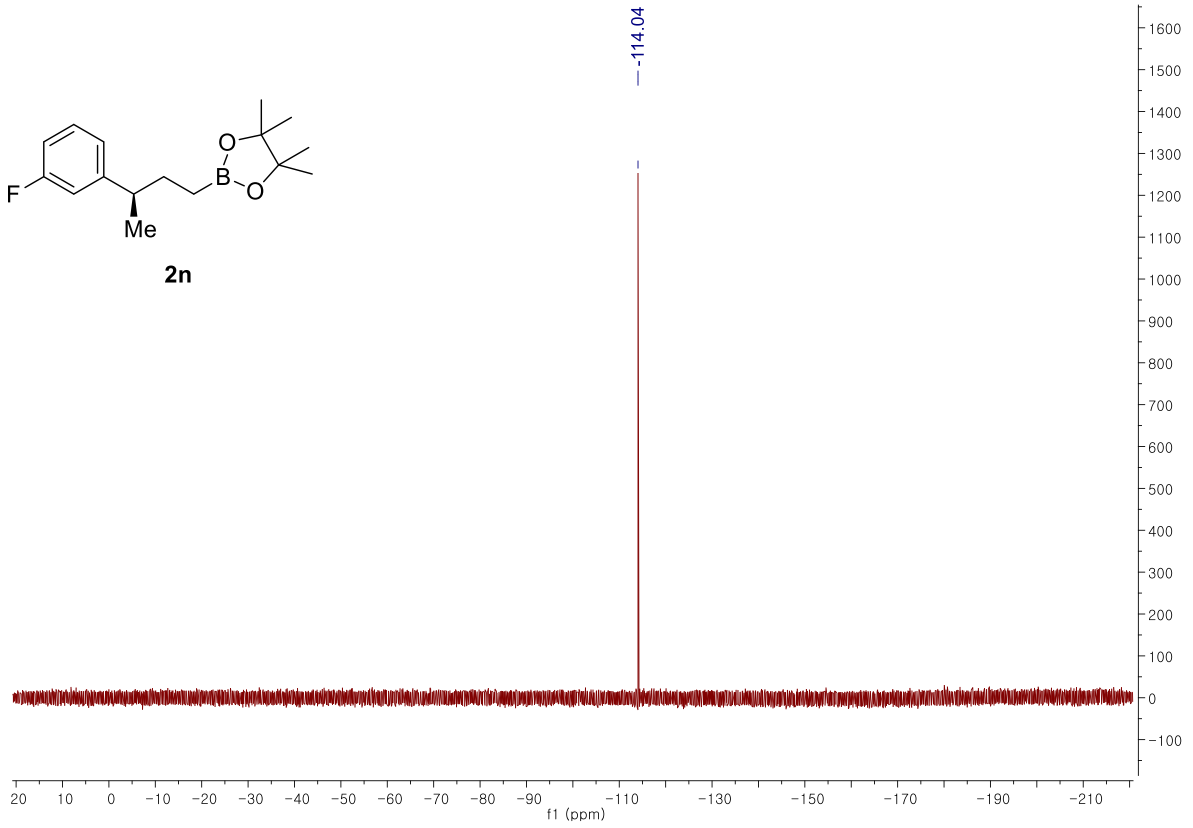

-114.04
F
O
B
O
Me
2n
f1 (ppm)

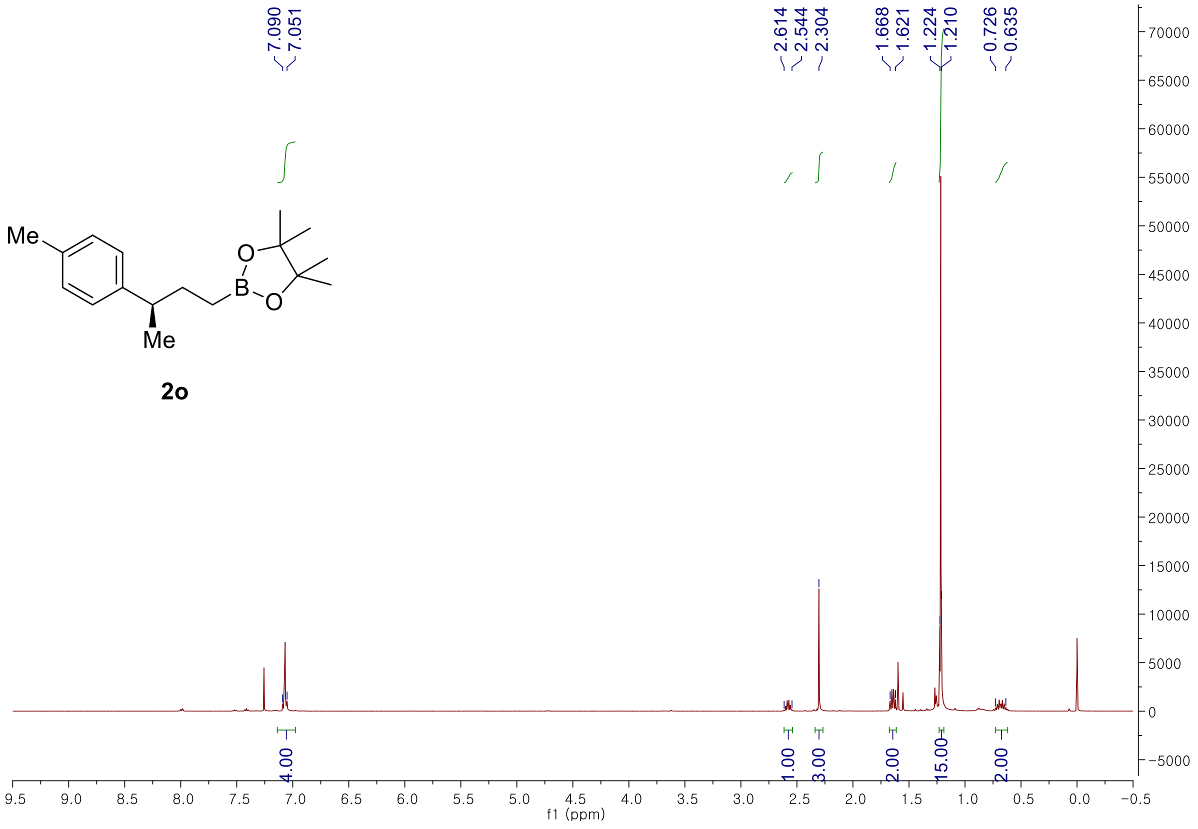

7.090
7.051
2.614
2.544
2.304
1.668
1.621
1.224
1.210
0.726
0.635
Me
O
B
O
Me
2o
4.00
1.00
3.00
2.00
15.00
2.00
f1 (ppm)

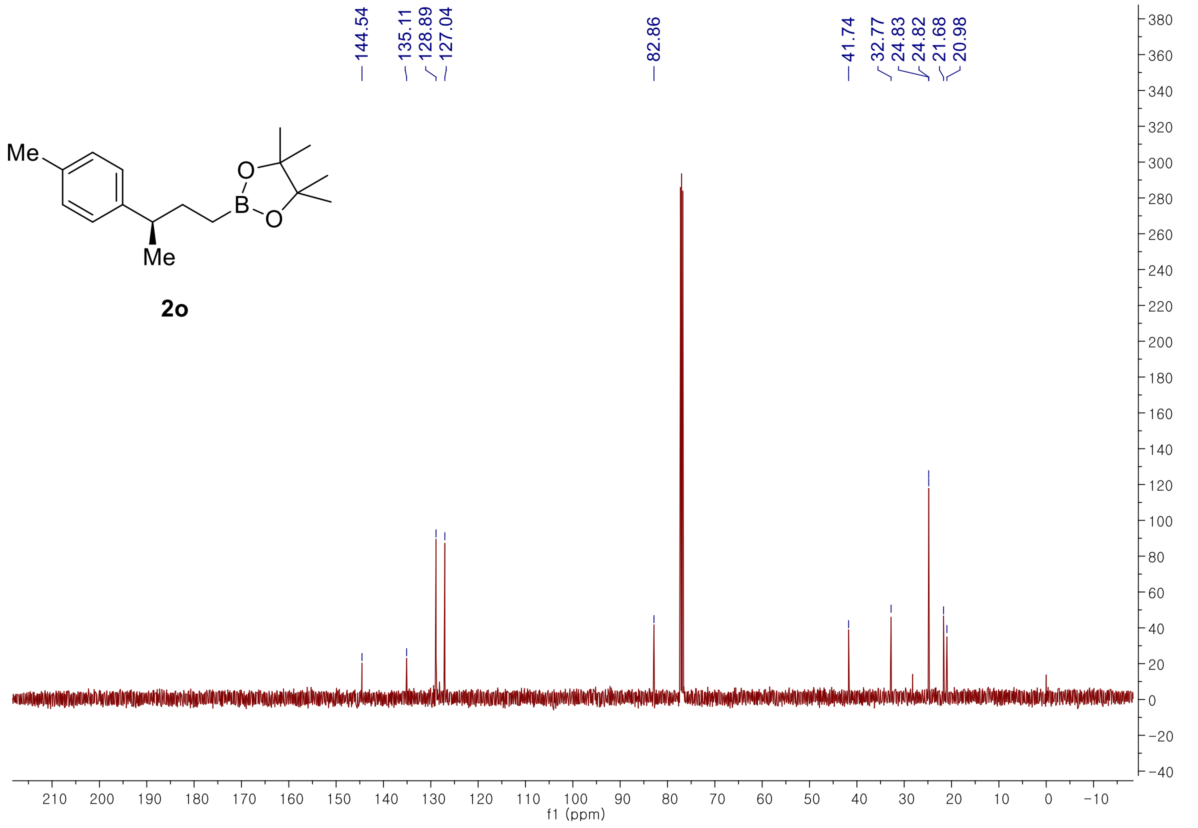

Me
O
B
O
Me
144.54
135.11
128.89
127.04
82.86
41.74
32.77
24.83
24.82
21.68
20.98
f1 (ppm)

2o

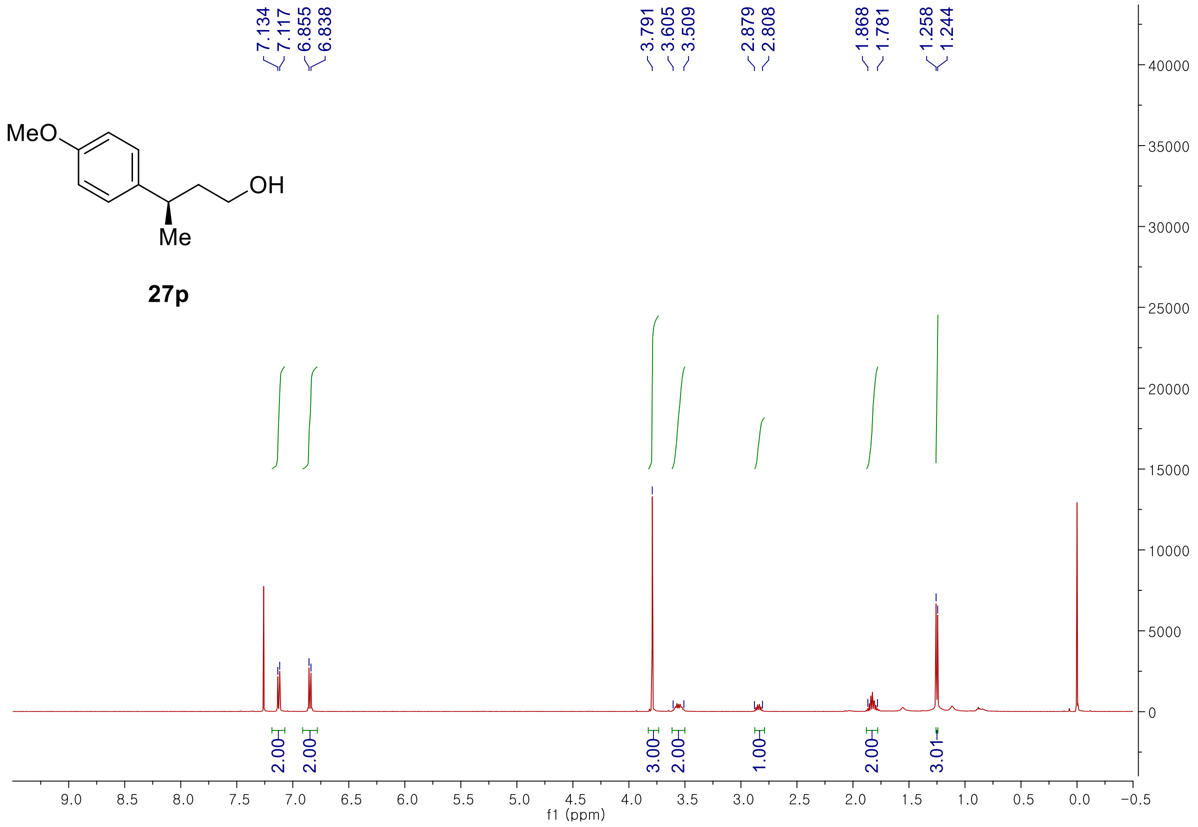

MeO
OH
Me
7.134
7.117
6.855
6.838
3.791
3.605
3.509
2.879
2.808
1.868
1.781
1.258
1.244
2.00
2.00
3.00
2.00
1.00
2.00
3.01
f1 (ppm)

27p

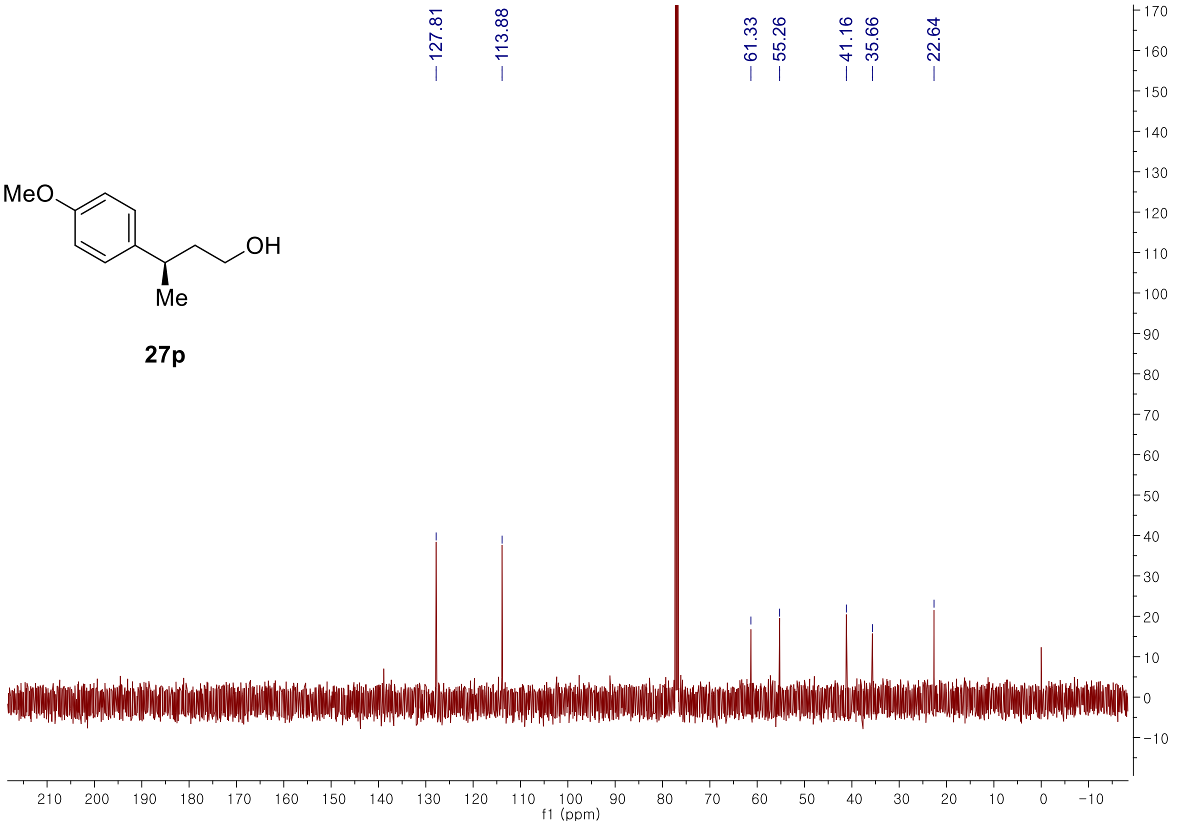

127.81
113.88
61.33
55.26
41.16
35.66
22.64
f1 (ppm)

27p

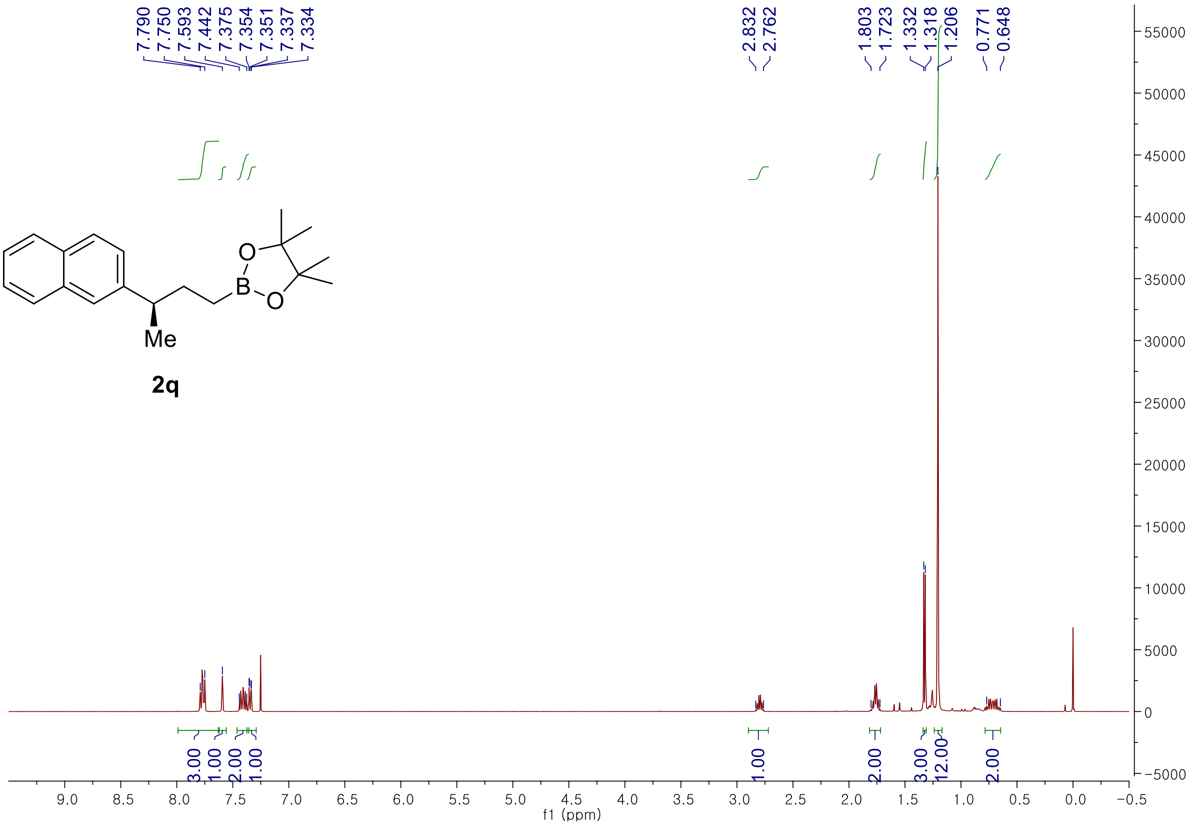

7.790
7.750
7.593
7.442
7.375
7.354
7.351
7.337
7.334
2.832
2.762
1.803
1.723
1.332
1.318
1.206
0.771
0.648
3.00
1.00
2.00
1.00
1.00
2.00
3.00
12.00
2.00
f1 (ppm)

2q

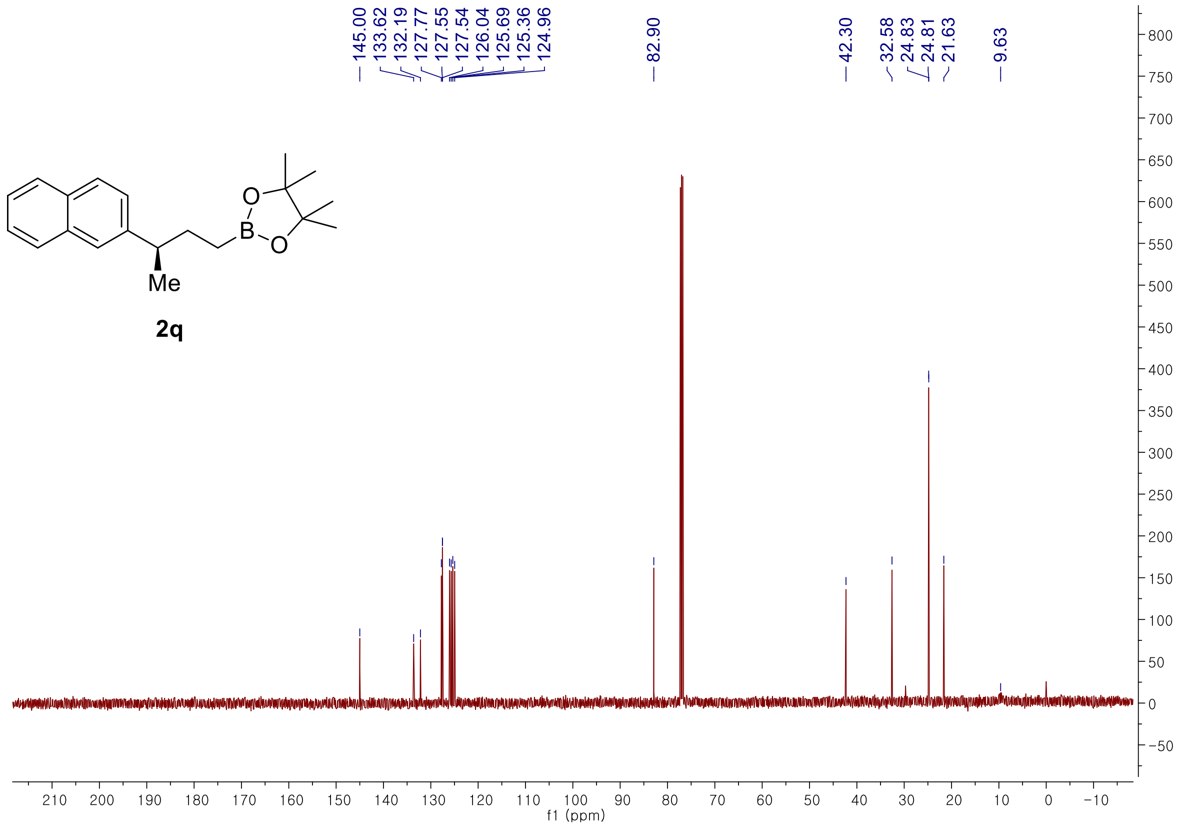
145.00
133.62
132.19
127.77
127.55
127.54
126.04
125.69
125.36
124.96
82.90
42.30
32.58
24.83
24.81
21.63
9.63
Me
O
B
O
f1 (ppm)

2q

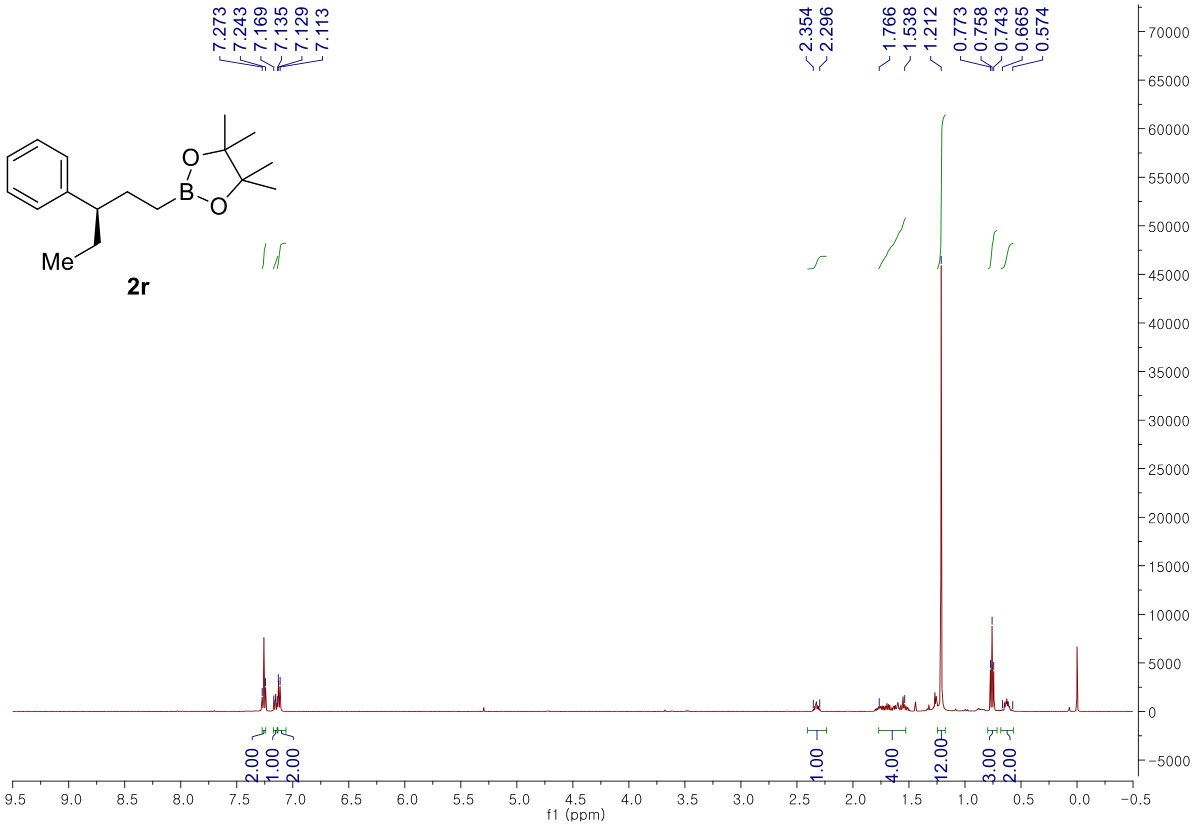
7.273
7.243
7.169
7.135
7.129
7.113
2.354
2.296
1.766
1.538
1.212
0.773
0.758
0.743
0.665
0.574
Me
O
B
O
2.00
1.00
2.00
1.00
4.00
12.00
3.00
2.00
f1 (ppm)

2r

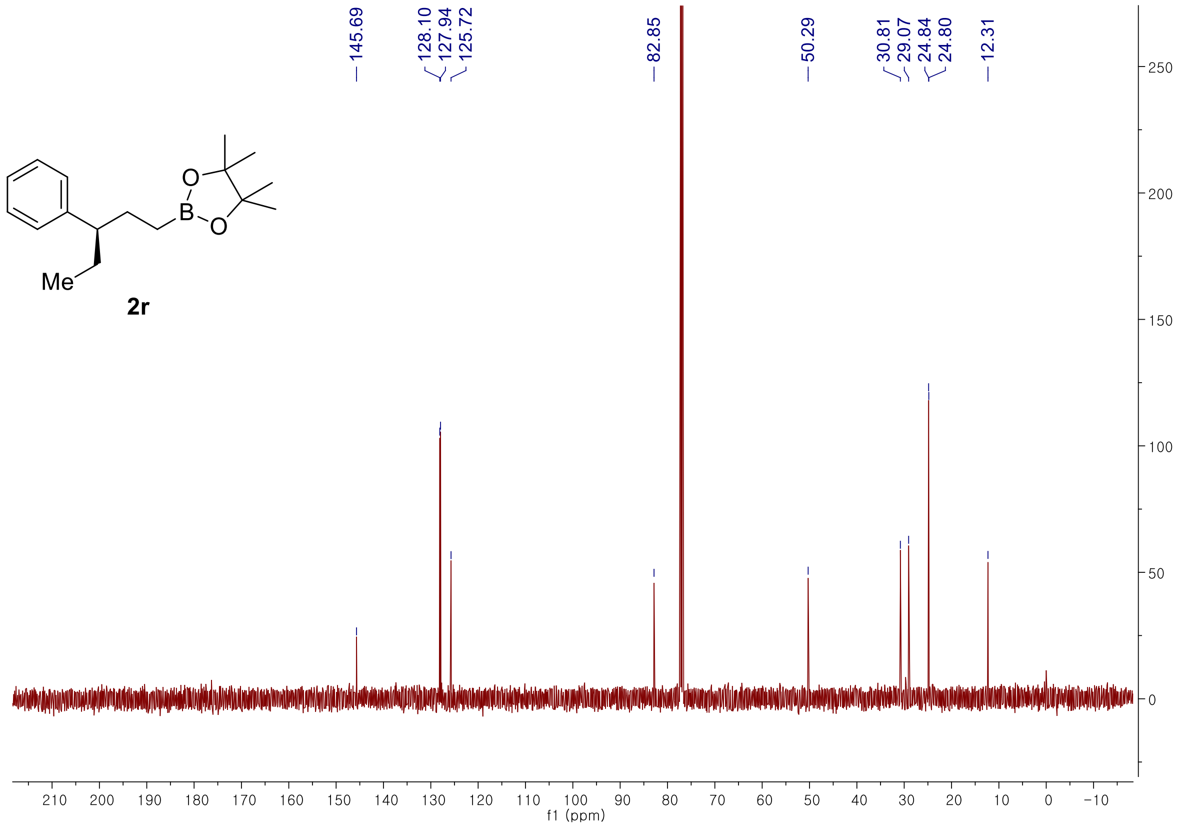

145.69
128.10
127.94
125.72
82.85
50.29
30.81
29.07
24.84
24.80
12.31
O
B
O
Me
2r
f1 (ppm)

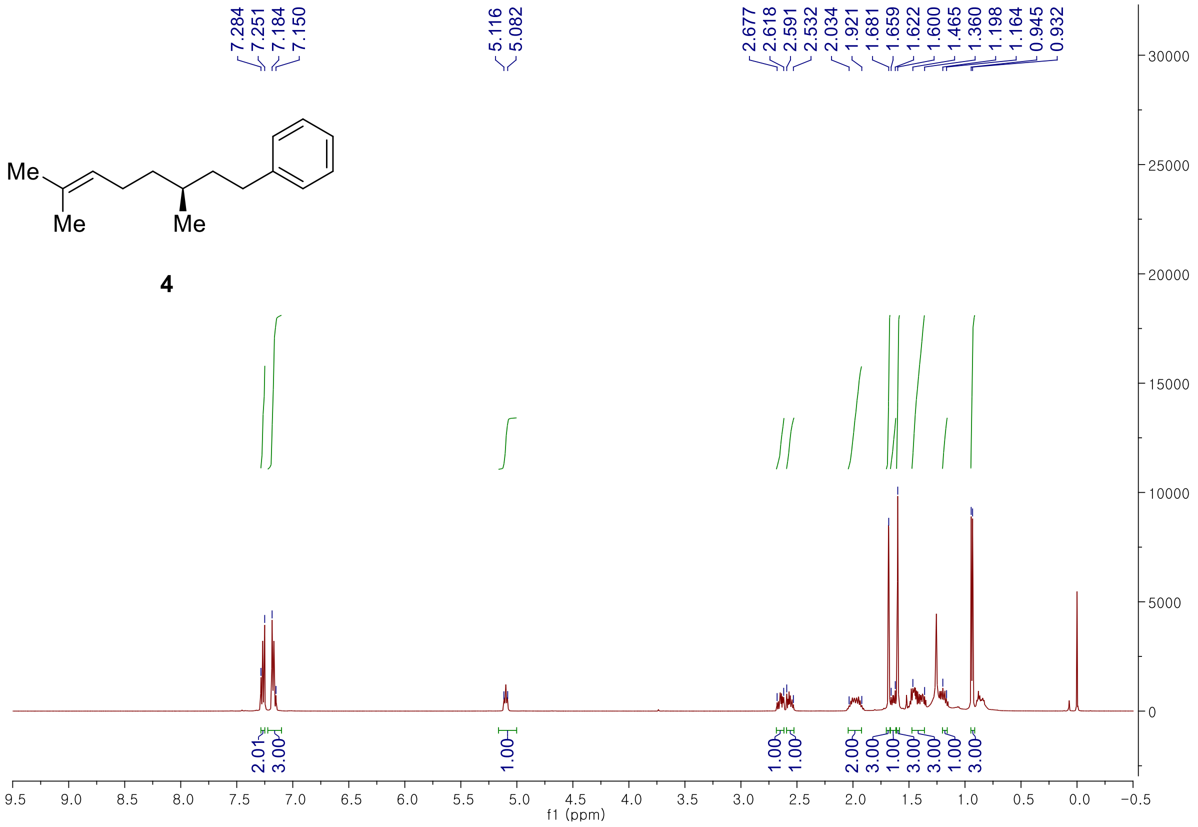

7.284
7.251
7.184
7.150
5.116
5.082
2.677
2.618
2.591
2.532
2.034
1.921
1.681
1.659
1.622
1.600
1.465
1.360
1.198
1.164
0.945
0.932
Me
Me
Me
4
2.01
3.00
1.00
1.00
1.00
2.00
3.00
1.00
3.00
3.00
1.00
3.00
f1 (ppm)

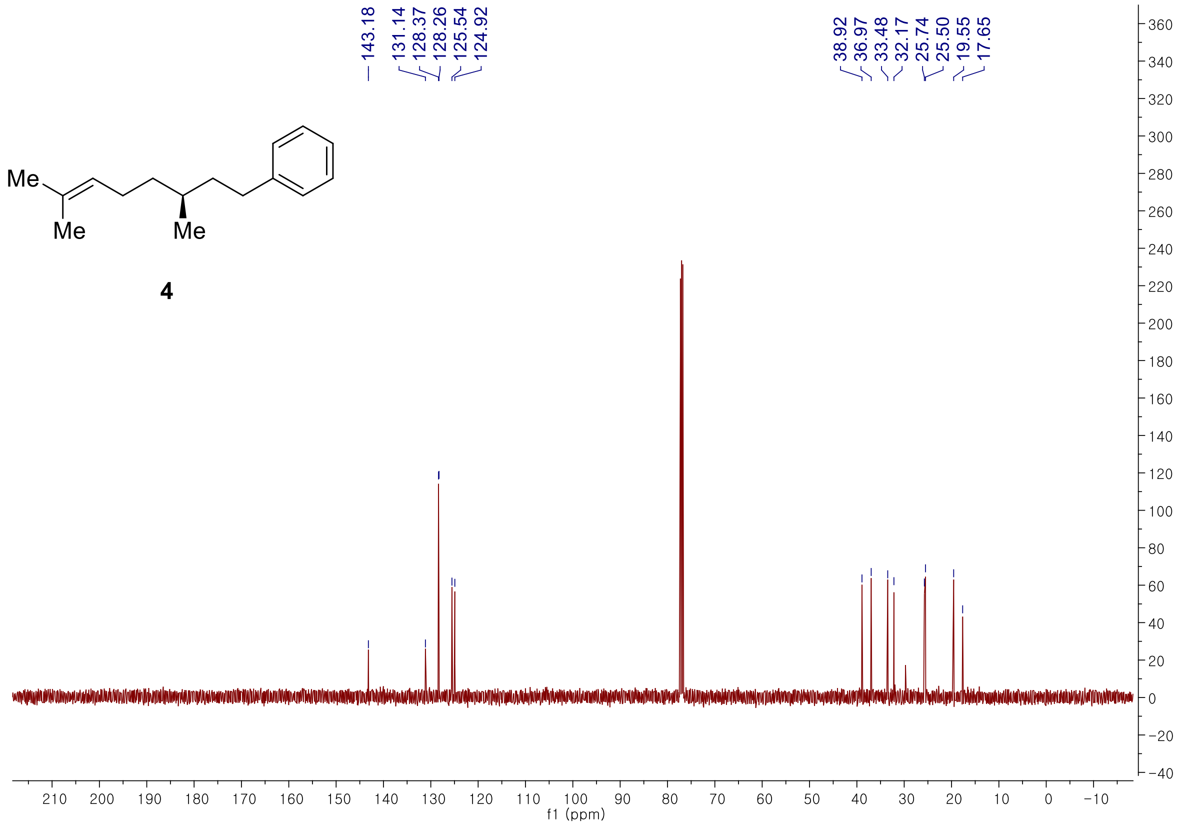
143.18
131.14
128.37
128.26
125.54
124.92
38.92
36.97
33.48
32.17
25.74
25.50
19.55
17.65
Me
Me
Me
4
f1 (ppm)

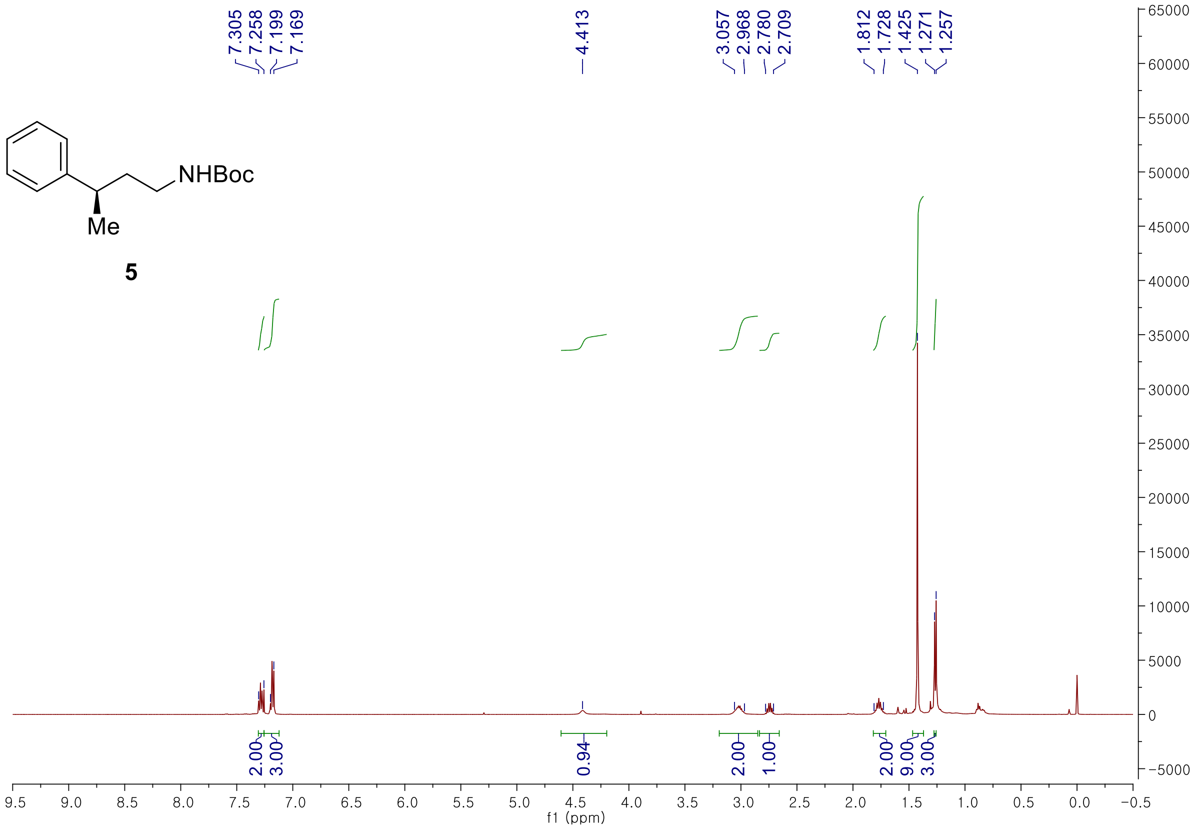
7.305
7.258
7.199
7.169
4.413
3.057
2.968
2.780
2.709
1.812
1.728
1.425
1.271
1.257
NHBoc
Me
5
2.00
3.00
0.94
2.00
1.00
2.00
9.00
3.00
f1 (ppm)

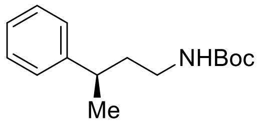

5

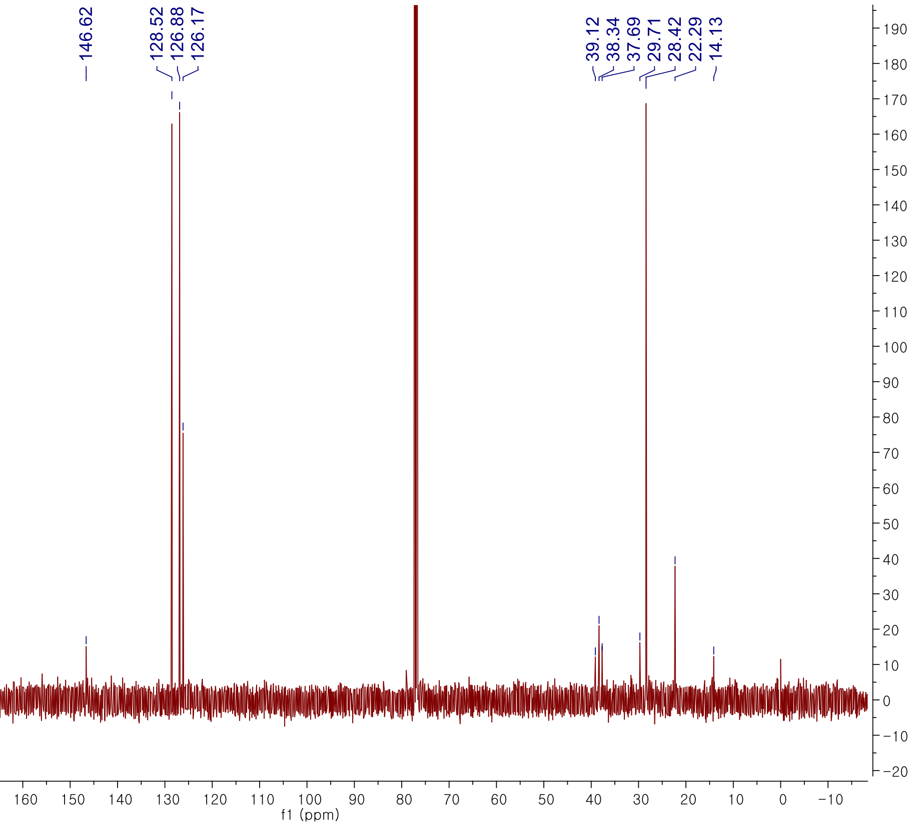

146.62
128.52
126.88
126.17
39.12
38.34
37.69
29.71
28.42
22.29
14.13
f1 (ppm)
